# Supplementary material for: The GALANT trial: study protocol of a randomised placebo-controlled trial in patients with a 68Ga-DOTATATE PET-positive, clinically non-functioning pituitary macroadenoma on the effect of lanreotide on tumour size
Source: BMJ Open. 2020 Aug 13;10(8):e038250. doi: 10.1136/bmjopen-2020-038250 (PMC7430490; doi:10.1136/bmjopen-2020-038250)
Supplement: Supplementary data [file bmjopen-2020-038250supp002.pdf]

Amsterdam UMC
Universitair Medische Centra

INFORMATIEBRIEF VOOR PROEFPERSONEN

GALANT: Het effect van lanreotide op de grootte van niet-functionerende hypofyse macroadenomen (NFMA)

Officiële titel: Een gerandomiseerde, placebo-gecontroleerde studie onder patiënten met **Ga**llium-68 DOTATATE PET/CT positieve, klinisch niet-functionerende hypofyse macroadenomen (NFMA) naar het effect van **Lan**reotide autosolution op **T**umorgrootte (**GALANT**)

Geachte heer/mevrouw,

Wij vragen u vriendelijk om mee te doen aan een medisch-wetenschappelijk onderzoek. Wij hebben u benaderd omdat u op dit moment onder behandeling bent bij de afdeling Endocrinologie van het AMC vanwege een adenoom (tumor) van de hypofyse. U beslist zelf of u wilt meedoen. Voordat u de beslissing neemt, is het belangrijk om meer te weten over het onderzoek. Lees deze informatiebrief daarom rustig door en bespreek het met partner, vrienden en/of familie. Bekijk ook de bijgevoegde brochure 'Medisch-wetenschappelijk onderzoek', daarin staat veel algemene informatie. Hebt u na het lezen van de informatie nog vragen? Dan kunt u terecht bij de arts-onderzoeker of de onafhankelijke arts. In **bijlage A**, pagina 7, vindt u de contactgegevens.

1. Wat is het doel van het onderzoek?

Het doel van dit onderzoek is het testen van het medicijn lanreotide als behandeling van bepaalde hypofyse tumoren. De hypofyse is een klein orgaan dat vlak onder de hersenen hangt, achter de neusbrug. De hypofyse heeft een centrale rol in de hormoonhuishouding van het lichaam. Een hypofyse adenoom is een goedaardige tumor of gezwel van de hypofyse. Als dit adenoom zelf geen hormonen produceert wordt dit "niet-functionerend" genoemd. Adenomen groter dan 1 centimeter worden macroadenomen genoemd. Door groei van het adenoom kan het gezonde deel van de hypofyse in verdrukking komen. Hierdoor kunnen hormonen uitvallen waarvoor aanvulling nodig is met hormoontherapie ("substitutie"). Als het adenoom te groot wordt, en daardoor op de oogzenuw drukt, is een operatie noodzakelijk. Helaas zien we dat hierna het adenoom toch vaak terug komt. Op dit moment is behandeling met een medicijn nog niet mogelijk. De Endocrinologie afdelingen van het AMC en het VU Medisch Centrum (VUmc) in Amsterdam zijn een gezamenlijk onderzoek gestart naar de werkzaamheid van het medicijn lanreotide bij patiënten met een niet-functionerend hypofyse macroadenoom. Wij hopen dat door dit medicijn het adenoom langzamer groeit of zelfs slinkt. Dit zou betekenen dat een operatie niet of pas later nodig is. Het onderzoek wordt verricht met financiële steun van Ipsen, het farmaceutische bedrijf dat lanreotide produceert.

2. Wat is lanreotide precies?

Lanreotide is een eiwit dat lijkt op het natuurlijke hormoon somatostatine. Het behoort tot de groep "antigroei hormonen". De merknaam van lanreotide is Somatuline AutoSolution. Dit zijn twee namen voor hetzelfde medicijn. Het wordt toegediend als een injectie. Lanreotide wordt al gebruikt voor de behandeling van hypofyse adenomen die groeihormoon of schildklier stimulerend hormoon aanmaken. Uit een aantal onderzoeken bleek dat ongeveer een derde van de niet-functionerende hypofyse adenomen ook gevoelig kan zijn voor lanreotide, zelfs als ze geen hormoon aanmaken. Deze onderzoeken waren echter

klein en er was geen vergelijking met een controlegroep. Hierdoor zijn de uitkomsten niet betrouwbaar genoeg.

3. Hoe wordt het onderzoek uitgevoerd?

*Zie ook het overzicht in **bijlage B***

Soort onderzoek

Het is een gerandomiseerd, dubbelblind, placebo-gecontroleerd onderzoek. Dit betekent dat een loting bepaalt in welke behandelgroep u komt (randomisatie). De ene groep krijgt het medicijn lanreotide en de andere groep krijgt een placebo (een "nepbehandeling") in de vorm van een onschadelijke zoutoplossing. Beide middelen worden als injectie toegediend. Dubbelblind betekent dat u en ook de onderzoekers niet weten welke behandeling u krijgt. Er vindt eerst een screening plaats om te zien of u mee kunt doen aan de loting en de behandeling.

De eerste bezoeken: screening

Indien u interesse heeft om mee te doen aan het onderzoek volgt een uitnodiging voor het eerste bezoek in het AMC. Hier zullen we eerst bekijken of deelname mogelijk is. Allereerst zal de onderzoeker wat algemene informatie vastleggen, zoals doorgemaakte en huidige ziektes, medicatiegebruik, allergieën etc. Wij vragen u ook een vragenlijst in te vullen over uw kwaliteit van leven. Hiernaast vinden tijdens het eerste bezoek een kort lichamelijk onderzoek en bloedafname (8 buisjes, 36 milliliter bloed) plaats. De MRI-scan van de hersenen wordt herhaald als het nodig is om de precieze grootte van het adenoom opnieuw te meten. Alleen adenomen groter dan 1 centimeter kunnen onderzocht worden. De genoemde testen en controles maken deel uit van uw reguliere behandeling, met uitzondering van de vragenlijst.

Als blijkt dat deelname mogelijk is volgt er nog een tweede screening. Deze tweede screening bestaat uit een speciale PET/CT scan. Deze scan laat zien of het adenoom bepaalde eigenschappen heeft waardoor lanreotide aan het adenoom kan binden. Zo ja, dan bestaat de kans dat het adenoom reageert op een behandeling met lanreotide. Alleen in dat geval kunt u meedoen aan het onderzoek. De scan vindt plaats in het AMC. Als dat mogelijk is al op dezelfde dag, maar hier kan ook een tweede bezoek voor nodig zijn. Voor de scan krijgt u een kleine hoeveelheid van een licht radioactieve stof (een radionuclide) toegediend via een infuus. Meer informatie over de PET/CT scan vindt u in **bijlage D**.

De verdere uitvoering en duur van het onderzoek

De loting vindt plaats als na de screening blijkt dat u mee kunt doen aan het onderzoek. Hierna starten de injecties. Elke 4 weken (28 dagen) krijgt u een injectie. Deze wordt diep onder de huid van de bil toegediend. In totaal krijgt u 18 injecties van óf lanreotide óf placebo. In principe worden de injecties elke 4 weken in het AMC toegediend door een gespecialiseerde endocrinologie verpleegkundige. Als dit voor u ver reizen is of als het niet goed uitkomt om overdag naar het AMC te komen, dan kunnen de injecties ook thuis of zelfs op werk toegediend worden. Dit wordt gedaan door verpleegkundigen van de Somacare thuiszorgservice van Eurocept Homecare. U wordt als deelnemer aan deze studie bij hen aangemeld zodat zij uw gegevens hebben en contact kunnen opnemen over de toediening.

Tijdens het onderzoek komt u verder elk halfjaar naar het AMC voor een controle-bezoek aan de arts-onderzoeker, in totaal drie keer. Hier herhalen we het lichamelijk onderzoek, de bloedafname en de vragenlijst. Ook vragen we naar mogelijke bijwerkingen of andere problemen. Het eerste en tweede controle-bezoek vinden plaats op de dag van een injectie, zodat deze tegelijk in het AMC kan plaatsvinden. Het derde en laatste controle-bezoek vindt plaats ná de laatste injectie. Rond het eerste en rond het laatste

bezoek wordt de MRI-scan van de hersenen herhaald om de grootte van het adenoom te bekijken. De totale duur van het onderzoek is ongeveer anderhalf jaar (18 maanden). In totaal zijn er minimaal 4 en maximaal 21 bezoeken aan het ziekenhuis. Dit hangt af van waar de injecties toegediend worden. In **bijlage B** vindt u een handig overzicht van het onderzoek.

4. Wat wordt er van u verwacht?

Indien u meedoet aan het onderzoek vragen wij u vaker naar het ziekenhuis te komen dan gebruikelijk. Hoe vaak u precies moet komen hangt af van waar de injecties worden toegediend. Als u de injecties in het AMC laat toedienen dan zijn deze bezoeken van korte duur. Om het onderzoek goed te kunnen uitvoeren is het belangrijk dat u zich houdt aan de geplande afspraken in het ziekenhuis en thuis. U kunt contact opnemen met de arts-onderzoeker of met Eurocept Homecare als u een afspraak wilt wijzigen.
Het onderzoek heeft geen invloed op uw dagelijkse activiteiten of werk. Er zijn geen beperkingen wat eten en drinken betreft. Lanreotide kan soms effect hebben op andere medicijnen. Daarom is het belangrijk dat de onderzoeker voor de start van het onderzoek uw medicatie controleert. Als deelname mogelijk is mag u uw eigen medicatie blijven gebruiken. Het is wel belangrijk de onderzoeker in te lichten voordat u nieuwe medicijnen gaat gebruiken tijdens de studie.

Voor vrouwelijke deelnemers
Geeft u borstvoeding, bent u zwanger of van plan dit te worden? Dan kunt u niet meedoen aan dit onderzoek. Bent u in de vruchtbare leeftijd? Dan is het van belang om tijdens het onderzoek adequate voorbehoedsmiddelen (anticonceptie) te gebruiken. Er is namelijk nog te weinig bekend over de gevolgen van lanreotide op het ongeboren kind. Wordt u toch zwanger tijdens het onderzoek? Neem dan contact op met de onderzoeker of uw arts. De zwangerschap wordt dan extra gecontroleerd.

5. Heeft meedoen invloed op de reguliere behandeling die u krijgt?

Meedoen heeft geen invloed op de reguliere behandeling van uw niet-functionerende hypofyse adenoom. Deze bestaat gewoonlijk uit controle van klachten, de grootte van het adenoom en laboratoriumbepalingen; naast eventuele hormoontherapie om tekorten aan te vullen (“substitutie”). Deze controles en therapie vinden ook plaats tijdens het onderzoek. Bepaalde klachten kunnen passen bij groei van het adenoom. Indien deze optreden tijdens het onderzoek krijgt u, conform de reguliere behandeling, een extra MRI-scan van de hersenen. Als blijkt dat het adenoom geopereerd moet worden stopt het onderzoek. Dit heeft geen consequenties voor uw verdere behandeling.

6. Welke bijwerkingen en ongemakken kunt u verwachten?

Lanreotide
Zoals alle medicijnen kan ook lanreotide bijwerkingen veroorzaken, al heeft lang niet iedereen hier last van. Hieronder staan de meest voorkomende bijwerkingen. Deze komen bij ongeveer 10% (één op de tien) van de mensen voor:

- Maag-darmklachten zoals diarree, buikpijn en misselijkheid (vooral na de eerste injectie)
- Vorming van galstenen (meestal zonder klachten)
- Huidreacties op de injectieplaats zoals zwelling, pijn en jeuk

Andere bijwerkingen komen minder vaak voor of zijn zeldzaam. Ook bestaat er altijd een kans op nog onbekende bijwerkingen. Meer informatie over de bijwerkingen vindt u in **bijlage C**. Daarin staat ook met wie u contact moet opnemen bij een mogelijk ernstige bijwerking.

MRI scans (reguliere behandeling)
Een MRI-scan werkt met een magneet, hierbij wordt u niet blootgesteld aan straling. Een MRI-scan kan niet plaatsvinden als zich in of op het lichaam kleine metaaldeeltjes bevinden, zoals een metaalsplinter in het oog, implantaten in de oren of metalen vaatclips in het hoofd. Ook kan dit niet bij geïmplanteerde elektronische apparatuur, zoals een pacemaker.

Bij de MRI-scans krijgt u een contrastmiddel toegediend via een bloedvat in de arm. Dit middel bevat geen jodium. In sommige gevallen ontstaan door het contrastmiddel kortdurend onschuldige bijwerkingen zoals hoofdpijn en misselijkheid. Als u bij een eerdere MRI-scan een allergische reactie hebt gehad kunt u niet meedoen met het onderzoek.

PET/CT scan en de radionuclide
Voor de PET/CT krijgt u zoals we eerder schreven een radionuclide toegediend, deze heet “Gallium-68 DOTATATE”. (Allergische) reacties hierop zijn zeldzaam. Het prikken van het infuus voor de toediening kan onprettig zijn en een blauwe plek veroorzaken.

De radionuclide is licht radioactief. Het stofje blijft net lang genoeg radioactief om de afbeeldingen te kunnen maken. Hierbij wordt u blootgesteld aan een kleine hoeveelheid straling. De geschatte stralingsbelasting van dit onderzoek is 3.1 mSv (millisievert). Uitgebreide informatie hierover vindt u in **bijlage D**.

7. Wat zijn mogelijke voor- en nadelen van deelname aan dit onderzoek?
Door resultaten uit eerder onderzoek is de verwachting dat lanreotide de groei van bepaalde hypofyse adenomen remt, maar zeker is dat niet. Het is daarom niet te zeggen of u voordeel heeft van deelname aan het onderzoek. Het huidige onderzoek zal het effect van lanreotide nauwkeuriger bestuderen en het vergelijken met een controlegroep. De kans om deel van die controlegroep te zijn en placebo injecties te krijgen is 50% (één op twee). Dit is een nadeel, ook vanwege de vele afspraken thuis of bezoeken aan het ziekenhuis om de injecties te ontvangen. Andere nadelen zijn de extra PET/CT scan met de bijbehorende straling en de mogelijke bijwerkingen of ongemakken van de injecties zelf. Tijdens de onderzoeksperiode wordt 4 keer een kleine hoeveelheid bloed afgenomen (telkens 36 milliliter). Dit gebeurt gewoonlijk ook tijdens de reguliere behandeling. Als er onverwacht afwijkingen uit het bloedonderzoek of de scans komen, zullen we dit altijd met u bespreken. Als u dit niet wilt, kunt u niet aan het onderzoek meedoen.

8. Wat gebeurt er als u niet wenst deel te nemen aan dit onderzoek of wilt stoppen?
Deelname aan het onderzoek is geheel vrijwillig. Als u besluit niet mee te doen, hoeft u niet te zeggen waarom. Als u wel meedoet, kunt u zich altijd bedenken en toch stoppen, ook tijdens het onderzoek. Als u besluit niet mee te doen of te stoppen, dan worden uw eigen behandeling en controles gewoon voortgezet. Tussentijds stoppen heeft geen risico’s voor uw gezondheid. Deelname kan ook door de onderzoeker worden beëindigd. Bijvoorbeeld als uw veiligheid in gevaar is.

Als er tijdens het onderzoek nieuwe informatie naar voren komt die belangrijk is voor u, laat de onderzoeker dit altijd aan u weten. U mag dan zelf beslissen of u blijft meedoen. Extra informatie over deelname, stoppen en uw rechten als proefpersoon kunt u vinden in de bijgevoegde brochure.

Boertien TM, *et al. BMJ Open* 2020; 10:e038250. doi: 10.1136/bmjopen-2020-038250

9. Wat gebeurt er met uw gegevens?

Privacy

Voor dit onderzoek worden uw medische en persoonlijke gegevens op verschillende manieren verzameld en gebruikt. Al uw gegevens blijven vertrouwelijk.

Hieronder staat waar en waarom uw persoonsgegevens verzameld worden:

- Bij de thuiszorgservice van Eurocept Homecare worden uw persoonsgegevens geregistreerd voor het inplannen en toedienen van injecties thuis.
- In een apart studiedossier in het AMC slaat de arts-onderzoeker uw contactgegevens op zodat zij u kan bereiken als dat nodig is.

Hiernaast worden alle gegevens voor het onderzoek verzameld en opgeslagen met behulp van een studiecode. Elke proefpersoon krijgt een eigen studiecode die de persoonsgegevens zoals uw naam vervangt. Welke code u heeft is alleen bekend bij het onderzoeksteam. Bij bespreken van en in rapporten over het onderzoek wordt alleen de studiecode gebruikt.

Inzien van onderzoeksgegevens

Om te controleren of het onderzoek degelijk en betrouwbaar wordt uitgevoerd, mogen bepaalde mensen wel uw gegevens zonder code inzien. Naast het onderzoeksteam zijn dit een controleur van het AMC en de Inspectie voor de Gezondheidszorg. Zij zijn verplicht om uw gegevens geheim te houden. Als vertegenwoordigers van Ipsen, de financier van het onderzoek, het onderzoek moeten controleren dan mogen zij de gegevens alleen met de studiecode inzien.

Bewaren van gegevens

Alle onderzoeksgegevens moeten wettelijk 15 jaar bewaard worden. Bij het ondertekenen van het toestemmingsformulier geeft u toestemming voor het verzamelen, inzien en bewaren van uw medische en persoonsgegevens. Algemene informatie over omgang met gegevens vindt u ook in de bijgevoegde brochure.

Bewaren van bloed

Gedurende de onderzoeksperiode wordt als reguliere controle een paar keer bloed afgenomen. Een deel hiervan word langer bewaard dan normaal, ook met gebruik van de code. Dit gebeurt voor het geval er later aanvullende bepalingen nodig blijken. Het bewaarde bloed wordt alleen voor het huidige onderzoek gebruikt. Het wordt vernietigd zodra het onderzoek in zijn geheel is voltooid. Dit houdt in dat alle patiënten die meedoen het onderzoek hebben afgerond en dat de resultaten kunnen worden gepubliceerd. In totaal zal het niet langer dan 5 jaar bewaard worden. Voor het bewaren van uw bloed geeft u toestemming via het toestemmingsformulier.

10. Bent u verzekerd wanneer u aan het onderzoek meedoet?

Voor iedereen die meedoet aan dit onderzoek is een verzekering afgesloten. De verzekering dekt schade als gevolg van het onderzoek. Dit geldt voor schade die naar boven komt tijdens het onderzoek, of binnen vier jaar na het einde van het onderzoek. In **bijlage E** vindt u de verzekerde bedragen, de uitzonderingen en de contactgegevens van de verzekeraar.

11. Wordt uw huisarts en/of behandelend specialist geïnformeerd bij deelname?

Wij laten uw huisarts en uw behandelend internist-endocrinoloog altijd schriftelijk weten dat u meedoet aan het onderzoek. Hiervoor geeft u toestemming via het toestemmingsformulier. Als u geen toestemming geeft, is meedoen niet mogelijk.

12. Is er een vergoeding wanneer u besluit deel te nemen?

De studiemedicatie, extra bezoeken en PET/CT scan kosten u niks. U wordt niet betaald voor het meedoen aan dit onderzoek. Wel krijgt u een vergoeding voor uw reiskosten.

13. Welke medisch-ethische toetsingscommissie heeft dit onderzoek goedgekeurd?

De medisch-ethische toetsingscommissie (METC) van het AMC heeft dit onderzoek goedgekeurd. Meer informatie over goedkeuring vindt u in de algemene brochure.

14. Wilt u verder nog iets weten?

U hoeft niet direct te beslissen of u mee wilt doen. De bedenktijd is ongeveer een week. Bij vragen kunt u contact opnemen met de arts-onderzoeker, Tessel Boertien. Voor onafhankelijk advies over meedoen aan dit onderzoek kunt u terecht bij de onafhankelijke arts, dr. M.R. Soeters, internist-endocrinoloog. Hij weet van het onderzoek af maar is zelf niet betrokken. Naast de algemene brochure kunt u ook op de website van het AMC veel informatie vinden, onder andere over het meedoen aan onderzoek en rechten & plichten als patiënt. Alle contactgegevens vindt u in **bijlage A**.

Dank voor uw aandacht en met vriendelijke groet,

Drs. Tessel M. Boertien, arts-onderzoeker en projectleider
Prof. Dr. E. Fliers, internist-endocrinoloog en hoofdonderzoeker AMC

<u>Bijlagen bij deze informatiebrief</u>:

- Los: Brochure 'Medisch-wetenschappelijk onderzoek. Algemene informatie voor de proefpersoon' (versie september 2014)
- Bijlage A: Contactgegevens en informatie per centrum
- Bijlage B: Overzicht van het onderzoek
- Bijlage C: Bijwerkingen lanreotide – Somatuline AutoSolution
- Bijlage D: Extra informatie over PET/CT en stralenbelasting
- Bijlage E: Verzekeringstekst
- Bijlage F: Toestemmingsformulier

BIJLAGE A: CONTACTGEGEVENS EN INFORMATIE PER CENTRUM

<u>Projectleider & eerste contactpersoon</u>
Drs. Tessel M. Boertien, arts-onderzoeker endocrinologie AMC & VUmc
e-mail **t.m.boertien@amsterdamumc.nl**
Tel. **020 566 0172** / 020 566 6071 (secr.)

Postadres onderzoek: Academisch Medisch Centrum, t.a.v. T.M. Boertien
Afdeling Endocrinologie & Metabolisme, kamer K2-283
Meibergdreef 9, 1105 AZ Amsterdam

Onafhankelijk arts AMC
Dr. M.R. Soeters, internist-endocrinoloog
e-mail m.r.soeters@amsterdamumc.nl
Tel. 020 566 6071

Informatie Academisch Medisch Centrum (AMC)
Bezoekadres: Meibergdreef 9
1105 AZ Amsterdam
Algemeen nummer: 020 566 9111
Nucleaire geneeskunde: 020 566 2775 (7:30-17:00)

Bij <u>acute problemen buiten kantoortijden</u> kunt u via het algemene telefoonnummer (020 566 9111) 24 uur per dag worden doorverbonden naar de dienstdoende internist-endocrinoloog. Vermeld altijd dat u meedoet aan deze studie.

Hoofdonderzoeker
Prof. dr. E. Fliers, internist-endocrinoloog
e-mail e.fliers@amsterdamumc.nl
Tel. 020 566 6071

Endocrinologie verpleegkundigen
Moniek Gent-Houben en Shariefa Peters-Amanatkhan
e-mail fk.endocrinologie@amsterdamumc.nl
Tel. 020 566 2990

Afdeling Patiëntenvoorlichting en Klachtenopvang AMC
Bij de afdeling patiëntenvoorlichting en klachtenopvang in het AMC kunt u terecht met algemene vragen en informatie, bijvoorbeeld over het meedoen aan onderzoek. Ook kunt u hier terecht indien u ontevreden bent over de gang van zaken of een klacht wilt indienen. De medewerkers verzorgen de eerste klachtenopvang en informeren over de klachtenprocedure. Op de website van het AMC is veel van de informatie terug te vinden: https://www.amc.nl/web/ik-heb-een-afspraak-1/rechten-en-plichten.htm

Locatie binnen AMC: begane grond van het polikliniekgebouw (A0)
Openingstijden: maandag t/m vrijdag van 9:00 tot 12:30 uur

Tel.: 020 566 3355
e-mail: patientenvoorlichting@amc.nl
klachtenfunctionaris@amc.nl
Postadres: Academisch Medisch Centrum, t.a.v. patiëntenvoorlichting, A0-144
Postbus 22660, 1100 DD Amsterdam

Contactgegevens Eurocept Homecare

Tel.: 035 528 8375 (ook buiten kantooruren)
e-mail galantstudie@eurocept.nl

BMJ Publishing Group Limited (BMJ) disclaims all liability and responsibility arising from any reliance placed on this supplemental material which has been supplied by the author(s)

BIJLAGE B: OVERZICHT VAN HET ONDERZOEK

Hieronder staat een schematisch overzicht van het onderzoek. De studie bezoeken met de **dikgedrukte** nummers vinden in ieder geval plaats. In dit voorbeeld overzicht staat de PET/CT scan gepland tijdens een apart bezoek (nr. 2). Het kan zijn dat deze scan al tijdens bezoek nr. 1 plaatsvindt. De 7[e] en 13[e] injectie vinden plaats op de dag dat u ook naar het AMC komt. Het totale aantal studie bezoeken hangt af van waar de overige injecties plaatsvinden: thuis of in het AMC. Daarom staan die bezoeknummers tussen haakjes. Als alle injecties in het AMC plaatsvinden zijn er in totaal 21 bezoeken.

LOTING ↓

Tijd → / Studie bezoek →	start **1**	**2**	(3 – 8)	6 mnd. **3** (9)	(10 – 14)	12 mnd. **4** (15)	(16 – 20)	18 mnd. **5** (21)	EINDE
Locatie →	AMC	AMC	AMC/thuis	AMC	AMC/thuis	AMC	AMC/thuis	AMC	
Duur in minuten →	90	90	15	90	15	60	15	75	

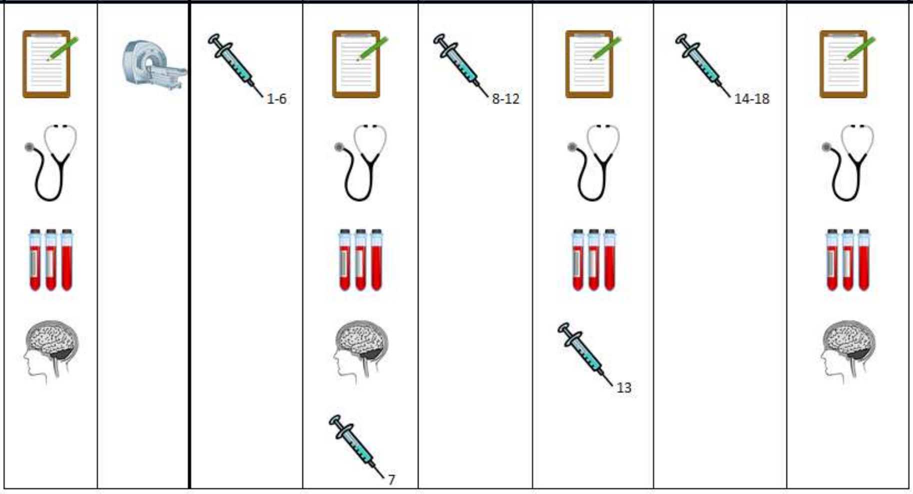

= gesprek en kwaliteit van leven vragenlijst

= kort lichamelijk onderzoek (bloeddruk, pols, lengte, gewicht; uitgebreider op geleide van klachten)

= bloed afname (één prik, 8 buisjes, 36 milliliter)

= MRI-hersenen

= PET/CT scan

= subcutane injectie (lanreotide / Somatuline AutoSolution óf placebo)

Boertien TM, *et al. BMJ Open* 2020; 10:e038250. doi: 10.1136/bmjopen-2020-038250

BIJLAGE C: BIJWERKINGEN LANREOTIDE – SOMATULINE AUTOSOLUTION

Zoals alle geneesmiddelen kan Somatuline AutoSolution, de merknaam van lanreotide, bijwerkingen veroorzaken. Lang niet iedereen krijgt hier last van.

De meest voorkomende bijwerkingen zijn zoals eerder genoemd maag-darmklachten, galblaasproblemen en reacties op de injectieplaats. Hieronder staat een lijst van de bijwerkingen die het vaakst optreden met Somatuline AutoSolution:

Zeer vaak: komen voor bij meer dan 1 op de 10 gebruikers (>10%)

- Diarree of dunne ontlasting, buikpijn
- Vorming van galstenen; dit veroorzaakt meestal geen klachten. Soms kan een galsteen echter leiden tot een verstopping van de galwegen. Dit kan de volgende klachten veroorzaken: ernstige en plotselinge buikpijn (vaak rechtsboven in de buik), hoge koorts, koude rillingen, geelzucht (het geel worden van de huid en het oogwit), verlies van eetlust, een jeukende huid

Vaak: komen voor bij maximaal 1 op de 10 gebruikers (1-10%)

- Reacties op de injectieplaats zoals zwelling, pijn en jeuk
- Misselijkheid, braken, constipatie (verstopping), winderigheid, opgezette buik, vreemd of opgeblazen gevoel in de buik, dyspepsie (gestoorde spijsvertering/maagklachten), vettige ontlasting
- Verminderde eetlust
- Gewichtsverlies
- Duizeligheid, hoofdpijn
- Pijn in de spieren, gewrichtsbanden, pezen of botten
- Vermoeidheid, gebrek aan energie of slaperigheid
- Trage hartslag
- Haaruitval of minder lichaamsbeharing
- Verwijding van de galwegen
- Verlaagde bloedsuikerspiegel: dit kan klachten geven van overmatig zweten, trillen, hongergevoel en verwardheid
- Verhoogde bloedsuikerspiegel: dit kan klachten geven van dorst, droge mond en vermoeidheid
- Afwijkende lever- en alveesklierwaarden, zoals blijkt uit bloedtesten

Soms: komen voor bij maximaal 1 op de 100 gebruikers (<1%)

- Opvliegers
- Slaapproblemen
- Verandering van de kleur van de ontlasting
- Veranderingen in de concentratie natrium en alkalinefosfaat, zoals blijkt uit bloedtesten

Wanneer u een ernstige bijwerking vermoedt of als u een andere ernstige klacht krijgt die hier niet tussen staat, raadpleeg dan de arts-onderzoeker! Indien geen gehoor of buiten kantooruren, raadpleeg dan de dienstdoende internist(-endocrinoloog) van uw ziekenhuis - *zie* ***bijlage A*** *voor contactgegevens*

Boertien TM, *et al. BMJ Open* 2020; 10:e038250. doi: 10.1136/bmjopen-2020-038250

BIJLAGE D: EXTRA INFORMATIE OVER PET/CT

Algemene informatie
De PET/CT scan wordt in het AMC uitgevoerd op de afdeling Nucleaire Geneeskunde. Deze vindt óf in de middag van het eerste bezoek plaats óf op een apart, tweede bezoek. U hoeft voor de scan niet nuchter te zijn. Op de afdeling Nucleaire Geneeskunde krijgt u een infuusnaald geplaatst in één arm. Via dit infuus krijgt u een kleine hoeveelheid van de licht radioactieve stof toegediend (de radionuclide Gallium-68 DOTATATE). Hierna wordt het infuus verwijderd. Het duurt 45 tot 60 minuten voordat deze stof goed is opgenomen door het lichaam en de hypofyse. Gedurende deze wachttijd mag u in de wachtkamer plaatsnemen, of iets gaan eten of drinken. Hierna neemt u rustig plaats op het bed van de PET/CT-scanner. Het maken van de scan duurt ongeveer 10 minuten, waarbij u op het bed door de brede ring van de scanner wordt geschoven. Deze ring is aan de voor en achterkant open. De laborant is constant aanwezig, en u kunt via de intercom contact met elkaar houden. Belangrijk is dat u tijdens het gehele onderzoek zo stil mogelijk blijft liggen. Na het maken van de scan mag u meteen naar huis. Het toedienen van de radionuclide heeft geen risico voor de omgeving. U mag na de scan meteen alles doen wat u normaal ook zou doen.

Stralingsbelasting
De stralingsbelasting wordt uitgedrukt in mSv (millisievert). De geschatte stralingsbelasting in dit onderzoek is 3.1 mSv. Ter vergelijking: de achtergrondstraling in Nederland is 2-2,5 mSv per jaar. Als u vaker meedoet aan wetenschappelijk onderzoek met stralingsbelasting, moet u er op letten dat de totale hoeveelheid over 5 jaar niet meer is dan 10 mSv. Ook kunt u beter niet mee doen aan dit onderzoek als u in uw beroep meer straling ontvangt dan de achtergrondstraling.

Wat is het risico van straling?
Het lichaam bestaat uit verschillende cellen. Wij hebben bijvoorbeeld hersencellen, spiercellen, bloedcellen, etc. Het zijn de genen (het erfelijke materiaal) in de cel die bepalen hoe de cel functioneert. Röntgenstraling of straling van radioactieve stoffen kan genen beschadigen. Deze beschadiging kan leiden tot kanker of een aangeboren afwijking.

Hoe groot zijn de kansen op deze ernstige gevolgen?
Een blootstelling aan 1 mSv straling geeft een kans van 1 op 17.000 op kanker. Bij een stralingsbelasting van 5 mSv is deze kans 1 op 3.400 en bij 10 mSv 1 op 1.700. Kanker wordt meestal pas 20 jaar of nog later ná de blootstelling aan de straling vastgesteld. De genoemde kansen moet u vergelijken met de kans op kanker van 25% (1 op 4) die elk mens heeft.
Als genen in zaadcellen of eicellen van de ouder beschadigd raken, bestaat het risico op een aangeboren afwijking van het kind. Een dosis van 1 mSv straling zal bij 1 op de 77.000 baby's (van een ouder die deze stralingsdosis kreeg) de oorzaak zijn van een ernstige aangeboren afwijking. Deze kans moet u vergelijken met de kans van 2,3% (23 op de 1000) op een aangeboren afwijking die elke baby heeft.

Boertien TM, *et al. BMJ Open* 2020; 10:e038250. doi: 10.1136/bmjopen-2020-038250

BIJLAGE E: VERZEKERINGSTEKST

Overeenkomstig de Wet medisch-wetenschappelijk onderzoek met mensen heeft de AMC Medical Research B.V. voor medisch-wetenschappelijk onderzoek een verzekering afgesloten die door het onderzoek veroorzaakte schade door dood of letsel van de proefpersoon dekt. Dit betreft zowel schade die zich tijdens de duur van het onderzoek openbaart als schade die zich openbaart, en gemeld is, binnen vier jaar na beëindiging van de deelname aan het onderzoek.
De verzekering biedt een dekking van €450.000 per proefpersoon met een maximum van €3.500.000 voor het hele onderzoek en een maximum van €5.000.000 per jaar voor alle onderzoeken van dezelfde opdrachtgever.

<u>De verzekering biedt dekking</u>:

- voor schade ten gevolge van de verwezenlijking van de deelname aan het wetenschappelijk onderzoek verbonden risico's waarover men niet schriftelijk is ingelicht;
- voor schade ten gevolge van de verwezenlijking van de risico's waarover de deelnemer wel is ingelicht, maar die zich in ernstiger mate voordoet dan is voorzien;
- voor schade ten gevolge van de verwezenlijking van de risico's waarover de deelnemer wel is ingelicht, maar die zeer onwaarschijnlijk werd geacht.

<u>De verzekering biedt geen dekking</u>:

- voor schade die het gevolg is van het uitblijven van een vermindering van de gezondheidsproblemen van de proefpersoon, dan wel het gevolg is van de verdere verslechtering van de gezondheidsproblemen, indien de deelname aan het wetenschappelijk onderzoek plaatsvindt in het kader van de behandeling van deze gezondheidsproblemen;
- voor schade door aantasting van de gezondheid van de proefpersoon waarvan aannemelijk is dat deze zich ook zou hebben geopenbaard wanneer de proefpersoon niet aan het onderzoek had deelgenomen;
- voor schade ten gevolge van deelname aan medisch-wetenschappelijk onderzoek waarbij in de kring van beroepsgenoten gebruikelijke handelingen op het gebied van de geneeskunst met elkaar worden vergeleken en aannemelijk is dat de schade het gevolg is van de toegepaste handelingen;
- voor schade die zich bij een nakomeling van de proefpersoon openbaart als gevolg van een nadelige inwerking van het onderzoek op de proefpersoon of de nakomeling;
- voor schade die het gevolg is van het niet of niet volledig opvolgen van aanwijzingen en instructies door de proefpersoon, indien de proefpersoon daartoe althans in staat is.

De verzekering dekt uitsluitend de schade van natuurlijke personen. De dekking van specifieke schades en kosten is tot bepaalde bedragen beperkt. Om aanspraak te kunnen maken op schadevergoeding dient de proefpersoon in geval van vermeende schade als gevolg van het onderzoek dit te melden aan:

Naam verzekeraar:	Centramed B.A.
Postadres:	Postbus 7374<br>2701 AJ Zoetermeer
Telefoonnummer:	070 301 7070
E-mail:	info@centramed.nl
Polisnummer:	620.872.806

Hiernaast wordt de proefpersoon verzocht contact op te nemen met de hoofdonderzoeker van het onderzoek, prof. dr. E. Fliers (Tel. 020 566 6071).

Boertien TM, et al. BMJ Open 2020; 10:e038250. doi: 10.1136/bmjopen-2020-038250

BIJLAGE F: TOESTEMMINGSFORMULIER

GALANT: Het effect van lanreotide op de grootte van niet-functionerende hypofyse macroadenomen (NFMA)

ABR: NL52821.018.15 – protocol versie 3.0, oktober 2016

Officiële titel: Een gerandomiseerde, placebo-gecontroleerde studie onder patiënten met **Ga**llium-68 DOTATATE PET/CT positieve, klinisch niet-functionerende hypofyse macroadenomen (NFMA) naar het effect van **Lan**reotide autosolution op **T**umorgrootte (**GALANT**)

Ik heb de informatiebrief voor de proefpersoon gelezen. Ik kon aanvullende vragen stellen. Mijn vragen zijn genoeg beantwoord. Ik had genoeg tijd om te beslissen of ik meedoe.

Ik weet dat meedoen helemaal vrijwillig is. Ik weet dat ik op ieder moment kan beslissen om toch niet mee te doen. Daarvoor hoef ik geen reden te geven.

Ik weet dat onverwachte afwijkingen die tijdens het onderzoek naar voren komen altijd met mij besproken zullen worden.

Ik geef toestemming om mijn huisarts en behandelend internist-endocrinoloog te informeren dat ik meedoe aan dit onderzoek.

Ik weet dat mijn persoonsgegevens worden geregistreerd bij Eurocept Homecare als de injecties thuis plaatsvinden.

Ik weet dat mijn contactgegevens in een studiedossier in het AMC worden opgeslagen zodat de arts-onderzoeker met mij contact kan onderhouden.

Ik weet dat het onderzoeksteam, de controleur van het AMC en vertegenwoordigers van de Inspectie voor de Gezondheidszorg mijn onderzoeksgegevens zonder code kunnen inzien.

Ik geef toestemming om mijn gegevens te verzamelen en te gebruiken voor de doelen die in de informatiebrief staan.

Ik geef toestemming om mijn onderzoeksgegevens 15 jaar na afloop van dit onderzoek te bewaren.

Ik geef toestemming om het afgenomen bloed gedurende het onderzoek te bewaren tot publicatie van de resultaten (korter dan 5 jaar), waarna het wordt vernietigd.

Ik geef □ **wel**
□ **geen**
toestemming om in de toekomst benaderd te worden voor een vervolgonderzoek.

Ik wil meedoen aan dit onderzoek.

Naam proefpersoon: ……………………………………………………………………………

Handtekening: ……………………………………………………….… Datum: ………/…..……/……….

Ik verklaar hierbij dat ik deze proefpersoon volledig heb geïnformeerd over het genoemde onderzoek.

Als er tijdens het onderzoek informatie bekend wordt die de toestemming van de proefpersoon zou kunnen beïnvloeden, dan breng ik hem/haar daarvan tijdig op de hoogte.

Naam onderzoeker: ……………………………………………………………………………

Handtekening: ……………………………………………………….… Datum: ………/…..……/……….

Aanvullende informatie is gegeven door (indien van toepassing):

Naam: ……………………………………………………………………………………………….

Functie: ……………………………………………………………………………………………

Handtekening: ……………………………………………………….… Datum: ………/…..……/……….

De proefpersoon krijgt een volledige informatiebrief mee, samen met een kopie van het getekende toestemmingsformulier

Boertien TM, *et al. BMJ Open* 2020; 10:e038250. doi: 10.1136/bmjopen-2020-038250
