# Supplementary material for: The GALANT trial: study protocol of a randomised placebo-controlled trial in patients with a 68Ga-DOTATATE PET-positive, clinically non-functioning pituitary macroadenoma on the effect of lanreotide on tumour size
Source: BMJ Open. 2020 Aug 13;10(8):e038250. doi: 10.1136/bmjopen-2020-038250 (PMC7430490; doi:10.1136/bmjopen-2020-038250)
Supplement: Supplementary data [file bmjopen-2020-038250supp003.pdf]

GALANT study
NL52821.018.15

SUBJECT INFORMATION FOR PARTICIPATION IN MEDICAL RESEARCH

GALANT: The effect of lanreotide on non-functioning pituitary macroadenoma (NFMA) size

Official study title: A randomized placebo-controlled study in patients with a **Ga**llium-68 DOTATATE PET/CT positive, clinically non-functioning pituitary macroadenoma (NFMA) of the effect of **Lan**reotide autosolution on **T**umor (adenoma) size (**GALANT**)

Dear Sir/Madam,

You are kindly requested to take part in a medical-scientific study. We have contacted you because you receive treatment for an adenoma (tumor) of the pituitary gland at the department of Endocrinology of the Academic Medical Center (AMC). Participation is voluntary. Participation requires your written consent. Before you decide whether you want to participate in this study, you will be given an explanation about what the study involves. Please read this information carefully and discuss it with your partner, friends and/or family. General information about participating in a study can be found in the enclosed general brochure on medical research. If you have any questions you can contact the (coordinating) investigator or the independent expert for an explanation. Contact details can be found in **appendix A**, page 7.

1. What is the purpose of the study?

The purpose of this study is to investigate the effect of the medicine lanreotide in the treatment of certain pituitary adenomas. The pituitary gland, or hypophysis, is a small but important endocrine organ. It sits at the base of the brain and is about the size and shape of a bean. It has a central role in the regulation of various hormones. Pituitary adenomas are benign tumors within the pituitary gland. Some adenomas produce excess hormone. If this is not the case the adenoma is called “non-functioning”. If the size exceeds 1 centimeter, it is called a macroadenoma. The growing adenoma can cause pressure on the pituitary gland and disrupt its function. This requires treatment to replace the missing hormones (“substitution therapy”). The growing adenoma can also cause pressure on the optic nerve, which leads to vision disturbances. If this happens, a surgical operation is necessary. Sadly, however, the adenoma grows back in most cases. At this time, no medicinal treatment is available. The departments of Endocrinology of the AMC and the VU Medical Center (VUmc) in Amsterdam have therefore started a study to examine the effect of the medicine lanreotide on the size of non-functioning pituitary macroadenoma. We hope that this medicine can slow the growth of the adenoma or even decrease its size. This could prevent or postpone surgery. The study is realized with the financial support of Ipsen, the pharmaceutical company that produces lanreotide.

2. What exactly is lanreotide?

Lanreotide is a medicinal protein that is similar to the naturally occurring hormone somatostatin. This hormone blocks the release of several other hormones such as growth hormone. The brand name of lanreotide in the Netherlands is Somatuline AutoSolution. Both names are used for the same drug. It is administered through an injection. Lanreotide is already used in the treatment of pituitary adenomas that produce growth hormone or thyroid stimulating hormone. Earlier research has shown that about one-third of non-functioning adenomas might also be susceptible to lanreotide and could benefit from treatment.

Boertien TM, *et al. BMJ Open* 2020; 10:e038250. doi: 10.1136/bmjopen-2020-038250

However, these studies were small and lacked comparison with a control group. Therefore the results are not reliable enough.

3. What does participation involve?

Also see the study overview in ***appendix B***

Study design

This is a randomized, double-blind and placebo-controlled study. Randomization means that chance decides which patient gets which study treatment. There are two treatment groups. One group will receive the medicine lanreotide and the other group will receive a placebo or "sham treatment", in this case a harmless saline solution. Both treatments are administered as an injection. Every patient will also receive their regular care. Double-blind means that neither the patient nor the investigators know which patient is getting which treatment. First, a screening will take place to see if you can participate in the randomization and treatment.

Screening: the first visits

If you are interested in participating in the study you will be invited for the first visit at the AMC. We will first evaluate whether you may participate. The investigator will register some general information about your medical history, medication use, allergies etc. We will also request you to fill in a questionnaire about your health and daily activities. Furthermore the first visit will involve a short physical examination and blood tests (8 tubes, 36 milliliters). The MRI-scan of the brain will be repeated if it is necessary to measure the size of the adenoma again. Only adenomas larger than 1 centimeter can be examined in this study. Apart from the questionnaire, the tests are part of your regular care.

If the first screening is okay, a second screening will follow. This screening consists of a specific PET/CT scan. This scan shows if the adenoma has certain characteristics that could make it susceptible for lanreotide. If so, treatment with lanreotide might be effective. Only in that case participation in the study is possible. The PET/CT takes place at the AMC. If possible on the same day of the first screening, but a second visit could be necessary. Before the scan is performed, you will receive a small amount of a slightly radioactive marker (called a radionuclide) through an intravenous injection. You can find more information about this scan in **appendix D**.

The treatment, further visits and duration of the study

Randomization takes place when the screening shows that participation is possible. Then the treatment with the injections starts. Every 4 weeks (28 days) you will receive one injection, administered in the skin of the buttock ('subcutaneously'). In total you will receive 18 injections of either lanreotide or placebo. A specialized endocrine nurse will administer the injections at the AMC. If travelling to the AMC every 4 weeks is inconvenient for you, the injections can also be administered at home or even at work. Somacare nurses from Eurocept Homecare will perform this. They will register you as a participant in this study so they can contact you about the administration.

Every six months there will be a control visit to the investigator at the AMC, three times during the study. At these visits we will repeat the physical examination, blood tests and the questionnaire. We will also ask about possible side effects or other problems. The first and second control visit will take place on the day of a planned injection, so these can take place at the same time. The third and final control visit will take place after the last injection. Around the first and the last visit the MRI scan of the brain will be repeated to check the size of the adenoma. The duration of the study is about 18 months. In total there will be a minimum of

Boertien TM, *et al. BMJ Open* 2020; 10:e038250. doi: 10.1136/bmjopen-2020-038250

4 and a maximum of 21 visits to the hospital, depending on where the injections are administered. In **appendix B** a useful overview of the study is shown.

4. What is expected of you?

If you decide to participate in the study we will ask you to come to the hospital more often than usual. The amount of visits depends on where the injections are administered. If the injections are administered at the AMC these visits will be short. In order to carry out the study properly it is important to keep to the planned appointments in the hospital and at home. If you need to reschedule you can contact the investigator or Eurocept Homecare. Participation will have no influence on your daily activities. There are no restrictions on eating or drinking. Lanreotide can have some effect on other medicines. That is why it is important to check your medication use before the start of the study. If participation is possible you can continue all of your usual medication. We will discuss it with you if extra monitoring is necessary. It is important to contact the investigator before starting to use any new medicines during the study.

For female participants

If you are breastfeeding, pregnant, or planning to become pregnant, you cannot participate in this study. For fertile women it is important to use adequate contraception. This is because there is still not enough data on the effects of lanreotide on the unborn child. If you do, however, become pregnant during the study, please contact the investigator. The pregnancy will then be monitored more closely.

5. Will participation affect your regular treatment?

Participation will not affect your regular treatment of the pituitary adenoma. Regular treatment consists of monitoring complaints, regular blood tests and checking the size of the adenoma; next to the substitution of missing hormones. This treatment is continued during the study. Certain complaints can indicate increasing size of the adenoma. If this occurs during the study, you will undergo an extra MRI-scan. If the scan shows that surgery is needed to remove the adenoma, participation in the study will stop. Withdrawal from the study will not affect your regular treatment.

6. Can you expect any side effects or discomfort?

Lanreotide

Like all medicines lanreotide may have side effects. The most common side effects are listed below. These occur in about one in ten people (10%):

- Gastrointestinal complaints such as diarrhea, abdominal pain or nausea (especially after the first injection)
- Formation of gallstones (mostly without any symptoms)
- Skin reactions where the injection is given such as swelling, pain or itching

Other known side effects are less common or rare. The drug may also have side effects that are still unknown. Please see **appendix C** for a more extensive list of possible side effects and information on who to contact if any side effect becomes severe.

MRI scans (regular care)

An MRI scan is performed using a magnet, this doesn’t involve any exposure to radiation. Because of the powerful magnet an MRI scan cannot be performed if there are small parts of metal in the body, such as

Boertien TM, *et al. BMJ Open* 2020; 10:e038250. doi: 10.1136/bmjopen-2020-038250

metal fragments in the eye or certain implants. The magnet can also interfere with implanted electronic devices such as a pacemaker.

To perform the MRI scan a contrast agent is administered through an intravenous injection. During the injection you may shortly experience harmless side effects like headache and nausea. If you had an allergic reaction during earlier MRI scans you cannot participate in this study.

PET/CT scan and the radionuclide
The earlier mentioned radionuclide used to perform the PET/CT is called "Gallium-68 DOTATATE". (Allergic) reactions to this radionuclide are rare. The injection to administer the radionuclide can be bothersome and may cause some bruising.

The radionuclide is slightly radioactive. The marker stays radioactive just long enough to perform the scan and generate the images. During this time you will be exposed to a small amount of radiation, estimated 3.1 mSv (millisievert). More extensive information on radiation exposure can be found in **appendix D**.

7. What are the possible advantages and disadvantages of participation?

The results of earlier research suggest that lanreotide may reduce the growth of non-functioning pituitary adenoma, but this is not certain. We can therefore not predict whether you will benefit from participation in this study. The current study will examine the effect of lanreotide more accurately and compare it with a control group. The chance to be part of the control group and receive placebo injections is 50% (one in two). This can be a disadvantage, also because of the frequent appointments at home or at the hospital to receive the injections. Further disadvantages are the additional PET/CT scan with the exposure to radiation and the possible discomfort and side effects of the injections. Blood tests are performed 4 times during the study period (each time 8 tubes and 36 milliliters). This is part of your regular care. If the blood tests or the scans lead to incidental findings about your health we will discuss these with you. If you do not want to be informed about these findings you cannot participate in the study.

8. What if you do not wish to participate or wish to stop participating?

It is up to you to decide whether or not to participate in the study. Participation is voluntary. If you do not want to participate, you will continue your usual treatment as before.
If you do participate in the study, you can always change your mind. You may stop participation at any time during the study. This is not harmful and will not affect your usual treatment. You do not have to say why you are stopping, but you do need to tell the investigator immediately. Participation may also be stopped by the investigator if it is considered best for you.

If there is any new information about the study that is important for you, the investigator will let you know. You will then be asked whether you still want to continue your participation. The data collected until that time can still be used for the study. Additional information about participation and your rights can be found in the general brochure.

9. How will your data be handled?

Privacy
For this study your medical and personal data will be collected and used in several ways. All your data will remain confidential.

Here we list where and why your personal data will be collected:

- At Eurocept Homecare your personal data will be registered in order to plan and administer injections at home.
- At the AMC the coordinating investigator will store your contact information so that she can reach you if necessary.

Data for the study will be collected and stored using a study code. Each participant will get a study code that replaces all personal data. Only the research team knows the code given to your data. In communication and reports about the study the study code will be used to protect your privacy.

Access to your data
To check whether the study is being performed according to the guidelines, some people may access the study data without using the code. Apart from the research team this concerns the monitor of the study and the Healthcare Inspectorate. They are obliged to keep your data secret. If representatives of Ipsen, the financier of the study and manufacturer of lanreotide, need to check the study, they only have access to the encoded data.

Storing of data
All data collected during this study needs to be stored for 15 years. If you sign the consent form, you consent to your medical and personal data being collected, stored and accessed. General information about the usage and storage of data can be found in the general brochure on medical research.

Storing of blood
During the study period blood tests are performed as part of your regular care. Part of the collected blood will be stored for a longer period than usual, also using the study code. We will store the blood in case we need to perform additional tests. The stored blood samples will only be used for this study. It will be destroyed once the study is concluded. The study is concluded once all the participants have completed the study and the results can be published. This will not be longer than 5 years. If you sign the consent form, you consent to the temporary storage of your blood samples.

10. Are you insured if you participate in this study?

Insurance has been taken out for everyone participating in this study. This insurance covers damage caused by the study. This applies to damage manifesting during the study or within four years of the end of the study. The insurance does not cover all damages. **Appendix E** contains more information about the insurance and the contact information.

11. Will my GP and/or my treating specialist be informed if I participate?

We will send your GP ("huisarts") and your treating internist-endocrinologist a letter to let them know that you are participating in the study. This is for your own safety. If you do not agree to this, you cannot participate in this study.

12. Is there a compensation when you participate?

The study medication, additional visits and PET/CT scan for the study are free of charge for you. You will not be paid for your participation in this study, but you will be reimbursed for your travel costs.

Boertien TM, *et al. BMJ Open* 2020; 10:e038250. doi: 10.1136/bmjopen-2020-038250

13. Which Ethics Committee has approved this study?
The Ethics Committee of the AMC has approved this study. General information about the assessment of research can be found in the general brochure on medical research.

14. Any questions?
You do not have to decide right away whether you want to participate in the study. In about a week we will get in touch with you to inform about your decision. If you have any questions or would like more information, please contact the coordinating investigator, Tessel Boertien. If you would like independent advice about participation in this study, you may contact the independent doctor M.R. Soeters, internist-endocrinologist. He knows about the study but is not involved in it. All contact details can be found in **appendix A**.

Thank you for your attention and kind regards,

T.M Boertien, MD, coordinating investigator and project leader
Prof. E. Fliers, MD PhD, internist-endocrinologist and principal investigator AMC

Appendices:
- Separate: Brochure 'Medical research. General information for research participants'
- Appendix A: Contact details and hospital information
- Appendix B: Study overview
- Appendix C: Side effects of lanreotide – Somatuline AutoSolution
- Appendix D: Additional information on PET/CT and radiation exposure
- Appendix E: Insurance information
- Appendix F: Informed Consent form

APPENDIX A: INFORMATION ON THE PARTICIPATING CENTERS & CONTACT DETAILS

Coordinating investigator & project leader

T.M. Boertien, MD
PhD candidate Endocrinology AMC & VUmc
email: **t.m.boertien@amsterdamumc.nl**
Tel. **020 566 3507** / 020 566 6071 (secr.)

Study postal address: Academisch Medisch Centrum, t.a.v. T.M. Boertien
Afdeling Endocrinologie & Metabolisme, K2-283
Meibergdreef 9, 1105 AZ Amsterdam

Independent expert

M.R. Soeters, MD PhD, internist-endocrinologist
email: m.r.soeters@amsterdamumc.nl
Tel. 020 566 6071

Information for Academic Medical Center (AMC)

Address: Meibergdreef 9
1105 AZ Amsterdam
General no.: 020 566 9111
Nuclear medicine: 020 566 2775 (7:30-17:00)

In case of acute problems outside office hours you can call the general number of the AMC (020 566 9111) 24 hours a day and ask to be put through to the attending internist-endocrinologist. Always mention your participation in this study.

Principal investigator

Prof. E. Fliers, MD PhD, internist-endocrinologist
email: e.fliers@amsterdamumc.nl
Tel. 020 566 6071

Endocrine nurses

Moniek Gent-Houben and Shariefa Peters-Amanatkhan
e-mail fk.endocrinologie@amsterdamumc.nl
Tel. 020 566 2990

AMC department for patient information and complaints

The AMC department for patient information and complaints is at your service for general questions and information about matters such as participating in medical-scientific research. If you are not satisfied or unhappy with the care, treatment or services, you can inform the staff about your complaint. They can inform you about the procedure of filing a complaint and if necessary call in the complaints officer.

Location within AMC: outpatients clinic at the ground floor, A0-144

Opening hours:	Monday to Friday from 9:00 to 12:30 hrs
email:	patientenvoorlichting@amc.nl
Tel.:	020 566 3355
Postal address:	Academisch Medisch Centrum, t.a.v. patiëntenvoorlichting, A0-144
	Postbus 22660, 1100 DD Amsterdam

Contact details Eurocept Homecare

Tel.:	035 528 8375 (24 hrs a day)
email	galantstudie@eurocept.nl

Boertien TM, *et al. BMJ Open* 2020; 10:e038250. doi: 10.1136/bmjopen-2020-038250

GALANT study
NL52821.018.15

APPENDIX B: STUDY OVERVIEW

This is an overview of the study period. The study visit with **bold** numbers will definitely take place. In this example the PET/CT is planned during a separate visit (no. 2). It could be that this scan can already take place during visit no. 1. The 7th and the 13th injection will take place on a day that you also have a study visit at the AMC. The total amount of study visits depends on where the rest of the injections are administered: at home or at the AMC. That is why those visit numbers are between parentheses ( ). If all injections take place at the AMC there will be 21 visits in total.

RANDOMIZATION ↓

Time point → Study visit no. →	start **1**	**2**	(3 – 8)	6 mo. **3** (9)	(10 – 14)	12 mo. **4** (15)	(16 – 20)	18 mo. **5** (21)	END OF STUDY
Location →	AMC	AMC	AMC/home	AMC	AMC/home	AMC	AMC/home	AMC	
Duration in minutes →	90	90	15	90	15	60	15	75	

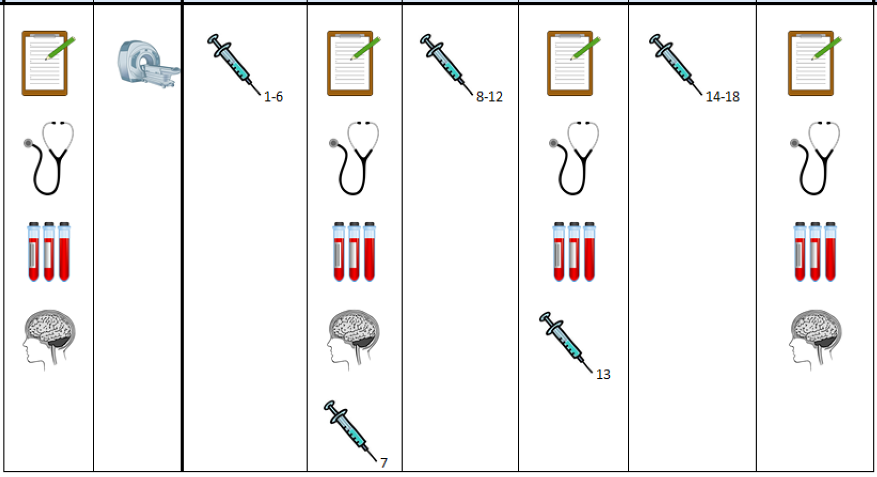

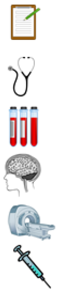

= interview and quality of life questionnaire
= short physical examination (blood pressure, heart rate, height, weight; if necessary more extensive)
= blood withdrawal (single puncture, 8 tubes, 36 milliliters)
= MRI scan of the brain
= PET/CT scan
= subcutaneous injection (lanreotide / Somatuline AutoSolution OR placebo)

Boertien TM, *et al. BMJ Open* 2020; 10:e038250. doi: 10.1136/bmjopen-2020-038250

APPENDIX C: SIDE EFFECTS OF LANREOTIDE – SOMATULINE AUTOSOLUTION

Like all medicines, Somatuline AutoSolution (the brand name of lanreotide) can cause side effects, although they do not affect everyone.

As mentioned earlier, the most commonly reported side effects are gastrointestinal complaints, gallbladder problems and injection site reactions. The list below shows the side effects that can occur with Somatuline AutoSolution according to their frequencies:

Very common: may affect more than 1 in 10 people (>10%):

- Diarrhea, loose stools, abdominal pain
- Formation of gallstones; this normally doesn't cause symptoms. In some cases however a gallstone can lead to blockage of a bile duct. This can cause the following symptoms: severe and sudden abdominal pain, high fever, chills, jaundice (yellowing of the skin and whites of the eyes), loss of appetite, itchy skin

Common: may affect up to 1 in 10 people (1-10%):

- Site reactions where the injection is given such as swelling, pain or itchiness
- Nausea, vomiting, constipation, gas formation/wind, stomach bloating or discomfort, indigestion, excess fat in stools
- Decrease in appetite
- Weight loss
- Feeling dizzy, headaches
- Pain that affects muscles, ligaments, tendons and bones
- Lack of energy or feeling tired
- Slow heart beat (sinus bradycardia)
- Loss of hair or less development of body hair
- Enlargement of the bile ducts between your liver and gallbladder and the intestine
- Lowered blood sugar level: this can cause symptoms of excessive sweating, feeling shaky, feeling hungry and confusion
- Heightened blood sugar level: this can cause symptoms of thirst, a dry mouth and feeling more tired than usual
- Abnormal liver and pancreas test results, shown in blood tests

Uncommon: may affect up to 1 in 100 people (<1%):

- Hot flushes
- Difficulty sleeping
- A change in the color of the stools
- Changes to sodium and alkaline phosphatase levels, shown in blood tests

If any of these side effects becomes severe or if you experience a severe symptom that is not listed here, please contact the coordinating investigator or, if there is no answer or outside office hours, the attending internist-endocrinologist of your hospital - *see **appendix A** for contact details*

Boertien TM, et al. BMJ Open 2020; 10:e038250. doi: 10.1136/bmjopen-2020-038250

APPENDIX D: ADDITIONAL INFORMATION ON PET/CT & RADIATION EXPOSURE

General information

The PET/CT scan will be performed at the department of Nuclear Medicine of the AMC. This will either take place in the afternoon of the first visit or during a separate, second visit. You are allowed to eat and drink before the scan. At the department of Nuclear Medicine, a small intravenous line will be placed in your arm. A small amount of the slightly radioactive marker (the radionuclide Gallium-68 DOTATATE) will be administered through the line. Following the injection the line will be removed. It takes 45 to 60 minutes for the radionuclide to be distributed through the body. During this time you can take place in the waiting room or go and have something to eat or drink. Then you will be placed comfortably on the PET/CT table and scanned. During the scan the table is moving through a large ring that is open on both sides. The technician is present continuously and you can contact him or her through the intercom. The imaging session will take about 10 minutes. It is important that you remain as still as possible. After the scan you may go home directly and continue your usual activities. The marker loses its radioactivity very quickly; it carries no risks for your environment.

Radiation exposure

The PET/CT involves using radioactive markers. Radiation exposure is expressed in mSv (millisievert). The total amount of radiation you will be exposed to in this study is estimated at 3.1 mSv. To compare: the background radiation in the Netherlands is 2-2.5 mSv per year. If you participate in scientific research involving exposure to radiation more often, you should discuss with the investigator whether participation now would be safe. The total amount of radiation due to scientific research should not exceed 10 mSv in 5 years. Participation is advised against if you are exposed to an amount of radiation that exceeds the background radiation as part of your job.

What is the risk of radiation?

The body is made up of different cells. There are brain cells, muscle cells, blood cells etc. The genes inside these cells carry the genetic material and determine the function of the cells. X-rays or radiation from radioactive substances can damage these genes. This can lead to cancer or a congenital (inborn) disorder.

What are the chances of these severe consequences?

Cancer due to radiation usually is not detected until 20 years after the exposure. An exposure of 1 mSv generates a chance of 1 in 17,000 to develop cancer. With an exposure of 5 mSv this chance is increased to 1 in 3,400 and with an exposure of 10 mSv to 1 in 1,700. You need to compare these chances with the chance of 25% (1 in 4) to develop cancer that every human has.

If genes in the reproductive cells (sperm and eggs) are damaged, there is a risk of a congenital (inborn) disorder of the child. An exposure of 1 mSv radiation of the parent will be the cause of a severe congenital disorder in 1 in 77,000 babies. You need to compare this chance with the chance of 2,3% (23 in 1,000) of a congenital disorder that every baby has.

APPENDIX E: INSURANCE INFORMATION

Insurance has been taken out by AMC Medical Research B.V. for everyone participating in this study. The insurance covers damage due to participation in the study. This applies to damage manifesting during the study or within four years of the end of the study. The insurance offers a cover of € 450,000 per study subject, with a maximum of € 3,500,000 for the entire study and € 5,000,000 annually for all studies from the same sponsor. You must notify the insurance company about the damage within those four years. This is set out in the Medical Research (Human Subjects) Compulsory Insurance Decree. This decree is listed on the website of the Central Committee on Research Involving Human Subjects www.ccmo.nl.

The insurance policy covers the following damage:

- damage as a result of a risk of participation in the clinical trial that you were not informed about in the written information;
- damage as a result of a risk that you were informed about in the written information, but which proved more serious than foreseen;
- damage as a result of a risk that you were informed about in the written information, but which was deemed very unlikely to occur.

The insurance policy does **not** cover the following damage:

- damage due to your health problems not being alleviated or your health problems deteriorating further, in case participation in the clinical trial took place within the context of treatment of those health problems;
- damage to your health that would also have occurred if you had not participated in the study;
- damage as a result of an existing treatment method for research into existing methods of treatment;
- damage to descendants as a result of a negative effect of the study on you or your descendants;
- damage resulting from not or not entirely following directions or instructions.

The insurance shall cover losses suffered by natural persons only. In the event of damage please contact the insurance company directly:

Name company:	Centramed B.A.
Postal address:	Postbus 7374
	2701 AJ Zoetermeer
Telephone number:	070 301 7070
Email:	info@centramed.nl
Policy number:	620.872.806

Furthermore you are requested to contact the principal investigator of the study, prof. E. Fliers (tel. 020 566 6071).

Boertien TM, *et al. BMJ Open* 2020; 10:e038250. doi: 10.1136/bmjopen-2020-038250

APPENDIX F: INFORMED CONSENT FORM

GALANT: The effect of lanreotide on non-functioning pituitary macroadenoma (NFMA) size

ABR: NL52821.018.15 – protocol version 3.0, 13 October 2016

Official study title: A randomized placebo-controlled study in patients with a **Ga**llium-68 DOTATATE PET/CT positive, clinically non-functioning pituitary macroadenoma (NFMA) of the effect of **Lan**reotide autosolution on **T**umor (adenoma) size (**GALANT**)

I have read the subject information letter. I was also able to ask questions. My questions have been answered to my satisfaction. I had enough time to decide whether to participate.

I know that participation is voluntary. I know that I may decide at any time not to participate after all or to withdraw from the study. I do not need to give a reason for this.

I know that incidental findings about my health during this study will be discussed with me.

I give permission to inform my GP and my treating specialist about my participation in this study.

I know that my personal data will be registered at Eurocept Homecare if the injections take place at home.

I know that my contact information will be stored in a study file at the AMC so that the coordinating investigator can contact me about the study.

I know that the research team, the study monitor and representatives of the Healthcare Inspectorate can access my data without the study code.

I consent to the collection and usage of my data for the purposes stated in the information letter.

I consent to my data being stored for another 15 years after this study.

I consent to my blood samples being stored during the study until publication of the results (maximum of 5 years), after which it shall be destroyed.

I ☐ **do**
☐ **do not**
consent to being contacted again after this study for a follow-up study.

Boertien TM, *et al. BMJ Open* 2020; 10:e038250. doi: 10.1136/bmjopen-2020-038250

I want to participate in this study.

Name study subject: …………………………………………………………………………….

Signature: ……………………………………………………………… Date: …………/…………/…………

I hereby declare that I have fully informed this study subject about this study.

If information comes to light during the course of the study that could affect the study subject's consent, I will inform him/her of this in a timely fashion.

Name investigator: ……………………………………………………………………………….

Signature: ……………………………………………………………… Date: …………/…………/…………

Additional information was given by (if applicable):

Name: ……………………………………………………………………………………….

Job title: ………………………………………………………………………………….

Signature: ……………………………………………………………… Date: …………/…………/…………

The study subject receives a complete information letter and a copy of the signed informed consent form.

Boertien TM, *et al. BMJ Open* 2020; 10:e038250. doi: 10.1136/bmjopen-2020-038250
