# Supplementary material for: The GALANT trial: study protocol of a randomised placebo-controlled trial in patients with a 68Ga-DOTATATE PET-positive, clinically non-functioning pituitary macroadenoma on the effect of lanreotide on tumour size
Source: BMJ Open. 2020 Aug 13;10(8):e038250. doi: 10.1136/bmjopen-2020-038250 (PMC7430490; doi:10.1136/bmjopen-2020-038250)
Supplement: Author's manuscript [file bmjopen-2020-038250.draft_revisions.pdf]

BMJ Open is committed to open peer review. As part of this commitment we make the peer review history of every article we publish publicly available.

When an article is published we post the peer reviewers' comments and the authors' responses online. We also post the versions of the paper that were used during peer review. These are the versions that the peer review comments apply to.

The versions of the paper that follow are the versions that were submitted during the peer review process. They are not the versions of record or the final published versions. They should not be cited or distributed as the published version of this manuscript.

BMJ Open is an open access journal and the full, final, typeset and author-corrected version of record of the manuscript is available on our site with no access controls, subscription charges or pay-per-view fees (http://bmjopen.bmj.com).

If you have any questions on BMJ Open's open peer review process please email info.bmjopen@bmj.com

BMJ Open

The GALANT trial: study protocol of a randomised placebo-controlled trial in patients with a ^{68}*Ga*-DOTATATE PET-positive, clinically nonfunctioning pituitary macroadenoma on the effect of *lan*reotide on *t*umour size

Journal:	*BMJ Open*
Manuscript ID	bmjopen-2020-038250
Article Type:	Protocol
Date Submitted by the Author:	04-Mar-2020

Complete List of Authors:	Boertien, Tessel; Amsterdam UMC - Locatie AMC, Department of Endocrinology and Metabolism<br>Drent, Madeleine; Amsterdam UMC - Locatie VUMC, Department of Internal Medicine, Section of Endocrinology<br>Booij, Jan; Amsterdam UMC - Locatie AMC, Department of Radiology and Nuclear Medicine<br>Majoie, Charles; Amsterdam UMC - Locatie AMC, Department of Radiology and Nuclear Medicine<br>Stokkel, Marcel; Netherlands Cancer Institute, Department of Nuclear Medicine<br>Hoogmoed, Jantien; Amsterdam UMC - Locatie AMC, Department of Neurosurgery, Neurosurgical Centre Amsterdam<br>Pereira, Alberto; Leiden University Medical Center, Department of Medicine, Division of Endocrinology, and Centre for Endocrine Tumors Leiden (CETL)<br>Biermasz, Nienke; Leiden University Medical Center, Department of Medicine, Division of Endocrinology, and Centre for Endocrine Tumors Leiden (CETL)<br>Simsek, Suat; Noordwest Ziekenhuisgroep, Department of Internal Medicine; Amsterdam UMC - Locatie VUMC, Department of Internal Medicine, Section of Endocrinology<br>Groote Veldman, Ronald; Medisch Spectrum Twente, Department of Internal Medicine<br>Tanck, Michael; Amsterdam UMC - Locatie AMC, Department of Clinical Epidemiology, Biostatistics and Bioinformatics<br>Fliers, Eric; Amsterdam UMC - Locatie AMC, Department of Endocrinology and Metabolism<br>Bisschop, Peter; Amsterdam UMC - Locatie AMC, Department of Endocrinology and Metabolism

Keywords:	Pituitary disorders < DIABETES & ENDOCRINOLOGY, Endocrine tumours < DIABETES & ENDOCRINOLOGY, Nuclear radiology < RADIOLOGY & IMAGING, Clinical trials < THERAPEUTICS

SCHOLARONE™
Manuscripts

I, the Submitting Author has the right to grant and does grant on behalf of all authors of the Work (as defined in the below author licence), an exclusive licence and/or a non-exclusive licence for contributions from authors who are: i) UK Crown employees; ii) where BMJ has agreed a CC-BY licence shall apply, and/or iii) in accordance with the terms applicable for US Federal Government officers or employees acting as part of their official duties; on a worldwide, perpetual, irrevocable, royalty-free basis to BMJ Publishing Group Ltd ("BMJ") its licensees and where the relevant Journal is co-owned by BMJ to the co-owners of the Journal, to publish the Work in this journal and any other BMJ products and to exploit all rights, as set out in our licence.

The Submitting Author accepts and understands that any supply made under these terms is made by BMJ to the Submitting Author unless you are acting as an employee on behalf of your employer or a postgraduate student of an affiliated institution which is paying any applicable article publishing charge ("APC") for Open Access articles. Where the Submitting Author wishes to make the Work available on an Open Access basis (and intends to pay the relevant APC), the terms of reuse of such Open Access shall be governed by a Creative Commons licence – details of these licences and which Creative Commons licence will apply to this Work are set out in our licence referred to above.

Other than as permitted in any relevant BMJ Author's Self Archiving Policies, I confirm this Work has not been accepted for publication elsewhere, is not being considered for publication elsewhere and does not duplicate material already published. I confirm all authors consent to publication of this Work and authorise the granting of this licence.

For peer review only

The GALANT trial: study protocol of a randomised placebo-controlled trial in patients with a ^{68}*Ga*-DOTATATE PET-positive, clinically nonfunctioning pituitary macroadenoma on the effect of *lan*reotide on *tu*mour size

Tessel M. Boertien[1], Madeleine L. Drent[2], Jan Booij[3], Charles B. L. M. Majoie[3], Marcel P. M. Stokkel[4], Jantien Hoogmoed[5], Alberto M. Pereira[6], Nienke R. Biermasz[6], Suat Simsek[2,7], Ronald Groote Veldman[8], Michael W.T. Tanck[9], Eric Fliers[1]*, Peter H. Bisschop[1]*

[1]*Department of Endocrinology and Metabolism, Amsterdam UMC, location AMC, University of Amsterdam, Meibergdreef 9, Amsterdam, the Netherlands*
[2]*Department of Internal Medicine, Section of Endocrinology, Amsterdam UMC, location VUMC, VU University, De Boelelaan 1117, Amsterdam, the Netherlands*
[3]*Department of Radiology and Nuclear Medicine, Amsterdam UMC, location AMC, University of Amsterdam, Meibergdreef 9, Amsterdam, the Netherlands*
[4]*Department of Nuclear Medicine, Netherlands Cancer Institute, Amsterdam, the Netherlands*
[5]*Department of Neurosurgery, Neurosurgical Centre Amsterdam, Amsterdam UMC, location AMC, University of Amsterdam, Meibergdreef 9, Amsterdam, the Netherlands*
[6]*Department of Medicine, Division of Endocrinology, and Centre for Endocrine Tumors Leiden (CETL), Leiden University Medical Centre, Leiden, the Netherlands*
[7]*Department of Internal Medicine, Northwest Clinics, Alkmaar, the Netherlands*
[8]*Department of Internal Medicine, Medical Spectrum Twente, Enschede, the Netherlands*
[9]*Department of Clinical Epidemiology, Biostatistics and Bioinformatics, Amsterdam UMC, location AMC, University of Meibergdreef 9, Amsterdam, the Netherlands*
**Equal contribution*

Corresponding author: Tessel M. Boertien; t.m.boertien@amsterdamumc.nl
Amsterdam UMC, location AMC
Dept. of Endocrinology & Metabolism, K2-283
Meibergdreef 9
1105 AZ Amsterdam
THE NETHERLANDS

Word count:
Abstract: 265
Main text: 4,179

For peer review only - http://bmjopen.bmj.com/site/about/guidelines.xhtml

Abstract

Introduction At present, there is no approved medical treatment option for patients with nonfunctioning pituitary adenoma. A number of open-label studies suggest that treatment with somatostatin analogues may prevent tumour progression. *In vivo* somatostatin receptor imaging using ^{68}Ga-DOTATATE PET could help in preselecting patients potentially responsive to treatment. Our aim is to investigate the effect of the somatostatin analogue lanreotide as compared to placebo on tumour size in patients with a ^{68}Ga-DOTATATE PET-positive nonfunctioning pituitary macroadenoma (NFMA).

Methods and analysis The GALANT study is a multicentre, randomised, double-blind, placebo-controlled trial in adult patients with a suprasellar extending NFMA. Included patients undergo a ^{68}Ga-DOTATATE PET/CT of the head and tracer uptake is assessed after coregistration with pituitary MRI. Forty-four patients with a ^{68}Ga-DOTATATE PET-positive NFMA are randomised in a 1:1 ratio between lanreotide 120 mg or placebo, both administered as subcutaneous injections every 28 days for 72 weeks. The primary outcome is the change in cranio-caudal tumour diameter on pituitary MRI after treatment. Secondary outcomes are change in tumour volume, time to tumour progression, change in quality of life and number of adverse events. Final results are expected in the second half of 2021.

Ethics and dissemination The study protocol has been approved by the medical research ethics committee of the Academic Medical Centre (AMC) of the Amsterdam University Medical Centres and by the Dutch competent authority. It is an investigator-initiated study with financial support by Ipsen Farmaceutica BV. The AMC, as sponsor, remains owner of all data. Results will be submitted for publication in a peer-reviewed journal.

Trial registration number NL5136 (Netherlands Trial Register); pre-recruitment.

BMJ Open keywords: pituitary disorders, endocrine tumours, nuclear radiology, clinical trials

Strengths and limitations of this study

- The GALANT study is the first double-blind and placebo-controlled intervention trial in nonfunctioning pituitary macroadenoma patients.
- ^{68}Ga-DOTATATE PET/CT is used to select only those patients with a somatostatin receptor-positive adenoma for randomisation between lanreotide and placebo.
- The study is powered on change in cranio-caudal adenoma diameter, a clinically relevant outcome in the prevention of optic chiasm compression.
- The exclusion of patients previously treated with radiotherapy may present a bias in this study towards NFMA with a less aggressive growth rate.

For peer review only

For peer review only - http://bmjopen.bmj.com/site/about/guidelines.xhtml

List of abbreviations

^{111}In-DTPA-octreotide	Indium-111 labeled diethylenetriaminepentaacetic acid-octreotide
^{68}Ga-DOTATATE	Gallium-68 labeled dodecanetetraacetic acid-[Tyr^{3}]-octreotate
AMC	Academic Medical Centre (Amsterdam UMC, University of Amsterdam)
Amsterdam UMC	Amsterdam University Medical Centres
ANCOVA	analysis of covariance
CRF	case report form
CT	computer tomography
ITT	intention-to-treat
LUMC	Leiden University Medical Centre
MAR	missing at random
MBq	megabecquerel
MREC	medical research ethics committee
MRI	magnetic resonance imaging
mSv	millisievert
NFA	nonfunctioning adenoma
NFMA	nonfunctioning macroadenoma
PET	positron emission tomography
ROI	region of interest
SF-36	36-item Short Form Health Survey
SPECT	single-photon emission computed tomography
SPIRIT	Standard Protocol Items: Recommendations for Interventional Trials
(S)AE	(serious) adverse event
SSA	somatostatin analogue(s)
SSTR	somatostatin receptor
SUSAR	suspected unexpected serious adverse reaction
SUV	standard uptake value
VUMC	VU University Medical Center (Amsterdam UMC, VU University)

For peer review only

For peer review only - http://bmjopen.bmj.com/site/about/guidelines.xhtml

INTRODUCTION

Pituitary adenomas are benign tumours of the pituitary gland and are recognised as the third most common intracranial neoplasm.[1] Clinically nonfunctioning adenomas (NFA) account for 15-50% of all pituitary adenomas.[2] Because of the lack of clinical and biochemical signs of hormonal hypersecretion, NFA can go undetected for a long time and usually are macroadenomas, i.e. diameter ≥1cm, at time of diagnosis. Presenting symptoms are related to mass effects and include pituitary insufficiency, headache, and visual disturbances.[2]

Transsphenoidal resection of the adenoma is the main therapeutic approach. However, the overall remission rate after surgery is only 44%, and regrowth of residual tumour occurs in over 50% of patients.[3,4] Consequently, a substantial part of patients requires additional treatment during follow-up. Repeat surgery is recommended in case of persistent or recurrent chiasm compression, but the benefit/risk ratio is less favourable than with primary surgery.[5,6] Adjuvant radiotherapy is effective in controlling tumour (re)growth, but its use is restricted in view of the high risk of hypopituitarism and rare but important complications such as optic nerve damage and secondary brain tumours.[7–9]

Effective medical therapy as alternative option in NFA management would therefore be of great value. The expression of somatostatin receptor (SSTR) subtypes in the majority of NFA provides a potential target for receptor-mediated therapy.[10–16] The somatostatin analogues (SSA) octreotide and lanreotide have highest affinity for $SSTR_2$ and $SSTR_5$, and these subtypes are implicated in antiproliferative effects.[17] Indeed, treatment with octreotide seems to prevent tumour progression in selected NFA patients, although interpretation is hampered by methodological heterogeneity among studies and open-label design.[10,18]

SSA could thus play a role in the management of NFA patients, but outcomes of well-designed placebo-controlled studies are lacking. A promising approach may be *in vivo* SSTR imaging to preselect patients potentially responsive to treatment.[10,19] Thus far, SSTR expression in NFA has been assessed through ^{111}In-DTPA-octreotide scintigraphy or SPECT.[10,14,15,20] However, interpretation of pituitary adenoma uptake using scintigraphy/SPECT is limited by low spatial resolution.[21] The introduction of the PET radiotracer ^{68}Ga-DOTATATE has enabled high-resolution PET/CT imaging with additional superb $SSTR_2$ affinity.[22,23]

The aim of the present study is to investigate the effect of the SSA lanreotide, as compared to placebo, on adenoma size in patients with a ^{68}Ga-DOTATATE PET-positive nonfunctioning pituitary macroadenoma (NFMA).

METHODS AND ANALYSIS

This manuscript was written following the Standard Protocol Items: Recommendations for Interventional Trials (SPIRIT) guideline on reporting of intervention trial protocols (online supplementary material S1).[24,25]

For peer review only - http://bmjopen.bmj.com/site/about/guidelines.xhtml

Study design and setting

The GALANT study is a multicentre, randomised, double-blind, placebo-controlled, parallel-group, superiority trial in patients with a ^{68}Ga-DOTATATE PET-positive NFMA, investigating the effect of lanreotide versus placebo on tumour size. Forty-four patients are randomised in a 1:1 ratio and the primary outcome measure is the absolute change in cranio-caudal adenoma diameter after 72 weeks of treatment (study flowchart in **Figure 1**). The study is conducted in an outpatient setting and eligible patients are referred by endocrinologists at academic and non-academic hospitals in the Netherlands for inclusion at one of the participating centres (Amsterdam University Medical Centres (locations AMC and VUMC) and Leiden University Medical Centre). The study-related ^{68}Ga-DOTATATE PET/CT is performed at either the AMC or the Netherlands Cancer Institute in Amsterdam, an imaging-only participating centre. The study is investigator-initiated with the AMC as sponsor. Financial support is provided by Ipsen Farmaceutica BV.

Eligibility criteria

Adult patients diagnosed with an NFMA with suprasellar extension, either surgery-naive or with a postoperative remnant, are eligible for inclusion. Detailed inclusion and exclusion criteria are listed in **Figure 2**. NFMA diagnosis is based on evidence for a pituitary macroadenoma on dedicated pituitary MRI and absence of clinical and biochemical signs of hormonal hypersecretion. Extension of the NFMA above the sellar diaphragm is a supplemental inclusion criterion since upward growth towards the optic nerves and chiasm is clinically relevant. In case of optic chiasm compression, current visual field defects must be excluded as this presents an indication for resection. Previous therapy with radiation, SSA or dopamine receptor agonists are exclusion criteria as these can influence the primary outcome. Other exclusion criteria are in accordance with the summary of product characteristics of lanreotide (Somatuline AutoSolution®).

Study outline

Eligible patients are approached by their treating endocrinologist and permission is asked to be contacted by a trial staff member (TB). The study is explained in full and written information is provided (online supplementary material S2 and S3). After obtaining written informed consent by a trial staff clinician, patient and NFMA characteristics are recorded (age, gender, medication use, laboratory and MRI results, current pituitary hormone deficiencies, prior NFMA treatment and further relevant medical history). At the screening visit, patients undergo a ^{68}Ga-DOTATATE PET/CT of the head. Only patients with a PET-positive NFMA are randomised for treatment. Before start of treatment, a baseline visit is planned consisting of a fasting blood sample, short physical examination, quality of life questionnaire, structured interview, and pregnancy test, if applicable. Pituitary MRI is only repeated at the baseline visit if time between the most recent MRI and planned start of study treatment exceeds 3 months or if the results provide any reason to repeat it. Study treatment consists of 18 subcutaneous injections administered every 28 days. Follow-up visits are planned at week 24 and week 48 (after the 6th and 12th injection, respectively), and the end-of-study visit takes place at week 72, approximately 4 weeks after the last injection. Study duration from first injection to last visit is thus 72 weeks. In case of withdrawal, a premature end-of-study visit is planned in the month after the last received injection. Study visits take place at the centre of inclusion (AMC, VUMC or LUMC) and will coincide with regular care assessments where possible. See **Figure 3** for the complete study schedule.

For peer review only - http://bmjopen.bmj.com/site/about/guidelines.xhtml

^{68}Ga-DOTATATE PET/CT

^{68}Ga-DOTATATE PET/CT of the head is performed at either the AMC or the Netherlands Cancer Institute (Gemini ToF, Philips Medical Systems and Biograph mCT, Siemens Healthineers). ^{68}Ga-DOTATATE preparation and quality control is performed according to a standard protocol as described elsewhere.[26] Images are acquired approximately 45 minutes after intravenous bolus injection of 100 MBq ^{68}Ga-DOTATATE with 2.5-3 min per bed position. A low-dose CT scan is acquired for attenuation correction and anatomical correlation. The total estimated radiation exposure of the combined PET/CT is 3.1 mSv.[27] PET/CT and pituitary MR images are co-registered manually using Hybrid Viewer (Hermes Medical Solutions). On the fused PET/MR images, ^{68}Ga-DOTATATE uptake is assessed by placement of a circular region of interest (ROI) within the adenoma while maintaining a clear margin from normal pituitary tissue, and determining the mean standard uptake value (SUV_{mean}).[23] An SUV_{mean} of >2 is considered as positive uptake, based on a ^{68}Ga-DOTATATE biodistribution study in which physiological pituitary uptake always had an SUV_{mean} of >2.[28]

Intervention

Forty-four patients with a ^{68}Ga-DOTATATE PET-positive NFMA are randomised between treatment with either the extended-release formulation of lanreotide 120 mg (Somatuline AutoSolution®, Ipsen Farmaceutica BV; known outside the Netherlands as Somatuline Autogel®) without dose titration or placebo (consisting of 0.4 mL saline 0.9%), both administered every 28 days as a deep subcutaneous injection in the superior, external quadrant of the buttock. Study treatment consists of a total of 18 injections. The injections are administered by experienced and trained nurses, either at the AMC endocrine unit or at home via a homecare service (Eurocept Homecare). Administration details are recorded at each nurse visit. Lanreotide 120 mg is the highest dose currently available. Treatment with this dose has shown to be safe and well-tolerated in two efficacy studies in enteropancreatic neuroendocrine tumours and acromegaly.[29,30] A safety review on SSA dose optimisation and a phase 2 RCT specifically investigating lanreotide in different dosages did not demonstrate significant changes in adverse effects and tolerability with increasing dose. Higher doses were, however, related to greater efficacy.[31,32]

Randomisation

Randomisation to treatment is done by means of a computer-generated randomisation list through Sealed Envelope Ltd (https://www.sealedenvelope.com/simple-randomiser/v1/lists) using blocked randomisation with an allocation ratio of 1:1 and block size of four.

Allocation concealment and blinding

Randomisation is performed by the AMC trial pharmacy. The randomisation list is stored in the pharmacy trial file and will not be disclosed until the last participant has completed the trial. In case of emergency the randomisation list can be accessed by the hospital pharmacist on call and the randomisation code can be broken if the safety of the patient requires knowledge of the assigned treatment (e.g. in case of a suspected unexpected serious adverse reaction (SUSAR)). Breaking of the code will be requested by an independent trial staff member to maintain allocation concealment to investigators assessing the outcomes. The study is blinded for participants, investigators and treating physicians. Because the placebo injections differ in appearance from the lanreotide (Somatuline AutoSolution®) pre-filled syringes, pharmacy employees preparing the treatment and nurses administering the injections cannot be blinded.[29] As the injections are administered into the

For peer review only - http://bmjopen.bmj.com/site/about/guidelines.xhtml

superior, external quadrant of the buttock, patients will not see the syringes. Furthermore, patients have no earlier experience with lanreotide treatment to have expectations concerning the administration. To maintain blinding during transport and storage, the study medication is placed in an opaque bag within a sealed cardboard box with a "blinded study medication" warning message.

Assessments during the study

Pituitary MRI

Pituitary MRIs are performed regularly as part of standard care of NFMA patients. Study participation is timed in such a way that visits coincide with regular MRI planning where possible. MRI scans are performed on a 1.5 or 3 Tesla scanner, following a pituitary-specific protocol that includes T1-weighted coronal and sagittal acquisitions before and after gadolinium administration, a T2-weighted coronal sequence, and preferably a volumetric 3DT1-weighted sequence.

Laboratory tests

Regular laboratory assessments are also part of standard care. During the study, laboratory tests will include an endocrine evaluation (TSH, free T4, cortisol, IGF-1, prolactin, and testosterone or oestradiol), fasting glucose, glycated haemoglobin (HbA1c), sodium, potassium, kidney function, liver enzymes, and bilirubin. At baseline and week 72, additional blood samples are obtained for alpha subunit assessment. Leftover serum and plasma will be stored according to local regulatory guidelines until publication of results.

Physical examination

Measurement of height, weight, blood pressure and pulse rate takes place at each visit after randomisation. More extensive examination is only performed if indicated.

Quality of life questionnaire

The 36-item Short Form Health Survey (SF-36), validated Dutch translation, is used to assess quality of life at study visits.[33] The SF-36 is comprised of 36 items that generate 8 component scores: physical functioning, role limitations due to physical health problems, bodily pain, general health perceptions, vitality, social functioning, role limitations due to emotional problems, and general mental health. Scores are expressed on a 0-100 scale with higher scores indicating better quality of life for that component.

Structured interview

The structured interview at visits during treatment consists of health-related questions based on possible side-effects of lanreotide, other signs and symptoms experienced between visits, and changes in medication use.

Adverse events

Adverse events (AEs) can be reported throughout the study period and will be inquired about specifically at study and nurse visits. An AE is defined as any undesirable finding or experience occurring to a subject during the study, whether or not considered related to study treatment. The type of AE, times of onset and resolution, severity, and possible relationship with lanreotide will be recorded.

For peer review only - http://bmjopen.bmj.com/site/about/guidelines.xhtml
For peer review only

Concomitant care and post-trial follow-up

Participants are not allowed to use medication or participate in other trials aimed at adenoma reduction. Otherwise, patient care is continued as usual. Participants may withdraw from the study for any reason at any time. In case of development of visual field impairments or signs pointing to cranial nerve injury, patients will undergo additional pituitary MRI and visual field examination as is standard practice. If intervention is deemed necessary, study treatment is stopped. Patients can also be withdrawn based on the investigator's or treating specialist's judgement, e.g. in case of an AE that could jeopardise patient safety. Premature study withdrawal will have no effect on regular patient care.

Lanreotide can inhibit the secretion of insulin and glucagon. Patients on antidiabetic therapy are therefore advised to monitor their blood glucose levels more closely after start of study treatment and have their medication adjusted accordingly. Concomitant use of bradycardia inducing drugs (e.g. beta blockers) may have an additive effect on the slight reduction of heart rate associated with lanreotide and dose adjustments may be necessary. Intestinal absorption of most notably ciclosporin can be reduced, requiring close review of therapeutic levels. Participants are instructed to notify the research team if they have been prescribed a new drug by their general practitioner or specialist during the study.

After completion of study treatment or premature withdrawal, standard care is continued. Patients who leave the study for medical reasons/AEs will be followed until the interfering condition has resolved or reached a stable state. All participants will be followed until completion of the trial and will be notified of the results.

Primary outcome

The primary outcome is the absolute change in cranio-caudal tumour diameter in millimetres from baseline to week 72 (or last available post-baseline measurement). Tumour size will be determined on pituitary MRI by two neuroradiologists blinded to treatment allocation and scan chronology. The measurements of both radiologists are averaged for the analysis. In case of a between-reader difference of ≥10% or ≥2mm, the images will be offered for reassessment. In case of a persistent difference of ≥10% or ≥2mm, a third neuroradiologist will review the images and the measurements of the three radiologists are averaged for the analysis. A change in tumour size of ≥2mm is considered clinically relevant, as such a difference is sufficient to prevent complications related to tumour growth. Even a small increase in cranio-caudal diameter can be reason for intervention via surgery or radiotherapy to prevent or relieve compression of the optic nerves or chiasm.[34] Moreover, the value of 2mm is commonly used as cut-off to reliably detect a change in tumour size on pituitary MRI.[35–37]

Secondary outcomes

Change in tumour volume

As with the primary outcome, the change in tumour volume between baseline and week 72 (or last available post-baseline measurement) is determined by two neuroradiologists or trained and supervised study personnel using pituitary MR images. Tumour volume in cubic millimetres will be calculated using a semi-automatic segmentation tool in the coronal plane. On each slice the tumour is delineated and the surface area multiplied by the slice thickness. The total tumour volume is

For peer review only - http://bmjopen.bmj.com/site/about/guidelines.xhtml

determined by summing up the slice volumes. The slice-by-slice segmentation method is preferred to the traditional geometric formula (i.e. ½ x length x width x height) due to the frequent irregular shape and invasion of NFMA, especially if it concerns a postoperative remnant.[38] In case of a between-reader difference of ≥10%, the images will be offered for reassessment. In case of a persistent difference of ≥10%, a third neuroradiologist or experienced study staff member will review the images. A change in tumour volume of ≥20% will be considered clinically significant.[30]

Time to progression

The presence or absence of tumour progression will be based on change in tumour volume between baseline and subsequent pituitary MRIs. Tumour progression is defined as an increase in volume of ≥20%, absence of progression comprises both tumour shrinkage (volume decrease ≥20%) and stable disease (volume change <20%).

Change in quality of life

The change in quality of life is based on the SF-36 questionnaire component scores obtained at the study visits.

Number of (serious) AEs

As a safety endpoint, the number and type of AEs and serious AEs (SAEs) recorded during the study will be compared between the groups.

Sample size

The study is powered on a between-group difference in primary outcome of ≥2mm. In order to detect a mean difference of 2mm with a standard deviation of 1.9mm, we need to randomise 16 patients per group based on a 2-sided independent *t*-test with 80% power and 5% type I error risk (nQuery Advisor 7.0, Statsol, Boston, Massachusetts, USA). The final analysis of the primary endpoint will be performed by ANCOVA. While ANCOVA may reduce the required sample size, a reliable estimation of rho is not possible for this study and a more conservative *t*-test-based power analysis is therefore less likely to result in an underpowered study.[39] Taking into account an observed dropout rate of around 25%, as a result of withdrawals due to AEs or need for intervention, the sample size has been increased to 22 patients per group (substantial amendment, protocol version 5.0). Dropout is defined as failure to complete the 72 weeks of treatment due to any reason. Only patients with a ^{68}Ga-DOTATATE PET-positive NFMA are randomised for treatment. Based on previous reports that about two-thirds of NFA patients showed increased adenoma uptake using ^{111}In-DTPA-octreotide scintigraphy[10,14,15,40] and considering the superior sensitivity of ^{68}Ga-DOTATATE PET/CT, we expected to enrol a maximum of 66 patients in order to randomise 44 patients.

Statistical analyses

Data of continuous variables will be summarised as either means with standard deviation or medians with interquartile range, according to distribution. Normality of distribution will be assessed visually with histograms, Q-Q plots and using the Shapiro-Wilk test. Data of categorical variables will be reported as incidence rates (number and percentage). Baseline characteristics and ^{68}Ga-DOTATATE PET/CT results of all included subjects will be reported. Data on recruitment and dropout will also be reported. Differences between patients with positive and negative ^{68}Ga-DOTATATE uptake will be evaluated. Group differences in continuous variables are analysed using the Student's *t*-test or Mann-Whitney U test, depending on data distribution. Group differences in categorical variables are

For peer review only - http://bmjopen.bmj.com/site/about/guidelines.xhtml

analysed using either the Chi-square or the Fisher's exact test, as appropriate. In principle, since patients are randomly allocated to treatment, differences in baseline characteristics between the groups should not be analysed. When performing linear regression analyses or linear mixed effects models, assumptions for linearity, distribution and/or homoscedasticity will be checked for each model using scatter plots and residual analysis.

Outcome data will be analysed according to intention-to-treat (ITT). The ITT population is defined as all randomised patients having received at least one study injection. In case of withdrawal after treatment initiation, effort is made to plan a premature end-of-study visit to obtain outcome data for the ITT analyses, unless assessments were performed within the previous 4 weeks. In case of incomplete/missing outcome data, the assumption that data is missing at random (MAR) will be checked. If the MAR assumption is plausible, a main analysis using all observed data is performed (e.g. a mixed model). This main analysis does not require the inclusion of subjects that lack post-baseline assessments.[41] Sensitivity analyses including all ITT subjects are then performed to explore the impact of departures from this assumption.[41] This includes an analysis using multiple imputation in case of missing outcome data.[42]

The difference between groups in primary outcome and secondary outcome change in tumour volume will be analysed using one-way ANCOVA, controlling for baseline adenoma size. For these two endpoints, a separate per-protocol efficacy analysis will be performed, including only those subjects that have completed all 18 study injections and final MRI. Additionally, the proportion of patients with a clinically significant change in tumour size (i.e. ≥2mm change in cranio-caudal size or ≥20% change in tumour volume) is reported. Time to tumour progression is analysed using the Kaplan-Meier method. Missing cases due to withdrawal without outcome data will be censored. Differences in distribution and percentage of censoring between groups will be evaluated. Between-group differences in time to progression are analysed using the stratified logrank test, with stratification for presence or absence of tumour growth at baseline. To quantify the difference, the hazard ratio and confidence intervals are estimated using the Cox proportional-hazards model. The SF-36 component scores will be presented as spider charts. The mean imputation method will be used to replace missing values. Results will be compared with Dutch population reference values[33] and the change in relevant component scores are compared between the groups. All patients that received at least one study injection will be included in the safety endpoint analysis of AEs independent of post-baseline assessments.

As the study remains blinded until completion, no interim analyses are planned. There are no pre-specified subgroups. A *p*-value <0.05 will be considered statistically significant. In case of multiple testing, Bonferroni correction will be applied where appropriate. Besides the reporting of *p*-values, 95% confidence intervals around point estimates will be provided for more useful interpretation of results. Analyses will be done using SPSS (IBM, v25 or later if available) and/or RStudio (R Foundation for Statistical Computing, v3.6.1, 2019-07-05, or later if available). Before outcome analyses are initiated, the statistical analysis plan will be finalised.

Data management

Data is collected using Castor Electronic Data Capture (EDC).[43] The electronic case report forms (CRFs) have built-in data validity and value range checks. Source documents include medical records, records of the Eurocept homecare service, SF-36 questionnaires and paper CRFs documenting

For peer review only - http://bmjopen.bmj.com/site/about/guidelines.xhtml

injections administered at the AMC. All subject data is pseudonymised with a study code. The subject identification log which links subjects to the code is kept in a trial file only accessible to study personnel. All research data will be stored for 15 years. Leftover serum and plasma from blood samples obtained for standard care during the study will be stored according to local regulatory guidelines until publication of the results and then destroyed. Data will be handled in accordance with the principles of the General Data Protection Regulation. All data management procedures are detailed in a separate data management plan.

Safety

The most commonly expected side-effects of lanreotide are gastrointestinal disorders (e.g. diarrhoea, abdominal pain and nausea, usually mild/moderate and transient), cholelithiasis (often asymptomatic) and injection site reactions. Additionally, lanreotide may induce (mild) hypoglycaemia or hyperglycaemia due to inhibition of insulin and/or glucagon secretion. Because of surveillance at regular intervals during the study the risks are small. Throughout the study, AEs reported by the patient or observed by the investigators or healthcare providers are recorded in the medical file and study database. All SAEs including SUSARs are reported to the medical research ethics committee (MREC) of the AMC. In addition, an annual safety report is submitted.

Monitoring

The trial is monitored by the Clinical Research Unit of the AMC. Participating centres agree to allow monitoring by providing access to source data and documents as required. Every participating centre is subjected to a start-up visit after three included patients, a second site-visit after approximately ten inclusions and a combined monitoring and site close-out visit after completion of data collection. The monitor confirms all written informed consents, checks all SAEs and investigates data collection and data quality for a random subset of patients. The frequency and intensity of monitoring can be changed based on findings during visits. Monitoring procedures are detailed in a separate monitoring plan.

Patient and public involvement

Patients were not involved in the design of this study. The study was highlighted in “Hyponieuws”, the magazine of the Dutch Pituitary Foundation, which is the Dutch pituitary patient platform.

ETHICS AND DISSEMINATION

The study protocol has been approved by the accredited MREC of the AMC and by the Dutch competent authority. Permission for execution of the study is obtained at each participating site. Substantial amendments to the protocol are submitted for approval and communicated to participating centres and trial registries. The study is conducted in accordance with the Declaration of Helsinki, Good Clinical Practice guidelines, and local regulatory requirements. Participant insurance is provided by the AMC for all participating subjects.

Regardless of the outcome, study results will be submitted for publication in a peer-reviewed international medical journal and presented at (inter)national conferences. The International Committee of Medical Journal Editors guidelines for authorship will be followed. The AMC, as

For peer review only - http://bmjopen.bmj.com/site/about/guidelines.xhtml

sponsor, will remain owner of all data and have all publication rights. Full protocol, datasets and statistical analysis plan will be available from the corresponding author on reasonable request after publication of the final results.

Trial status

Recruitment started on November 3rd 2015. At the time of preparing this manuscript, inclusion has been completed. The last visit of the last patient is expected in the first half of 2021.

DISCUSSION

The GALANT study is the first double-blind and placebo-controlled intervention trial in NFMA patients, investigating the effect of lanreotide 120 mg on NFMA size. Treatment duration is 72 weeks, in line with the slow average NFA growth rate of around 1 mm/year.[4,35] An additional strength of the study design is the use of ^{68}Ga-DOTATATE PET/CT to select only those patients with an SSTR-positive adenoma for treatment. Although in a clinical setting dose titration might mitigate side-effects and prevent treatment cessation, use of the highest dose currently available is justified in order to prove efficacy for a possible new therapeutic indication and ensures data homogeneity. Furthermore, treatment with this dose has shown to be safe and well-tolerated in earlier trials.[29–31] The study is powered to detect a between-group difference of ≥2 mm change in cranio-caudal tumour size after treatment, a clinically relevant outcome. Consistent and unbiased tumour measurements are ensured through assessment by at least two neuroradiologists blinded for treatment allocation and scan chronology. If lanreotide proves effective in reducing or controlling tumour size, this would present an important new treatment option for NFMA patients.

Supplementary files

The following supplementary files to this article can be found online at ***[… fill-in-DOI]***:
Supplementary file S1: Completed SPIRIT Checklist. (PDF, 184 kB)
Supplementary file S2: Patient information letter and informed consent form, Dutch AMC version. (PDF, 606 kB)
Supplementary file S3: Patient information letter and informed consent form, English AMC version. (PDF, 344 kB)

Footnotes

Contributors: TB, MD, JB, CM, MS, MT, PB and EF participated in the design of the study. EF, MD, MS and AP are principal investigators at the particpating study centres. EF supervises the study in name of the sponsor (AMC). MD, JH, AP, NB, SS, RGV, PB and EF assist with the referral and inclusion of patients. TB drafted the manuscript with help of PB and EF. All authors reviewed and approved the final manuscript.

Funding: This work is supported by Ipsen Farmaceutica BV (the Netherlands). The GALANT study is investigator-initiated; the Academic Medical Centre (AMC) is the sponsor of the study. The funding

For peer review only - http://bmjopen.bmj.com/site/about/guidelines.xhtml

party has played no role in the final study design or study conduct nor in the preparation of this manuscript or the decision to submit it for publication. The AMC, as sponsor, will remain owner of all data and have all publication rights.

Competing interests: MD received a honorarium for chairing a symposium sponsored by Ipsen Farmaceutica BV in 2019. No other potential conflicts of interest have been declared.

Ethics approval: Medical Ethics Committee Academic Medical Centre, of the Amsterdam University Medical Centres (reference: NL 52821.018.15).

Trial registration: EudraCT: 2015-001234-22, registered 10 March 2015; Netherlands Trial Register (NTR): NL5136, registered 18 August 2015. Registration took place before start of recruitment.

Protocol versions:

- V2.0, May 21st 2015: first approved protocol version.
- V3.0, October 13th 2016: new participating centre (LUMC) added to achieve target recruitment goal; possibility to perform the ^{68}Ga-DOTATATE PET/CT at the AMC; possibility for patients to have study injections administered at home by a specialised homecare company with trained nurses (Eurocept Homecare).
- V4.0, August 31st 2017: clearer exclusion criterion concerning dopamine agonist use: "Use of dopamine agonists" changed to "Use of dopamine agonist in the past 6 months".
- V5.0, November 30th 2018: increased number of randomisations to account for dropouts and more detailed statistical analysis section (including handling of missing/incomplete data). Current protocol version.

Data sharing statement: The datasets generated and/or analysed during the current study will be available from the corresponding author on reasonable request after publication of the final results.

Provenance and peer review: Not commissioned; externally peer reviewed.

Open Access: This is an Open Access article distributed in accordance with the Creative Commons Attribution Non Commercial (CC BY-NC 4.0) license, which permits others to distribute, remix, adapt, build upon this work non-commercially, and license their derivative works on different terms, provided the original work is properly cited and the use is non-commercial. See: https://creativecommons.org/licenses/by-nc/4.0/

References

1 Aflorei ED, Korbonits M. Epidemiology and etiopathogenesis of pituitary adenomas. *J Neurooncol* 2014;**117**:379–94. doi:10.1007/s11060-013-1354-5

2 Ntali G, Wass JA. Epidemiology, clinical presentation and diagnosis of non-functioning pituitary adenomas. *Pituitary* 2018;**21**:111–8. doi:10.1007/s11102-018-0869-3

3 Roelfsema F, Biermasz NR, Pereira AM. Clinical factors involved in the recurrence of pituitary adenomas after surgical remission: A structured review and meta-analysis. *Pituitary* 2012;**15**:71–83. doi:10.1007/s11102-011-0347-7

4 Chen Y, Wang C De, Su ZP, *et al.* Natural History of Postoperative Nonfunctioning Pituitary Adenomas: A

For peer review only - http://bmjopen.bmj.com/site/about/guidelines.xhtml

Systematic Review and Meta-Analysis. *Neuroendocrinology* 2012;**96**:333–42. doi:10.1159/000339823

5 Sheehan J, Lee C-C, Bodach ME, *et al.* Congress of Neurological Surgeons Systematic Review and Evidence-Based Guideline for the Management of Patients With Residual or Recurrent Nonfunctioning Pituitary Adenomas. *Neurosurgery* 2016;**79**:E539–40. doi:10.1227/NEU.0000000000001385

6 Cortet-Rudelli C, Bonneville J-F, Borson-Chazot F, *et al.* Post-surgical management of non-functioning pituitary adenoma. *Ann Endocrinol (Paris)* 2015;**76**:228–38. doi:10.1016/j.ando.2015.04.003

7 Minniti G, Flickinger J, Tolu B, *et al.* Management of nonfunctioning pituitary tumors: radiotherapy. *Pituitary* 2018;**21**:154–61. doi:10.1007/s11102-018-0868-4

8 Dekkers OM, Pereira AM, Romijn JA. Treatment and follow-up of clinically nonfunctioning pituitary macroadenomas. *J Clin Endocrinol Metab* 2008;**93**:3717–26. doi:10.1210/jc.2008-0643

9 Greenman Y. MANAGEMENT OF ENDOCRINE DISEASE: Present and future perspectives for medical therapy of nonfunctioning pituitary adenomas. *Eur J Endocrinol* 2017;**177**:R113–24. doi:10.1530/EJE-17-0216

10 Fusco A, Giampietro A, Bianchi A, *et al.* Treatment with octreotide LAR in clinically non-functioning pituitary adenoma: Results from a case-control study. *Pituitary* 2012;**15**:571–8. doi:10.1007/s11102-011-0370-8

11 Lee M, Lupp A, Mendoza N, *et al.* SSTR3 is a putative target for the medical treatment of gonadotroph adenomas of the pituitary. *Endocr Relat Cancer* 2015;**22**:111–9. doi:10.1530/ERC-14-0472

12 Ramírez C, Cheng S, Vargas G, *et al.* Expression of Ki-67, PTTG1, FGFR4, and SSTR 2, 3, and 5 in Nonfunctioning Pituitary Adenomas: A High Throughput TMA, Immunohistochemical Study. *J Clin Endocrinol Metab* 2012;**97**:1745–51. doi:10.1210/jc.2011-3163

13 Gabalec F, Drastikova M, Cesak T, *et al.* Dopamine 2 and somatostatin 1-5 receptors coexpression in clinically non-functioning pituitary adenomas. *Physiol Res* 2015;**64**:369–77.http://www.ncbi.nlm.nih.gov/pubmed/25536318

14 Plöckinger U, Bäder M, Hopfenmüller W, *et al.* Results of somatostatin receptor scintigraphy do not predict pituitary tumor volume- and hormone-response to ocreotide therapy and do not correlate with tumor histology. *Eur J Endocrinol* 1997;**136**:369–76.http://www.ncbi.nlm.nih.gov/pubmed/9150695

15 Borson-Chazot F, Houzard C, Ajzenberg C, *et al.* Somatostatin receptor imaging in somatotroph and non-functioning pituitary adenomas: correlation with hormonal and visual responses to octreotide. *Clin Endocrinol (Oxf)* 1997;**47**:589–98. doi:10.1046/j.1365-2265.1997.3361119.x

16 Reubi JC, Waser B, Schaer J-C, *et al.* Somatostatin receptor sst1-sst5 expression in normal and neoplastic human tissues using receptor autoradiography with subtype-selective ligands. *Eur J Nucl Med Mol Imaging* 2001;**28**:836–46. doi:10.1007/s002590100541

17 Bevan JS. The Antitumoral Effects of Somatostatin Analog Therapy in Acromegaly. *J Clin Endocrinol Metab* 2005;**90**:1856–63. doi:10.1210/jc.2004-1093

18 Colao A, Di Somma C, Pivonello R, *et al.* Medical therapy for clinically non-functioning pituitary adenomas. *Endocr Relat Cancer* 2008;**15**:905–15. doi:10.1677/ERC-08-0181

19 Melmed S. Pituitary Medicine From Discovery to Patient-Focused Outcomes. *J Clin Endocrinol Metab* 2016;**101**:769–77. doi:10.1210/jc.2015-3653

20 Colao A, Lastoria S, Ferone D, *et al.* The pituitary uptake of 111In-DTPA-D-Phe1-octreotide in the normal pituitary and in pituitary adenomas. *J Endocrinol Invest* 1999;**22**:176–83.http://www.embase.com/search/results?subaction=viewrecord&from=export&id=L29158636

21 de Herder WW, Kwekkeboom DJ, Feelders RA, *et al.* Somatostatin receptor imaging for neuroendocrine tumors. *Pituitary* 2006;**9**:243–8. doi:10.1007/s11102-006-0270-5

22 Reubi JC, Schaer J-C, Waser B, *et al.* Affinity profiles for human somatostatin receptor subtypes SST1-SST5 of somatostatin radiotracers selected for scintigraphic and radiotherapeutic use. *Eur J Nucl Med Mol Imaging* 2000;**27**:273–82.http://www.ncbi.nlm.nih.gov/pubmed/10774879

23 Bai B, Bading J, Conti PS. Tumor quantification in clinical positron emission tomography. *Theranostics* 2013;**3**:787–801. doi:10.7150/thno.5629

24 Chan A-W, Tetzlaff JM, Gøtzsche PC, *et al.* SPIRIT 2013 explanation and elaboration: guidance for protocols of clinical trials. *BMJ* 2013;**346**:e7586–e7586. doi:10.1136/bmj.e7586

25 Chan A-W, Tetzlaff JM, Altman DG, *et al.* SPIRIT 2013 Statement: Defining Standard Protocol Items for Clinical Trials. *Ann Intern Med* 2013;**158**:200. doi:10.7326/0003-4819-158-3-201302050-00583

26 Aalbersberg EA, Geluk-Jonker MM, Young-Mylvaganan T, *et al.* A Practical Guide for the Production and PET/CT Imaging of 68Ga-DOTATATE for Neuroendocrine Tumors in Daily Clinical Practice. *J Vis Exp* 2019;:1–2. doi:10.3791/59358

27 Walker RC, Smith GT, Liu E, *et al.* Measured human dosimetry of 68Ga-DOTATATE. *J Nucl Med*

For peer review only

For peer review only - http://bmjopen.bmj.com/site/about/guidelines.xhtml

2013;**54**:855–60. doi:10.2967/jnumed.112.114165
28 Shastry M, Kayani I, Wild D, *et al.* Distribution pattern of 68Ga-DOTATATE in disease-free patients. *Nucl Med Commun* 2010;**31**:1. doi:10.1097/MNM.0b013e32833f635e
29 Caplin ME, Pavel M, Cwikła JB, *et al.* Lanreotide in metastatic enteropancreatic neuroendocrine tumors. *N Engl J Med* 2014;**371**:224–33. doi:10.1056/NEJMoa1316158
30 Caron PJ, Bevan JS, Petersenn S, *et al.* Tumor shrinkage with lanreotide autogel 120 mg as primary therapy in acromegaly: Results of a prospective multicenter clinical trial. *J Clin Endocrinol Metab* 2014;**99**:1282–90. doi:10.1210/jc.2013-3318
31 Ludlam WH, Anthony L. Safety review: Dose optimization of somatostatin analogs in patients with acromegaly and neuroendocrine tumors. *Adv Ther* 2011;**28**:825–41. doi:10.1007/s12325-011-0062-9
32 Bronstein M, Musolino N, Jallad R, *et al.* Pharmacokinetic profile of lanreotide Autogel?? in patients with acromegaly after four deep subcutaneous injections of 60, 90 or 120 mg every 28 days. *Clin Endocrinol (Oxf)* 2005;**63**:514–9. doi:10.1111/j.1365-2265.2005.02372.x
33 Aaronson NK, Muller M, Cohen PDA, *et al.* Translation, validation, and norming of the Dutch language version of the SF-36 Health Survey in community and chronic disease populations. *J Clin Epidemiol* 1998;**51**:1055–68. doi:10.1016/S0895-4356(98)00097-3
34 Molitch ME. Diagnosis and Treatment of Pituitary Adenomas. *JAMA* 2017;**317**:516. doi:10.1001/jama.2016.19699
35 Dekkers OM, Hammer S, de Keizer RJW, *et al.* The natural course of non-functioning pituitary macroadenomas. *Eur J Endocrinol* 2007;**156**:217–24. doi:10.1530/eje.1.02334
36 O'Sullivan EP, Woods C, Glynn N, *et al.* The natural history of surgically treated but radiotherapy-naïve nonfunctioning pituitary adenomas. *Clin Endocrinol (Oxf)* 2009;**71**:709–14. doi:10.1111/j.1365-2265.2009.03583.x
37 Brochier S, Galland F, Kujas M, *et al.* Factors predicting relapse of nonfunctioning pituitary macroadenomas after neurosurgery: A study of 142 patients. *Eur J Endocrinol* 2010;**163**:193–200. doi:10.1530/EJE-10-0255
38 Chuang C-C, Lin S-Y, Pai P-C, *et al.* Different Volumetric Measurement Methods for Pituitary Adenomas and Their Crucial Clinical Significance. *Sci Rep* 2017;**7**:40792. doi:10.1038/srep40792
39 Borm GF, Fransen J, Lemmens WAJG. A simple sample size formula for analysis of covariance in randomized clinical trials. *J Clin Epidemiol* 2007;**60**:1234–8. doi:10.1016/j.jclinepi.2007.02.006
40 Duet M, Ajzenberg C, Benelhadj S, *et al.* Somatostatin receptor scintigraphy in pituitary adenomas: a somatostatin receptor density index can predict hormonal and tumoral efficacy of octreotide in vivo. *J Nucl Med* 1999;**40**:1252–6.http://www.ncbi.nlm.nih.gov/pubmed/10450674
41 White IR, Carpenter J, Horton NJ. Including all individuals is not enough: Lessons for intention-to-treat analysis. *Clin Trials* 2012;**9**:396–407. doi:10.1177/1740774512450098
42 Sterne JAC, White IR, Carlin JB, *et al.* Multiple imputation for missing data in epidemiological and clinical research: potential and pitfalls. *BMJ* 2009;**338**:b2393. doi:10.1136/bmj.b2393
43 Castor E. Castor Electronic Data Capture. 2019.https://castoredc.com

For peer review only

For peer review only - http://bmjopen.bmj.com/site/about/guidelines.xhtml

Figure legends

Figure 1 Flowchart of the GALANT (a randomized placebo-controlled trial in patients with a ^{68}*Ga*-DOTATATE PET-positive, clinically nonfunctioning pituitary macroadenoma on the effect of *lan*reotide on *t*umour size) study. NFMA, nonfunctioning pituitary macroacdenoma

Figure 2 Eligibility criteria.

Figure 3 Standard Protocol Items: Recommendations for Interventional Trials (SPIRIT) figure. Schedule of enrolment, interventions, and assessments in the GALANT study. SF-36, 36-item Short Form Health Survey.

[a] Treatment consists of 18 injections administered every 28 days.

[b] In case of study withdrawal, a premature end-of-study visit is planned in the month after the last received injection.

[c] Patient age, gender, medication use, most recent laboratory and pituitary MRI results, current pituitary hormone deficiencies, prior treatment and further relevant medical history.

[d] Only in female patients of childbearing potential.

[e] Pituitary MRI is only repeated if time between the most recent MRI and planned start of study treatment exceeds three months or if results give cause to repeat it.

[f] Endocrine evaluation, fasting glucose, glycated haemoglobin (HbA1c), sodium, potassium, kidney function, liver enzymes, and bilirubin; additional sample at baseline and close-out for alpha subunit assessment.

[g] Measurement of height, weight, blood pressure and pulse rate; more extensive examination if indicated.

[h] Unresolved adverse events at time of end-of-study visit are followed up until resolved or stabilised.

For peer review only - http://bmjopen.bmj.com/site/about/guidelines.xhtml

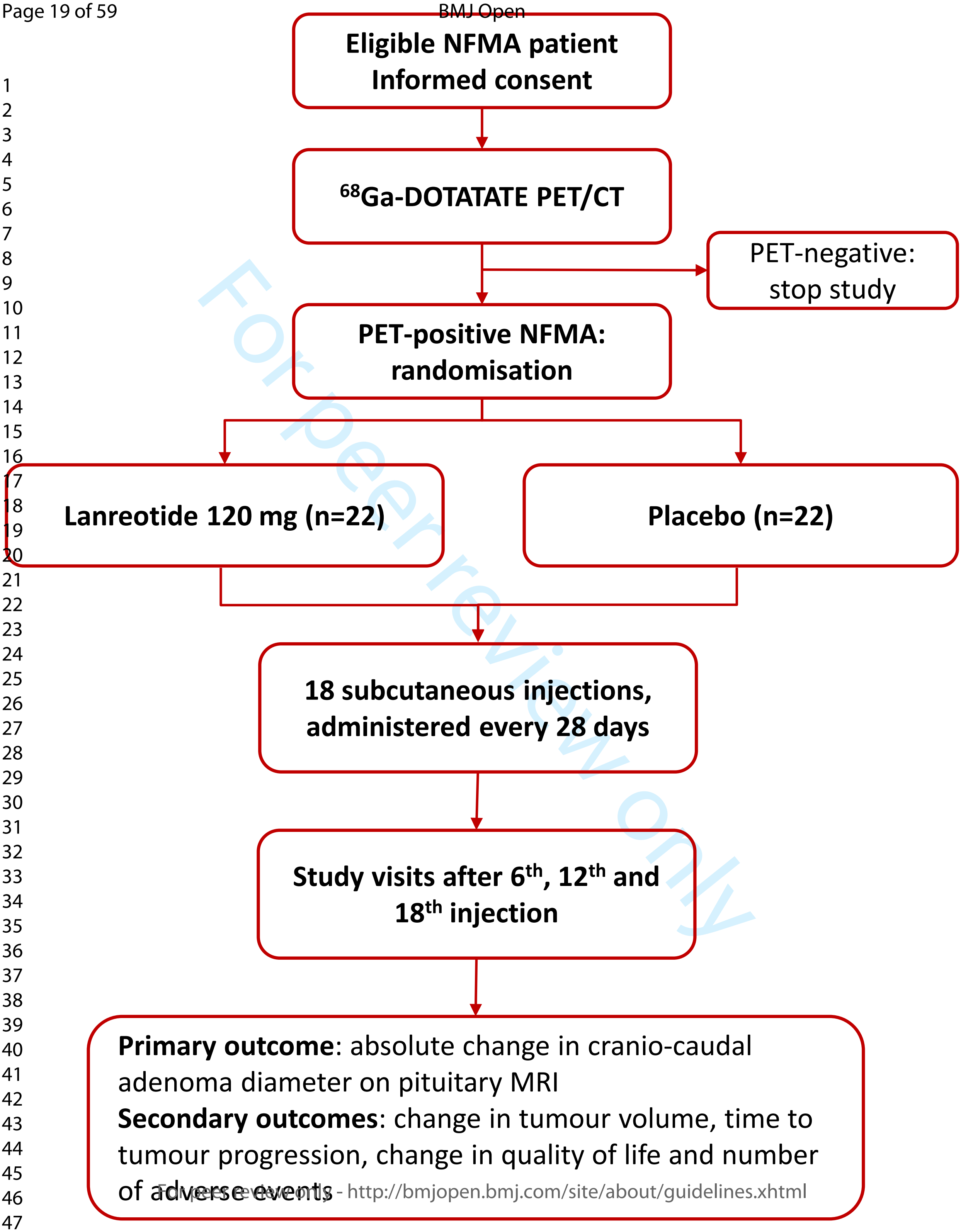

For peer review only

Inclusion criteria

- Clinically nonfunctioning pituitary macroadenoma extending above the sellar diaphragm
- Age ≥ 18 years
- Written informed consent

Exclusion criteria

- Optic chiasm compression with visual field defects
- Previous radiation therapy in the pituitary region
- Previous use of somatostatin analogues
- Use of dopamine receptor agonists in the past 6 months
- Hypersensitivity to somatostatin or similar peptides
- Obstructive neuroendocrine gut tumour
- Symptomatic cholelithiasis
- Pregnancy, lactation or wish to conceive
- Female patients of childbearing potential not using adequate contraception
- Any contraindication to perform MRI with gadolinium-based contrast agent (including implanted stimulation devices and severe claustrophobia)

For peer review only

	STUDY PERIOD						
	Screen	Baseline	Treatment[a]				Close-out[b]
**Weeks**		-1 / 0	0	24	48	68	72
**Visits**	V_1	V_2		V_3	V_4		V_5
**ENROLMENT:**							
**Eligibility screen**	X						
**Informed consent**	X						
**Baseline characteristics**[c]	X						
**Pregnancy test, if applicable**[d]	X	X					
**^{68}Ga-DOTATATE PET/CT**	X						
**Allocation**		X					
**INTERVENTIONS:**							
**Lanreotide**			◆———	———	———	———◆	
**Placebo**			◆———	———	———	———◆	
**ASSESSMENTS:**							
**Pituitary MRI**		X[e]		X			X
**Laboratory assessment**[f]		X		X	X		X
**Physical examination**[g]		X		X	X		X
**SF-36 survey**		X		X	X		X
**Concomitant medication**		X		X	X		X
**Adverse events**		X	◆———	———	———	———	———◆ X[h]

For peer review only

SPIRIT
STANDARD PROTOCOL ITEMS: RECOMMENDATIONS FOR INTERVENTIONAL TRIALS

Manuscript Boertien et al. "The GALANT trial: study protocol of a randomized placebo-controlled trial in patients with a ^{68}Ga-DOTATATE PET/positive, clinically nonfunctioning pituitary macroadenoma on the effect of lanreotide on tumour size" – v 04-MAR-2020

SPIRIT 2013 Checklist: Recommended items to address in a clinical trial protocol and related documents*

Section/item	Item No	Description	Addressed in section, page no.
**Administrative information**			
Title	1	Descriptive title identifying the study design, population, interventions, and, if applicable, trial acronym	Title page, p1
Trial registration	2a	Trial identifier and registry name. If not yet registered, name of intended registry	Abstract, p2
	2b	All items from the World Health Organization Trial Registration Data Set	N/A (see registry)
Protocol version	3	Date and version identifier	Footnotes, p13
Funding	4	Sources and types of financial, material, and other support	Footnotes, p12-13
Roles and responsibilities	5a	Names, affiliations, and roles of protocol contributors	Title page, p1; Footnotes, p12
	5b	Name and contact information for the trial sponsor	Title page, p1
	5c	Role of study sponsor and funders, if any, in study design; collection, management, analysis, and interpretation of data; writing of the report; and the decision to submit the report for publication, including whether they will have ultimate authority over any of these activities	Ethics&Dissemination, p11-12; Footnotes, p12-13

For peer review only

For peer review only - http://bmjopen.bmj.com/site/about/guidelines.xhtml

	5d	Composition, roles, and responsibilities of the coordinating centre, steering committee, endpoint adjudication committee, data management team, and other individuals or groups overseeing the trial, if applicable (see Item 21a for data monitoring committee)	N/A
**Introduction**			
Background and rationale	6a	Description of research question and justification for undertaking the trial, including summary of relevant studies (published and unpublished) examining benefits and harms for each intervention	Introduction, p4
	6b	Explanation for choice of comparators	Intervention, p6
Objectives	7	Specific objectives or hypotheses	Introduction, p4
Trial design	8	Description of trial design including type of trial (eg, parallel group, crossover, factorial, single group), allocation ratio, and framework (eg, superiority, equivalence, noninferiority, exploratory)	Study design&setting, p5
**Methods: Participants, interventions, and outcomes**			
Study setting	9	Description of study settings (eg, community clinic, academic hospital) and list of countries where data will be collected. Reference to where list of study sites can be obtained	Study design&setting, p5
Eligibility criteria	10	Inclusion and exclusion criteria for participants. If applicable, eligibility criteria for study centres and individuals who will perform the interventions (eg, surgeons, psychotherapists)	Eligibility criteria, p5; Figure 2
Interventions	11a	Interventions for each group with sufficient detail to allow replication, including how and when they will be administered	Intervention p6; Allocation concealment…, p6-7
	11b	Criteria for discontinuing or modifying allocated interventions for a given trial participant (eg, drug dose change in response to harms, participant request, or improving/worsening disease)	Intervention, p6; Concomitant care…, p8
	11c	Strategies to improve adherence to intervention protocols, and any procedures for monitoring adherence (eg, drug tablet return, laboratory tests)	Intervention, p6
	11d	Relevant concomitant care and interventions that are permitted or prohibited during the trial	Concomitant care…, p8

For peer review only - http://bmjopen.bmj.com/site/about/guidelines.xhtml

Outcomes	12	Primary, secondary, and other outcomes, including the specific measurement variable (eg, systolic blood pressure), analysis metric (eg, change from baseline, final value, time to event), method of aggregation (eg, median, proportion), and time point for each outcome. Explanation of the clinical relevance of chosen efficacy and harm outcomes is strongly recommended	Primary outcome, p8; Secondary outcomes, p8-
Participant timeline	13	Time schedule of enrolment, interventions (including any run-ins and washouts), assessments, and visits for participants. A schematic diagram is highly recommended (see Figure)	Study outline, p5; Figure 3
Sample size	14	Estimated number of participants needed to achieve study objectives and how it was determined, including clinical and statistical assumptions supporting any sample size calculations	Sample size, p9
Recruitment	15	Strategies for achieving adequate participant enrolment to reach target sample size	Study outline, p5
**Methods: Assignment of interventions (for controlled trials)**			
Allocation:			
Sequence generation	16a	Method of generating the allocation sequence (eg, computer-generated random numbers), and list of any factors for stratification. To reduce predictability of a random sequence, details of any planned restriction (eg, blocking) should be provided in a separate document that is unavailable to those who enrol participants or assign interventions	Randomisation, p6
Allocation concealment mechanism	16b	Mechanism of implementing the allocation sequence (eg, central telephone; sequentially numbered, opaque, sealed envelopes), describing any steps to conceal the sequence until interventions are assigned	Randomisation, p6; Allocation concealment…, p6-7
Implementation	16c	Who will generate the allocation sequence, who will enrol participants, and who will assign participants to interventions	Allocation concealment…, p6-7
Blinding (masking)	17a	Who will be blinded after assignment to interventions (eg, trial participants, care providers, outcome assessors, data analysts), and how	Allocation concealment…, p6-7
	17b	If blinded, circumstances under which unblinding is permissible, and procedure for revealing a participant's allocated intervention during the trial	Allocation concealment…, p6-7
**Methods: Data collection, management, and analysis**			

For peer review only - http://bmjopen.bmj.com/site/about/guidelines.xhtml

Data collection methods	18a	Plans for assessment and collection of outcome, baseline, and other trial data, including any related processes to promote data quality (eg, duplicate measurements, training of assessors) and a description of study instruments (eg, questionnaires, laboratory tests) along with their reliability and validity, if known. **Reference to where data collection forms can be found, if not in the protocol**	Study outline, p5; Assessments…, p7; Outcomes, p8-9; Data management, p10-11
	18b	Plans to promote participant retention and complete follow-up, including list of any outcome data to be collected for participants who discontinue or deviate from intervention protocols	Study outline, p5; Statistical analyses, p9-10
Data management	19	Plans for data entry, coding, security, and storage, including any related processes to promote data quality (eg, double data entry; range checks for data values). **Reference to where details of data management procedures can be found, if not in the protocol**	Data management, p10-11
Statistical methods	20a	Statistical methods for analysing primary and secondary outcomes. Reference to where other details of the statistical analysis plan can be found, if not in the protocol	Statistical analyses, p9-10
	20b	Methods for any additional analyses (eg, subgroup and adjusted analyses)	Statistical analyses, p9-10
	20c	Definition of analysis population relating to protocol non-adherence (eg, as randomised analysis), and any statistical methods to handle missing data (eg, multiple imputation)	Statistical analyses, p9-10
**Methods: Monitoring**			
Data monitoring	21a	Composition of data monitoring committee (DMC); summary of its role and reporting structure; statement of whether it is independent from the sponsor and competing interests; and reference to where further details about its charter can be found, if not in the protocol. Alternatively, an explanation of why a DMC is not needed	N/A (no DMC), Monitoring, p11
	21b	Description of any interim analyses and stopping guidelines, including who will have access to these interim results and make the final decision to terminate the trial	N/A (no interim analyses)
Harms	22	Plans for collecting, assessing, reporting, and managing solicited and spontaneously reported adverse events and other unintended effects of trial interventions or trial conduct	Assessments.., p7; Safety, p11

Auditing	23	Frequency and procedures for auditing trial conduct, if any, and whether the process will be independent from investigators and the sponsor	Monitoring, p11
**Ethics and dissemination**			
Research ethics approval	24	Plans for seeking research ethics committee/institutional review board (REC/IRB) approval	Ethics&Dissemination, p11-12;
Protocol amendments	25	Plans for communicating important protocol modifications (eg, changes to eligibility criteria, outcomes, analyses) to relevant parties (eg, investigators, REC/IRBs, trial participants, trial registries, journals, regulators)	Ethics&Dissemination, p11-12;
Consent or assent	26a	Who will obtain informed consent or assent from potential trial participants or authorised surrogates, and how (see Item 32)	Study outline, p5
	26b	Additional consent provisions for collection and use of participant data and biological specimens in ancillary studies, if applicable	N/A
Confidentiality	27	How personal information about potential and enrolled participants will be collected, shared, and maintained in order to protect confidentiality before, during, and after the trial	Data management p10-11
Declaration of interests	28	Financial and other competing interests for principal investigators for the overall trial and each study site	Footnotes, p13
Access to data	29	Statement of who will have access to the final trial dataset, and disclosure of contractual agreements that limit such access for investigators	Ethics&Dissemination, p11-12; Footnotes, 13
Ancillary and post-trial care	30	Provisions, if any, for ancillary and post-trial care, and for compensation to those who suffer harm from trial participation	Concomitant care…, p8
Dissemination policy	31a	Plans for investigators and sponsor to communicate trial results to participants, healthcare professionals, the public, and other relevant groups (eg, via publication, reporting in results databases, or other data sharing arrangements), including any publication restrictions	Ethics&Dissemination, p11-12;
	31b	Authorship eligibility guidelines and any intended use of professional writers	Ethics&Dissemination, p11-12;

For peer review

	31c	Plans, if any, for granting public access to the full protocol, participant-level dataset, and statistical code	Ethics&Dissemination, p11-12;
**Appendices**			
Informed consent materials	32	Model consent form and other related documentation given to participants and authorised surrogates	Supplementary file S2 and S3
Biological specimens	33	Plans for collection, laboratory evaluation, and storage of biological specimens **for genetic or molecular analysis** in the current trial and for future use in ancillary studies, if applicable	N/A

*It is strongly recommended that this checklist be read in conjunction with the SPIRIT 2013 Explanation & Elaboration for important clarification on the items. Amendments to the protocol should be tracked and dated. The SPIRIT checklist is copyrighted by the SPIRIT Group under the Creative Commons "Attribution-NonCommercial-NoDerivs 3.0 Unported" license.

For peer review only

Amsterdam UMC
Universitair Medische Centra

INFORMATIEBRIEF VOOR PROEFPERSONEN

GALANT: Het effect van lanreotide op de grootte van niet-functionerende hypofyse macroadenomen (NFMA)

Officiële titel: Een gerandomiseerde, placebo-gecontroleerde studie onder patiënten met **Ga**llium-68 DOTATATE PET/CT positieve, klinisch niet-functionerende hypofyse macroadenomen (NFMA) naar het effect van **Lan**reotide autosolution op **T**umorgrootte (**GALANT**)

Geachte heer/mevrouw,

Wij vragen u vriendelijk om mee te doen aan een medisch-wetenschappelijk onderzoek. Wij hebben u benaderd omdat u op dit moment onder behandeling bent bij de afdeling Endocrinologie van het AMC vanwege een adenoom (tumor) van de hypofyse. U beslist zelf of u wilt meedoen. Voordat u de beslissing neemt, is het belangrijk om meer te weten over het onderzoek. Lees deze informatiebrief daarom rustig door en bespreek het met partner, vrienden en/of familie. Bekijk ook de bijgevoegde brochure 'Medisch-wetenschappelijk onderzoek', daarin staat veel algemene informatie. Hebt u na het lezen van de informatie nog vragen? Dan kunt u terecht bij de arts-onderzoeker of de onafhankelijke arts. In **bijlage A**, pagina 7, vindt u de contactgegevens.

1. Wat is het doel van het onderzoek?

Het doel van dit onderzoek is het testen van het medicijn lanreotide als behandeling van bepaalde hypofyse tumoren. De hypofyse is een klein orgaan dat vlak onder de hersenen hangt, achter de neusbrug. De hypofyse heeft een centrale rol in de hormoonhuishouding van het lichaam. Een hypofyse adenoom is een goedaardige tumor of gezwel van de hypofyse. Als dit adenoom zelf geen hormonen produceert wordt dit "niet-functionerend" genoemd. Adenomen groter dan 1 centimeter worden macroadenomen genoemd. Door groei van het adenoom kan het gezonde deel van de hypofyse in verdrukking komen. Hierdoor kunnen hormonen uitvallen waarvoor aanvulling nodig is met hormoontherapie ("substitutie"). Als het adenoom te groot wordt, en daardoor op de oogzenuw drukt, is een operatie noodzakelijk. Helaas zien we dat hierna het adenoom toch vaak terug komt. Op dit moment is behandeling met een medicijn nog niet mogelijk. De Endocrinologie afdelingen van het AMC en het VU Medisch Centrum (VUmc) in Amsterdam zijn een gezamenlijk onderzoek gestart naar de werkzaamheid van het medicijn lanreotide bij patiënten met een niet-functionerend hypofyse macroadenoom. Wij hopen dat door dit medicijn het adenoom langzamer groeit of zelfs slinkt. Dit zou betekenen dat een operatie niet of pas later nodig is. Het onderzoek wordt verricht met financiële steun van Ipsen, het farmaceutische bedrijf dat lanreotide produceert.

2. Wat is lanreotide precies?

Lanreotide is een eiwit dat lijkt op het natuurlijke hormoon somatostatine. Het behoort tot de groep "antigroei hormonen". De merknaam van lanreotide is Somatuline AutoSolution. Dit zijn twee namen voor hetzelfde medicijn. Het wordt toegediend als een injectie. Lanreotide wordt al gebruikt voor de behandeling van hypofyse adenomen die groeihormoon of schildklier stimulerend hormoon aanmaken. Uit een aantal onderzoeken bleek dat ongeveer een derde van de niet-functionerende hypofyse adenomen ook gevoelig kan zijn voor lanreotide, zelfs als ze geen hormoon aanmaken. Deze onderzoeken waren echter

For peer review only - http://bmjopen.bmj.com/site/about/guidelines.xhtml

Amsterdam UMC
Universitair Medische Centra

GALANT studie
NL52821.018.15

klein en er was geen vergelijking met een controlegroep. Hierdoor zijn de uitkomsten niet betrouwbaar genoeg.

3. Hoe wordt het onderzoek uitgevoerd?

*Zie ook het overzicht in **bijlage B***

Soort onderzoek

Het is een gerandomiseerd, dubbelblind, placebo-gecontroleerd onderzoek. Dit betekent dat een loting bepaalt in welke behandelgroep u komt (randomisatie). De ene groep krijgt het medicijn lanreotide en de andere groep krijgt een placebo (een "nepbehandeling") in de vorm van een onschadelijke zoutoplossing. Beide middelen worden als injectie toegediend. Dubbelblind betekent dat u en ook de onderzoekers niet weten welke behandeling u krijgt. Er vindt eerst een screening plaats om te zien of u mee kunt doen aan de loting en de behandeling.

De eerste bezoeken: screening

Indien u interesse heeft om mee te doen aan het onderzoek volgt een uitnodiging voor het eerste bezoek in het AMC. Hier zullen we eerst bekijken of deelname mogelijk is. Allereerst zal de onderzoeker wat algemene informatie vastleggen, zoals doorgemaakte en huidige ziektes, medicatiegebruik, allergieën etc. Wij vragen u ook een vragenlijst in te vullen over uw kwaliteit van leven. Hiernaast vinden tijdens het eerste bezoek een kort lichamelijk onderzoek en bloedafname (8 buisjes, 36 milliliter bloed) plaats. De MRI-scan van de hersenen wordt herhaald als het nodig is om de precieze grootte van het adenoom opnieuw te meten. Alleen adenomen groter dan 1 centimeter kunnen onderzocht worden. De genoemde testen en controles maken deel uit van uw reguliere behandeling, met uitzondering van de vragenlijst.

Als blijkt dat deelname mogelijk is volgt er nog een tweede screening. Deze tweede screening bestaat uit een speciale PET/CT scan. Deze scan laat zien of het adenoom bepaalde eigenschappen heeft waardoor lanreotide aan het adenoom kan binden. Zo ja, dan bestaat de kans dat het adenoom reageert op een behandeling met lanreotide. Alleen in dat geval kunt u meedoen aan het onderzoek. De scan vindt plaats in het AMC. Als dat mogelijk is al op dezelfde dag, maar hier kan ook een tweede bezoek voor nodig zijn. Voor de scan krijgt u een kleine hoeveelheid van een licht radioactieve stof (een radionuclide) toegediend via een infuus. Meer informatie over de PET/CT scan vindt u in **bijlage D**.

De verdere uitvoering en duur van het onderzoek

De loting vindt plaats als na de screening blijkt dat u mee kunt doen aan het onderzoek. Hierna starten de injecties. Elke 4 weken (28 dagen) krijgt u een injectie. Deze wordt diep onder de huid van de bil toegediend. In totaal krijgt u 18 injecties van óf lanreotide óf placebo. In principe worden de injecties elke 4 weken in het AMC toegediend door een gespecialiseerde endocrinologie verpleegkundige. Als dit voor u ver reizen is of als het niet goed uitkomt om overdag naar het AMC te komen, dan kunnen de injecties ook thuis of zelfs op werk toegediend worden. Dit wordt gedaan door verpleegkundigen van de Somacare thuiszorgservice van Eurocept Homecare. U wordt als deelnemer aan deze studie bij hen aangemeld zodat zij uw gegevens hebben en contact kunnen opnemen over de toediening.

Tijdens het onderzoek komt u verder elk halfjaar naar het AMC voor een controle-bezoek aan de arts-onderzoeker, in totaal drie keer. Hier herhalen we het lichamelijk onderzoek, de bloedafname en de vragenlijst. Ook vragen we naar mogelijke bijwerkingen of andere problemen. Het eerste en tweede controle-bezoek vinden plaats op de dag van een injectie, zodat deze tegelijk in het AMC kan plaatsvinden. Het derde en laatste controle-bezoek vindt plaats ná de laatste injectie. Rond het eerste en rond het laatste

For peer review only - http://bmjopen.bmj.com/site/about/guidelines.xhtml

bezoek wordt de MRI-scan van de hersenen herhaald om de grootte van het adenoom te bekijken. De totale duur van het onderzoek is ongeveer anderhalf jaar (18 maanden). In totaal zijn er minimaal 4 en maximaal 21 bezoeken aan het ziekenhuis. Dit hangt af van waar de injecties toegediend worden. In **bijlage B** vindt u een handig overzicht van het onderzoek.

4. Wat wordt er van u verwacht?

Indien u meedoet aan het onderzoek vragen wij u vaker naar het ziekenhuis te komen dan gebruikelijk. Hoe vaak u precies moet komen hangt af van waar de injecties worden toegediend. Als u de injecties in het AMC laat toedienen dan zijn deze bezoeken van korte duur. Om het onderzoek goed te kunnen uitvoeren is het belangrijk dat u zich houdt aan de geplande afspraken in het ziekenhuis en thuis. U kunt contact opnemen met de arts-onderzoeker of met Eurocept Homecare als u een afspraak wilt wijzigen.
Het onderzoek heeft geen invloed op uw dagelijkse activiteiten of werk. Er zijn geen beperkingen wat eten en drinken betreft. Lanreotide kan soms effect hebben op andere medicijnen. Daarom is het belangrijk dat de onderzoeker voor de start van het onderzoek uw medicatie controleert. Als deelname mogelijk is mag u uw eigen medicatie blijven gebruiken. Het is wel belangrijk de onderzoeker in te lichten voordat u nieuwe medicijnen gaat gebruiken tijdens de studie.

Voor vrouwelijke deelnemers

Geeft u borstvoeding, bent u zwanger of van plan dit te worden? Dan kunt u niet meedoen aan dit onderzoek. Bent u in de vruchtbare leeftijd? Dan is het van belang om tijdens het onderzoek adequate voorbehoedsmiddelen (anticonceptie) te gebruiken. Er is namelijk nog te weinig bekend over de gevolgen van lanreotide op het ongeboren kind. Wordt u toch zwanger tijdens het onderzoek? Neem dan contact op met de onderzoeker of uw arts. De zwangerschap wordt dan extra gecontroleerd.

5. Heeft meedoen invloed op de reguliere behandeling die u krijgt?

Meedoen heeft geen invloed op de reguliere behandeling van uw niet-functionerende hypofyse adenoom. Deze bestaat gewoonlijk uit controle van klachten, de grootte van het adenoom en laboratoriumbepalingen; naast eventuele hormoontherapie om tekorten aan te vullen ("substitutie"). Deze controles en therapie vinden ook plaats tijdens het onderzoek. Bepaalde klachten kunnen passen bij groei van het adenoom. Indien deze optreden tijdens het onderzoek krijgt u, conform de reguliere behandeling, een extra MRI-scan van de hersenen. Als blijkt dat het adenoom geopereerd moet worden stopt het onderzoek. Dit heeft geen consequenties voor uw verdere behandeling.

6. Welke bijwerkingen en ongemakken kunt u verwachten?

Lanreotide

Zoals alle medicijnen kan ook lanreotide bijwerkingen veroorzaken, al heeft lang niet iedereen hier last van. Hieronder staan de meest voorkomende bijwerkingen. Deze komen bij ongeveer 10% (één op de tien) van de mensen voor:

- Maag-darmklachten zoals diarree, buikpijn en misselijkheid (vooral na de eerste injectie)
- Vorming van galstenen (meestal zonder klachten)
- Huidreacties op de injectieplaats zoals zwelling, pijn en jeuk

Andere bijwerkingen komen minder vaak voor of zijn zeldzaam. Ook bestaat er altijd een kans op nog onbekende bijwerkingen. Meer informatie over de bijwerkingen vindt u in **bijlage C**. Daarin staat ook met wie u contact moet opnemen bij een mogelijk ernstige bijwerking.

For peer review only - http://bmjopen.bmj.com/site/about/guidelines.xhtml
For peer review only

MRI scans (reguliere behandeling)
Een MRI-scan werkt met een magneet, hierbij wordt u niet blootgesteld aan straling. Een MRI-scan kan niet plaatsvinden als zich in of op het lichaam kleine metaaldeeltjes bevinden, zoals een metaalsplinter in het oog, implantaten in de oren of metalen vaatclips in het hoofd. Ook kan dit niet bij geïmplanteerde elektronische apparatuur, zoals een pacemaker.

Bij de MRI-scans krijgt u een contrastmiddel toegediend via een bloedvat in de arm. Dit middel bevat geen jodium. In sommige gevallen ontstaan door het contrastmiddel kortdurend onschuldige bijwerkingen zoals hoofdpijn en misselijkheid. Als u bij een eerdere MRI-scan een allergische reactie hebt gehad kunt u niet meedoen met het onderzoek.

PET/CT scan en de radionuclide
Voor de PET/CT krijgt u zoals we eerder schreven een radionuclide toegediend, deze heet “Gallium-68 DOTATATE”. (Allergische) reacties hierop zijn zeldzaam. Het prikken van het infuus voor de toediening kan onprettig zijn en een blauwe plek veroorzaken.

De radionuclide is licht radioactief. Het stofje blijft net lang genoeg radioactief om de afbeeldingen te kunnen maken. Hierbij wordt u blootgesteld aan een kleine hoeveelheid straling. De geschatte stralingsbelasting van dit onderzoek is 3.1 mSv (millisievert). Uitgebreide informatie hierover vindt u in **bijlage D**.

7. Wat zijn mogelijke voor- en nadelen van deelname aan dit onderzoek?
Door resultaten uit eerder onderzoek is de verwachting dat lanreotide de groei van bepaalde hypofyse adenomen remt, maar zeker is dat niet. Het is daarom niet te zeggen of u voordeel heeft van deelname aan het onderzoek. Het huidige onderzoek zal het effect van lanreotide nauwkeuriger bestuderen en het vergelijken met een controlegroep. De kans om deel van die controlegroep te zijn en placebo injecties te krijgen is 50% (één op twee). Dit is een nadeel, ook vanwege de vele afspraken thuis of bezoeken aan het ziekenhuis om de injecties te ontvangen. Andere nadelen zijn de extra PET/CT scan met de bijbehorende straling en de mogelijke bijwerkingen of ongemakken van de injecties zelf. Tijdens de onderzoeksperiode wordt 4 keer een kleine hoeveelheid bloed afgenomen (telkens 36 milliliter). Dit gebeurt gewoonlijk ook tijdens de reguliere behandeling. Als er onverwacht afwijkingen uit het bloedonderzoek of de scans komen, zullen we dit altijd met u bespreken. Als u dit niet wilt, kunt u niet aan het onderzoek meedoen.

8. Wat gebeurt er als u niet wenst deel te nemen aan dit onderzoek of wilt stoppen?
Deelname aan het onderzoek is geheel vrijwillig. Als u besluit niet mee te doen, hoeft u niet te zeggen waarom. Als u wel meedoet, kunt u zich altijd bedenken en toch stoppen, ook tijdens het onderzoek. Als u besluit niet mee te doen of te stoppen, dan worden uw eigen behandeling en controles gewoon voortgezet. Tussentijds stoppen heeft geen risico’s voor uw gezondheid. Deelname kan ook door de onderzoeker worden beëindigd. Bijvoorbeeld als uw veiligheid in gevaar is.

Als er tijdens het onderzoek nieuwe informatie naar voren komt die belangrijk is voor u, laat de onderzoeker dit altijd aan u weten. U mag dan zelf beslissen of u blijft meedoen. Extra informatie over deelname, stoppen en uw rechten als proefpersoon kunt u vinden in de bijgevoegde brochure.

For peer review only - http://bmjopen.bmj.com/site/about/guidelines.xhtml

9. Wat gebeurt er met uw gegevens?

Privacy

Voor dit onderzoek worden uw medische en persoonlijke gegevens op verschillende manieren verzameld en gebruikt. Al uw gegevens blijven vertrouwelijk.

Hieronder staat waar en waarom uw persoonsgegevens verzameld worden:

- Bij de thuiszorgservice van Eurocept Homecare worden uw persoonsgegevens geregistreerd voor het inplannen en toedienen van injecties thuis.
- In een apart studiedossier in het AMC slaat de arts-onderzoeker uw contactgegevens op zodat zij u kan bereiken als dat nodig is.

Hiernaast worden alle gegevens voor het onderzoek verzameld en opgeslagen met behulp van een studiecode. Elke proefpersoon krijgt een eigen studiecode die de persoonsgegevens zoals uw naam vervangt. Welke code u heeft is alleen bekend bij het onderzoeksteam. Bij bespreken van en in rapporten over het onderzoek wordt alleen de studiecode gebruikt.

Inzien van onderzoeksgegevens

Om te controleren of het onderzoek degelijk en betrouwbaar wordt uitgevoerd, mogen bepaalde mensen wel uw gegevens zonder code inzien. Naast het onderzoeksteam zijn dit een controleur van het AMC en de Inspectie voor de Gezondheidszorg. Zij zijn verplicht om uw gegevens geheim te houden. Als vertegenwoordigers van Ipsen, de financier van het onderzoek, het onderzoek moeten controleren dan mogen zij de gegevens alleen met de studiecode inzien.

Bewaren van gegevens

Alle onderzoeksgegevens moeten wettelijk 15 jaar bewaard worden. Bij het ondertekenen van het toestemmingsformulier geeft u toestemming voor het verzamelen, inzien en bewaren van uw medische en persoonsgegevens. Algemene informatie over omgang met gegevens vindt u ook in de bijgevoegde brochure.

Bewaren van bloed

Gedurende de onderzoeksperiode wordt als reguliere controle een paar keer bloed afgenomen. Een deel hiervan word langer bewaard dan normaal, ook met gebruik van de code. Dit gebeurt voor het geval er later aanvullende bepalingen nodig blijken. Het bewaarde bloed wordt alleen voor het huidige onderzoek gebruikt. Het wordt vernietigd zodra het onderzoek in zijn geheel is voltooid. Dit houdt in dat alle patiënten die meedoen het onderzoek hebben afgerond en dat de resultaten kunnen worden gepubliceerd. In totaal zal het niet langer dan 5 jaar bewaard worden. Voor het bewaren van uw bloed geeft u toestemming via het toestemmingsformulier.

10. Bent u verzekerd wanneer u aan het onderzoek meedoet?

Voor iedereen die meedoet aan dit onderzoek is een verzekering afgesloten. De verzekering dekt schade als gevolg van het onderzoek. Dit geldt voor schade die naar boven komt tijdens het onderzoek, of binnen vier jaar na het einde van het onderzoek. In **bijlage E** vindt u de verzekerde bedragen, de uitzonderingen en de contactgegevens van de verzekeraar.

For peer review only - http://bmjopen.bmj.com/site/about/guidelines.xhtml

11. Wordt uw huisarts en/of behandelend specialist geïnformeerd bij deelname?

Wij laten uw huisarts en uw behandelend internist-endocrinoloog altijd schriftelijk weten dat u meedoet aan het onderzoek. Hiervoor geeft u toestemming via het toestemmingsformulier. Als u geen toestemming geeft, is meedoen niet mogelijk.

12. Is er een vergoeding wanneer u besluit deel te nemen?

De studiemedicatie, extra bezoeken en PET/CT scan kosten u niks. U wordt niet betaald voor het meedoen aan dit onderzoek. Wel krijgt u een vergoeding voor uw reiskosten.

13. Welke medisch-ethische toetsingscommissie heeft dit onderzoek goedgekeurd?

De medisch-ethische toetsingscommissie (METC) van het AMC heeft dit onderzoek goedgekeurd. Meer informatie over goedkeuring vindt u in de algemene brochure.

14. Wilt u verder nog iets weten?

U hoeft niet direct te beslissen of u mee wilt doen. De bedenktijd is ongeveer een week. Bij vragen kunt u contact opnemen met de arts-onderzoeker, Tessel Boertien. Voor onafhankelijk advies over meedoen aan dit onderzoek kunt u terecht bij de onafhankelijke arts, dr. M.R. Soeters, internist-endocrinoloog. Hij weet van het onderzoek af maar is zelf niet betrokken. Naast de algemene brochure kunt u ook op de website van het AMC veel informatie vinden, onder andere over het meedoen aan onderzoek en rechten & plichten als patiënt. Alle contactgegevens vindt u in **bijlage A**.

Dank voor uw aandacht en met vriendelijke groet,

Drs. Tessel M. Boertien, arts-onderzoeker en projectleider
Prof. Dr. E. Fliers, internist-endocrinoloog en hoofdonderzoeker AMC

Bijlagen bij deze informatiebrief:

- Los: Brochure 'Medisch-wetenschappelijk onderzoek. Algemene informatie voor de proefpersoon' (versie september 2014)
- Bijlage A: Contactgegevens en informatie per centrum
- Bijlage B: Overzicht van het onderzoek
- Bijlage C: Bijwerkingen lanreotide – Somatuline AutoSolution
- Bijlage D: Extra informatie over PET/CT en stralenbelasting
- Bijlage E: Verzekeringstekst
- Bijlage F: Toestemmingsformulier

For peer review only

For peer review only - http://bmjopen.bmj.com/site/about/guidelines.xhtml

Bijlage A: Contactgegevens en informatie per centrum

Projectleider & eerste contactpersoon

Drs. Tessel M. Boertien, arts-onderzoeker endocrinologie AMC & VUmc
e-mail **t.m.boertien@amsterdamumc.nl**
Tel. **020 566 0172** / 020 566 6071 (secr.)

Postadres onderzoek: Academisch Medisch Centrum, t.a.v. T.M. Boertien
Afdeling Endocrinologie & Metabolisme, kamer K2-283
Meibergdreef 9, 1105 AZ Amsterdam

Onafhankelijk arts AMC

Dr. M.R. Soeters, internist-endocrinoloog
e-mail m.r.soeters@amsterdamumc.nl
Tel. 020 566 6071

Informatie Academisch Medisch Centrum (AMC)

Bezoekadres: Meibergdreef 9
1105 AZ Amsterdam
Algemeen nummer: 020 566 9111
Nucleaire geneeskunde: 020 566 2775 (7:30-17:00)

Bij acute problemen buiten kantoortijden kunt u via het algemene telefoonnummer (020 566 9111) 24 uur per dag worden doorverbonden naar de dienstdoende internist-endocrinoloog. Vermeld altijd dat u meedoet aan deze studie.

Hoofdonderzoeker

Prof. dr. E. Fliers, internist-endocrinoloog
e-mail e.fliers@amsterdamumc.nl
Tel. 020 566 6071

Endocrinologie verpleegkundigen

Moniek Gent-Houben en Shariefa Peters-Amanatkhan
e-mail fk.endocrinologie@amsterdamumc.nl
Tel. 020 566 2990

Afdeling Patiëntenvoorlichting en Klachtenopvang AMC

Bij de afdeling patiëntenvoorlichting en klachtenopvang in het AMC kunt u terecht met algemene vragen en informatie, bijvoorbeeld over het meedoen aan onderzoek. Ook kunt u hier terecht indien u ontevreden bent over de gang van zaken of een klacht wilt indienen. De medewerkers verzorgen de eerste klachtenopvang en informeren over de klachtenprocedure. Op de website van het AMC is veel van de informatie terug te vinden: https://www.amc.nl/web/ik-heb-een-afspraak-1/rechten-en-plichten.htm

Locatie binnen AMC: begane grond van het polikliniekgebouw (A0)
Openingstijden: maandag t/m vrijdag van 9:00 tot 12:30 uur

For peer review only - http://bmjopen.bmj.com/site/about/guidelines.xhtml

Tel.:	020 566 3355
e-mail:	patientenvoorlichting@amc.nl
	klachtenfunctionaris@amc.nl
Postadres:	Academisch Medisch Centrum, t.a.v. patiëntenvoorlichting, A0-144
	Postbus 22660, 1100 DD Amsterdam

Contactgegevens Eurocept Homecare

Tel.: 035 528 8375 (ook buiten kantooruren)

e-mail galantstudie@eurocept.nl

For peer review only

For peer review only - http://bmjopen.bmj.com/site/about/guidelines.xhtml

GALANT studie
NL52821.018.15

Amsterdam UMC
Universitair Medische Centra

BIJLAGE B: OVERZICHT VAN HET ONDERZOEK

Hieronder staat een schematisch overzicht van het onderzoek. De studie bezoeken met de **dikgedrukte** nummers vinden in ieder geval plaats. In dit voorbeeld overzicht staat de PET/CT scan gepland tijdens een apart bezoek (nr. 2). Het kan zijn dat deze scan al tijdens bezoek nr. 1 plaatsvindt. De 7e en 13e injectie vinden plaats op de dag dat u ook naar het AMC komt. Het totale aantal studie bezoeken hangt af van waar de overige injecties plaatsvinden: thuis of in het AMC. Daarom staan die bezoeknummers tussen haakjes. Als alle injecties in het AMC plaatsvinden zijn er in totaal 21 bezoeken.

LOTING ↓

Tijd → Studie bezoek →	start **1**	 **2**	 (3 – 8)	6 mnd. **3** (9)	 (10 – 14)	12 mnd. **4** (15)	 (16 – 20)	18 mnd. **5** (21)	EINDE
Locatie →	AMC	AMC	AMC/thuis	AMC	AMC/thuis	AMC	AMC/thuis	AMC	
Duur in minuten →	90	90	15	90	15	60	15	75	

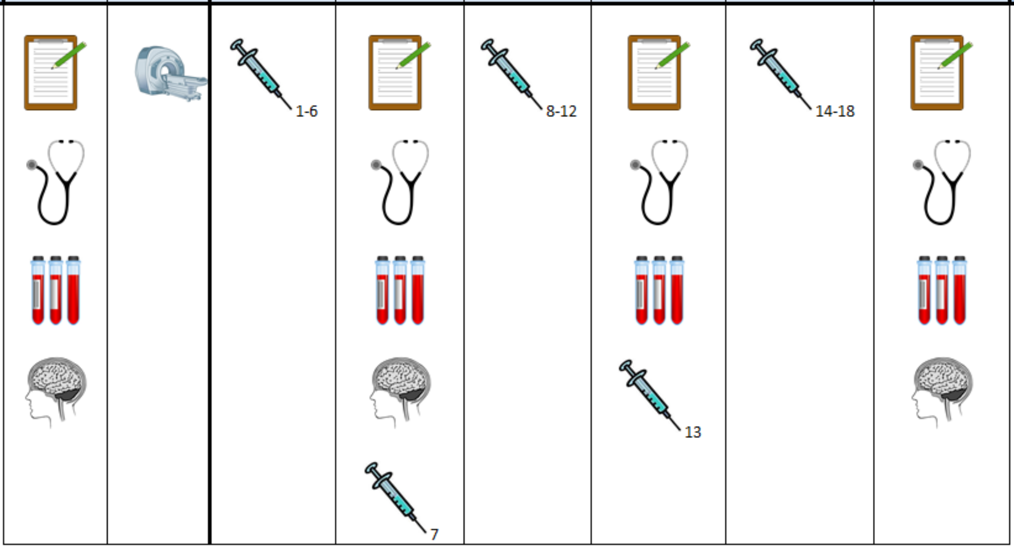

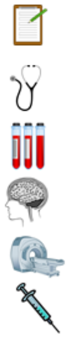

= gesprek en kwaliteit van leven vragenlijst

= kort lichamelijk onderzoek (bloeddruk, pols, lengte, gewicht; uitgebreider op geleide van klachten)

= bloed afname (één prik, 8 buisjes, 36 milliliter)

= MRI-hersenen

= PET/CT scan

= subcutane injectie (lanreotide / Somatuline AutoSolution óf placebo)

For peer review only - http://bmjopen.bmj.com/site/about/guidelines.xhtml

BIJLAGE C: BIJWERKINGEN LANREOTIDE – SOMATULINE AUTOSOLUTION

Zoals alle geneesmiddelen kan Somatuline AutoSolution, de merknaam van lanreotide, bijwerkingen veroorzaken. Lang niet iedereen krijgt hier last van.

De meest voorkomende bijwerkingen zijn zoals eerder genoemd maag-darmklachten, galblaasproblemen en reacties op de injectieplaats. Hieronder staat een lijst van de bijwerkingen die het vaakst optreden met Somatuline AutoSolution:

<u>Zeer vaak: komen voor bij meer dan 1 op de 10 gebruikers (>10%)</u>
- Diarree of dunne ontlasting, buikpijn
- Vorming van galstenen; dit veroorzaakt meestal geen klachten. Soms kan een galsteen echter leiden tot een verstopping van de galwegen. Dit kan de volgende klachten veroorzaken: ernstige en plotselinge buikpijn (vaak rechtsboven in de buik), hoge koorts, koude rillingen, geelzucht (het geel worden van de huid en het oogwit), verlies van eetlust, een jeukende huid

<u>Vaak: komen voor bij maximaal 1 op de 10 gebruikers (1-10%)</u>
- Reacties op de injectieplaats zoals zwelling, pijn en jeuk
- Misselijkheid, braken, constipatie (verstopping), winderigheid, opgezette buik, vreemd of opgeblazen gevoel in de buik, dyspepsie (gestoorde spijsvertering/maagklachten), vettige ontlasting
- Verminderde eetlust
- Gewichtsverlies
- Duizeligheid, hoofdpijn
- Pijn in de spieren, gewrichtsbanden, pezen of botten
- Vermoeidheid, gebrek aan energie of slaperigheid
- Trage hartslag
- Haaruitval of minder lichaamsbeharing
- Verwijding van de galwegen
- Verlaagde bloedsuikerspiegel: dit kan klachten geven van overmatig zweten, trillen, hongergevoel en verwardheid
- Verhoogde bloedsuikerspiegel: dit kan klachten geven van dorst, droge mond en vermoeidheid
- Afwijkende lever- en alveesklierwaarden, zoals blijkt uit bloedtesten

<u>Soms: komen voor bij maximaal 1 op de 100 gebruikers (<1%)</u>
- Opvliegers
- Slaapproblemen
- Verandering van de kleur van de ontlasting
- Veranderingen in de concentratie natrium en alkalinefosfaat, zoals blijkt uit bloedtesten

Wanneer u een ernstige bijwerking vermoedt of als u een andere ernstige klacht krijgt die hier niet tussen staat, raadpleeg dan de arts-onderzoeker! Indien geen gehoor of buiten kantooruren, raadpleeg dan de dienstdoende internist(-endocrinoloog) van uw ziekenhuis - *zie **bijlage A** voor contactgegevens*

For peer review only

For peer review only - http://bmjopen.bmj.com/site/about/guidelines.xhtml

BIJLAGE D: EXTRA INFORMATIE OVER PET/CT

Algemene informatie

De PET/CT scan wordt in het AMC uitgevoerd op de afdeling Nucleaire Geneeskunde. Deze vindt óf in de middag van het eerste bezoek plaats óf op een apart, tweede bezoek. U hoeft voor de scan niet nuchter te zijn. Op de afdeling Nucleaire Geneeskunde krijgt u een infuusnaald geplaatst in één arm. Via dit infuus krijgt u een kleine hoeveelheid van de licht radioactieve stof toegediend (de radionuclide Gallium-68 DOTATATE). Hierna wordt het infuus verwijderd. Het duurt 45 tot 60 minuten voordat deze stof goed is opgenomen door het lichaam en de hypofyse. Gedurende deze wachttijd mag u in de wachtkamer plaatsnemen, of iets gaan eten of drinken. Hierna neemt u rustig plaats op het bed van de PET/CT-scanner. Het maken van de scan duurt ongeveer 10 minuten, waarbij u op het bed door de brede ring van de scanner wordt geschoven. Deze ring is aan de voor en achterkant open. De laborant is constant aanwezig, en u kunt via de intercom contact met elkaar houden. Belangrijk is dat u tijdens het gehele onderzoek zo stil mogelijk blijft liggen. Na het maken van de scan mag u meteen naar huis. Het toedienen van de radionuclide heeft geen risico voor de omgeving. U mag na de scan meteen alles doen wat u normaal ook zou doen.

Stralingsbelasting

De stralingsbelasting wordt uitgedrukt in mSv (millisievert). De geschatte stralingsbelasting in dit onderzoek is 3.1 mSv. Ter vergelijking: de achtergrondstraling in Nederland is 2-2,5 mSv per jaar. Als u vaker meedoet aan wetenschappelijk onderzoek met stralingsbelasting, moet u er op letten dat de totale hoeveelheid over 5 jaar niet meer is dan 10 mSv. Ook kunt u beter niet mee doen aan dit onderzoek als u in uw beroep meer straling ontvangt dan de achtergrondstraling.

Wat is het risico van straling?

Het lichaam bestaat uit verschillende cellen. Wij hebben bijvoorbeeld hersencellen, spiercellen, bloedcellen, etc. Het zijn de genen (het erfelijke materiaal) in de cel die bepalen hoe de cel functioneert. Röntgenstraling of straling van radioactieve stoffen kan genen beschadigen. Deze beschadiging kan leiden tot kanker of een aangeboren afwijking.

Hoe groot zijn de kansen op deze ernstige gevolgen?

Een blootstelling aan 1 mSv straling geeft een kans van 1 op 17.000 op kanker. Bij een stralingsbelasting van 5 mSv is deze kans 1 op 3.400 en bij 10 mSv 1 op 1.700. Kanker wordt meestal pas 20 jaar of nog later ná de blootstelling aan de straling vastgesteld. De genoemde kansen moet u vergelijken met de kans op kanker van 25% (1 op 4) die elk mens heeft.

Als genen in zaadcellen of eicellen van de ouder beschadigd raken, bestaat het risico op een aangeboren afwijking van het kind. Een dosis van 1 mSv straling zal bij 1 op de 77.000 baby's (van een ouder die deze stralingsdosis kreeg) de oorzaak zijn van een ernstige aangeboren afwijking. Deze kans moet u vergelijken met de kans van 2,3% (23 op de 1000) op een aangeboren afwijking die elke baby heeft.

For peer review only

For peer review only - http://bmjopen.bmj.com/site/about/guidelines.xhtml

Amsterdam UMC
Universitair Medische Centra

BIJLAGE E: VERZEKERINGSTEKST

Overeenkomstig de Wet medisch-wetenschappelijk onderzoek met mensen heeft de AMC Medical Research B.V. voor medisch-wetenschappelijk onderzoek een verzekering afgesloten die door het onderzoek veroorzaakte schade door dood of letsel van de proefpersoon dekt. Dit betreft zowel schade die zich tijdens de duur van het onderzoek openbaart als schade die zich openbaart, en gemeld is, binnen vier jaar na beëindiging van de deelname aan het onderzoek.
De verzekering biedt een dekking van €450.000 per proefpersoon met een maximum van €3.500.000 voor het hele onderzoek en een maximum van €5.000.000 per jaar voor alle onderzoeken van dezelfde opdrachtgever.

<u>De verzekering biedt dekking</u>:
- voor schade ten gevolge van de verwezenlijking van de deelname aan het wetenschappelijk onderzoek verbonden risico's waarover men niet schriftelijk is ingelicht;
- voor schade ten gevolge van de verwezenlijking van de risico's waarover de deelnemer wel is ingelicht, maar die zich in ernstiger mate voordoet dan is voorzien;
- voor schade ten gevolge van de verwezenlijking van de risico's waarover de deelnemer wel is ingelicht, maar die zeer onwaarschijnlijk werd geacht.

<u>De verzekering biedt geen dekking</u>:
- voor schade die het gevolg is van het uitblijven van een vermindering van de gezondheidsproblemen van de proefpersoon, dan wel het gevolg is van de verdere verslechtering van de gezondheidsproblemen, indien de deelname aan het wetenschappelijk onderzoek plaatsvindt in het kader van de behandeling van deze gezondheidsproblemen;
- voor schade door aantasting van de gezondheid van de proefpersoon waarvan aannemelijk is dat deze zich ook zou hebben geopenbaard wanneer de proefpersoon niet aan het onderzoek had deelgenomen;
- voor schade ten gevolge van deelname aan medisch-wetenschappelijk onderzoek waarbij in de kring van beroepsgenoten gebruikelijke handelingen op het gebied van de geneeskunst met elkaar worden vergeleken en aannemelijk is dat de schade het gevolg is van de toegepaste handelingen;
- voor schade die zich bij een nakomeling van de proefpersoon openbaart als gevolg van een nadelige inwerking van het onderzoek op de proefpersoon of de nakomeling;
- voor schade die het gevolg is van het niet of niet volledig opvolgen van aanwijzingen en instructies door de proefpersoon, indien de proefpersoon daartoe althans in staat is.

De verzekering dekt uitsluitend de schade van natuurlijke personen. De dekking van specifieke schades en kosten is tot bepaalde bedragen beperkt. Om aanspraak te kunnen maken op schadevergoeding dient de proefpersoon in geval van vermeende schade als gevolg van het onderzoek dit te melden aan:

Naam verzekeraar:	Centramed B.A.
Postadres:	Postbus 7374<br>2701 AJ Zoetermeer
Telefoonnummer:	070 301 7070
E-mail:	info@centramed.nl
Polisnummer:	620.872.806

Hiernaast wordt de proefpersoon verzocht contact op te nemen met de hoofdonderzoeker van het onderzoek, prof. dr. E. Fliers (Tel. 020 566 6071).

For peer review only - http://bmjopen.bmj.com/site/about/guidelines.xhtml

BIJLAGE F: TOESTEMMINGSFORMULIER

GALANT: Het effect van lanreotide op de grootte van niet-functionerende hypofyse macroadenomen (NFMA)

ABR: NL52821.018.15 – protocol versie 3.0, oktober 2016

Officiële titel: Een gerandomiseerde, placebo-gecontroleerde studie onder patiënten met **Ga**llium-68 DOTATATE PET/CT positieve, klinisch niet-functionerende hypofyse macroadenomen (NFMA) naar het effect van **Lan**reotide autosolution op **T**umorgrootte (**GALANT**)

Ik heb de informatiebrief voor de proefpersoon gelezen. Ik kon aanvullende vragen stellen. Mijn vragen zijn genoeg beantwoord. Ik had genoeg tijd om te beslissen of ik meedoe.

Ik weet dat meedoen helemaal vrijwillig is. Ik weet dat ik op ieder moment kan beslissen om toch niet mee te doen. Daarvoor hoef ik geen reden te geven.

Ik weet dat onverwachte afwijkingen die tijdens het onderzoek naar voren komen altijd met mij besproken zullen worden.

Ik geef toestemming om mijn huisarts en behandelend internist-endocrinoloog te informeren dat ik meedoe aan dit onderzoek.

Ik weet dat mijn persoonsgegevens worden geregistreerd bij Eurocept Homecare als de injecties thuis plaatsvinden.

Ik weet dat mijn contactgegevens in een studiedossier in het AMC worden opgeslagen zodat de arts-onderzoeker met mij contact kan onderhouden.

Ik weet dat het onderzoeksteam, de controleur van het AMC en vertegenwoordigers van de Inspectie voor de Gezondheidszorg mijn onderzoeksgegevens zonder code kunnen inzien.

Ik geef toestemming om mijn gegevens te verzamelen en te gebruiken voor de doelen die in de informatiebrief staan.

Ik geef toestemming om mijn onderzoeksgegevens 15 jaar na afloop van dit onderzoek te bewaren.

Ik geef toestemming om het afgenomen bloed gedurende het onderzoek te bewaren tot publicatie van de resultaten (korter dan 5 jaar), waarna het wordt vernietigd.

Ik geef ☐ **wel**
☐ **geen**
toestemming om in de toekomst benaderd te worden voor een vervolgonderzoek.

For peer review only

For peer review only - http://bmjopen.bmj.com/site/about/guidelines.xhtml

Amsterdam UMC
Universitair Medische Centra

GALANT studie
NL52821.018.15

Ik wil meedoen aan dit onderzoek.

Naam proefpersoon: ……………………………………………………………………………

Handtekening: ………………………………………………………….. Datum: ………/………../………..

Ik verklaar hierbij dat ik deze proefpersoon volledig heb geïnformeerd over het genoemde onderzoek.

Als er tijdens het onderzoek informatie bekend wordt die de toestemming van de proefpersoon zou kunnen beïnvloeden, dan breng ik hem/haar daarvan tijdig op de hoogte.

Naam onderzoeker: ……………………………………………………………………………

Handtekening: ………………………………………………………….. Datum: ………/………../………..

Aanvullende informatie is gegeven door (indien van toepassing):

Naam: ……………………………………………………………………………………………..

Functie: …………………………………………………………………………………………..

Handtekening: ………………………………………………………….. Datum: ………/………../………..

De proefpersoon krijgt een volledige informatiebrief mee, samen met een kopie van het getekende toestemmingsformulier

For peer review only

Amsterdam UMC
Universitair Medische Centra

SUBJECT INFORMATION FOR PARTICIPATION IN MEDICAL RESEARCH

GALANT: The effect of lanreotide on non-functioning pituitary macroadenoma (NFMA) size

Official study title: A randomized placebo-controlled study in patients with a **Ga**llium-68 DOTATATE PET/CT positive, clinically non-functioning pituitary macroadenoma (NFMA) of the effect of **Lan**reotide autosolution on **T**umor (adenoma) size (**GALANT**)

Dear Sir/Madam,

You are kindly requested to take part in a medical-scientific study. We have contacted you because you receive treatment for an adenoma (tumor) of the pituitary gland at the department of Endocrinology of the Academic Medical Center (AMC). Participation is voluntary. Participation requires your written consent. Before you decide whether you want to participate in this study, you will be given an explanation about what the study involves. Please read this information carefully and discuss it with your partner, friends and/or family. General information about participating in a study can be found in the enclosed general brochure on medical research. If you have any questions you can contact the (coordinating) investigator or the independent expert for an explanation. Contact details can be found in **appendix A**, page 7.

1. What is the purpose of the study?

The purpose of this study is to investigate the effect of the medicine lanreotide in the treatment of certain pituitary adenomas. The pituitary gland, or hypophysis, is a small but important endocrine organ. It sits at the base of the brain and is about the size and shape of a bean. It has a central role in the regulation of various hormones. Pituitary adenomas are benign tumors within the pituitary gland. Some adenomas produce excess hormone. If this is not the case the adenoma is called “non-functioning”. If the size exceeds 1 centimeter, it is called a macroadenoma. The growing adenoma can cause pressure on the pituitary gland and disrupt its function. This requires treatment to replace the missing hormones (“substitution therapy”). The growing adenoma can also cause pressure on the optic nerve, which leads to vision disturbances. If this happens, a surgical operation is necessary. Sadly, however, the adenoma grows back in most cases. At this time, no medicinal treatment is available. The departments of Endocrinology of the AMC and the VU Medical Center (VUmc) in Amsterdam have therefore started a study to examine the effect of the medicine lanreotide on the size of non-functioning pituitary macroadenoma. We hope that this medicine can slow the growth of the adenoma or even decrease its size. This could prevent or postpone surgery. The study is realized with the financial support of Ipsen, the pharmaceutical company that produces lanreotide.

2. What exactly is lanreotide?

Lanreotide is a medicinal protein that is similar to the naturally occurring hormone somatostatin. This hormone blocks the release of several other hormones such as growth hormone. The brand name of lanreotide in the Netherlands is Somatuline AutoSolution. Both names are used for the same drug. It is administered through an injection. Lanreotide is already used in the treatment of pituitary adenomas that produce growth hormone or thyroid stimulating hormone. Earlier research has shown that about one-third of non-functioning adenomas might also be susceptible to lanreotide and could benefit from treatment.

For peer review only - http://bmjopen.bmj.com/site/about/guidelines.xhtml

However, these studies were small and lacked comparison with a control group. Therefore the results are not reliable enough.

3. What does participation involve?

*Also see the study overview in **appendix B***

Study design

This is a randomized, double-blind and placebo-controlled study. Randomization means that chance decides which patient gets which study treatment. There are two treatment groups. One group will receive the medicine lanreotide and the other group will receive a placebo or "sham treatment", in this case a harmless saline solution. Both treatments are administered as an injection. Every patient will also receive their regular care. Double-blind means that neither the patient nor the investigators know which patient is getting which treatment. First, a screening will take place to see if you can participate in the randomization and treatment.

Screening: the first visits

If you are interested in participating in the study you will be invited for the first visit at the AMC. We will first evaluate whether you may participate. The investigator will register some general information about your medical history, medication use, allergies etc. We will also request you to fill in a questionnaire about your health and daily activities. Furthermore the first visit will involve a short physical examination and blood tests (8 tubes, 36 milliliters). The MRI-scan of the brain will be repeated if it is necessary to measure the size of the adenoma again. Only adenomas larger than 1 centimeter can be examined in this study. Apart from the questionnaire, the tests are part of your regular care.

If the first screening is okay, a second screening will follow. This screening consists of a specific PET/CT scan. This scan shows if the adenoma has certain characteristics that could make it susceptible for lanreotide. If so, treatment with lanreotide might be effective. Only in that case participation in the study is possible. The PET/CT takes place at the AMC. If possible on the same day of the first screening, but a second visit could be necessary. Before the scan is performed, you will receive a small amount of a slightly radioactive marker (called a radionuclide) through an intravenous injection. You can find more information about this scan in **appendix D**.

The treatment, further visits and duration of the study

Randomization takes place when the screening shows that participation is possible. Then the treatment with the injections starts. Every 4 weeks (28 days) you will receive one injection, administered in the skin of the buttock ('subcutaneously'). In total you will receive 18 injections of either lanreotide or placebo. A specialized endocrine nurse will administer the injections at the AMC. If travelling to the AMC every 4 weeks is inconvenient for you, the injections can also be administered at home or even at work. Somacare nurses from Eurocept Homecare will perform this. They will register you as a participant in this study so they can contact you about the administration.

Every six months there will be a control visit to the investigator at the AMC, three times during the study. At these visits we will repeat the physical examination, blood tests and the questionnaire. We will also ask about possible side effects or other problems. The first and second control visit will take place on the day of a planned injection, so these can take place at the same time. The third and final control visit will take place after the last injection. Around the first and the last visit the MRI scan of the brain will be repeated to check the size of the adenoma. The duration of the study is about 18 months. In total there will be a minimum of

For peer review only - http://bmjopen.bmj.com/site/about/guidelines.xhtml

4 and a maximum of 21 visits to the hospital, depending on where the injections are administered. In **appendix B** a useful overview of the study is shown.

4. What is expected of you?

If you decide to participate in the study we will ask you to come to the hospital more often than usual. The amount of visits depends on where the injections are administered. If the injections are administered at the AMC these visits will be short. In order to carry out the study properly it is important to keep to the planned appointments in the hospital and at home. If you need to reschedule you can contact the investigator or Eurocept Homecare. Participation will have no influence on your daily activities. There are no restrictions on eating or drinking. Lanreotide can have some effect on other medicines. That is why it is important to check your medication use before the start of the study. If participation is possible you can continue all of your usual medication. We will discuss it with you if extra monitoring is necessary. It is important to contact the investigator before starting to use any new medicines during the study.

For female participants

If you are breastfeeding, pregnant, or planning to become pregnant, you cannot participate in this study. For fertile women it is important to use adequate contraception. This is because there is still not enough data on the effects of lanreotide on the unborn child. If you do, however, become pregnant during the study, please contact the investigator. The pregnancy will then be monitored more closely.

5. Will participation affect your regular treatment?

Participation will not affect your regular treatment of the pituitary adenoma. Regular treatment consists of monitoring complaints, regular blood tests and checking the size of the adenoma; next to the substitution of missing hormones. This treatment is continued during the study. Certain complaints can indicate increasing size of the adenoma. If this occurs during the study, you will undergo an extra MRI-scan. If the scan shows that surgery is needed to remove the adenoma, participation in the study will stop. Withdrawal from the study will not affect your regular treatment.

6. Can you expect any side effects or discomfort?

Lanreotide

Like all medicines lanreotide may have side effects. The most common side effects are listed below. These occur in about one in ten people (10%):

- Gastrointestinal complaints such as diarrhea, abdominal pain or nausea (especially after the first injection)
- Formation of gallstones (mostly without any symptoms)
- Skin reactions where the injection is given such as swelling, pain or itching

Other known side effects are less common or rare. The drug may also have side effects that are still unknown. Please see **appendix C** for a more extensive list of possible side effects and information on who to contact if any side effect becomes severe.

MRI scans (regular care)

An MRI scan is performed using a magnet, this doesn't involve any exposure to radiation. Because of the powerful magnet an MRI scan cannot be performed if there are small parts of metal in the body, such as

For peer review only - http://bmjopen.bmj.com/site/about/guidelines.xhtml

metal fragments in the eye or certain implants. The magnet can also interfere with implanted electronic devices such as a pacemaker.

To perform the MRI scan a contrast agent is administered through an intravenous injection. During the injection you may shortly experience harmless side effects like headache and nausea. If you had an allergic reaction during earlier MRI scans you cannot participate in this study.

PET/CT scan and the radionuclide

The earlier mentioned radionuclide used to perform the PET/CT is called "Gallium-68 DOTATATE". (Allergic) reactions to this radionuclide are rare. The injection to administer the radionuclide can be bothersome and may cause some bruising.

The radionuclide is slightly radioactive. The marker stays radioactive just long enough to perform the scan and generate the images. During this time you will be exposed to a small amount of radiation, estimated 3.1 mSv (millisievert). More extensive information on radiation exposure can be found in **appendix D**.

7. What are the possible advantages and disadvantages of participation?

The results of earlier research suggest that lanreotide may reduce the growth of non-functioning pituitary adenoma, but this is not certain. We can therefore not predict whether you will benefit from participation in this study. The current study will examine the effect of lanreotide more accurately and compare it with a control group. The chance to be part of the control group and receive placebo injections is 50% (one in two). This can be a disadvantage, also because of the frequent appointments at home or at the hospital to receive the injections. Further disadvantages are the additional PET/CT scan with the exposure to radiation and the possible discomfort and side effects of the injections. Blood tests are performed 4 times during the study period (each time 8 tubes and 36 milliliters). This is part of your regular care. If the blood tests or the scans lead to incidental findings about your health we will discuss these with you. If you do not want to be informed about these findings you cannot participate in the study.

8. What if you do not wish to participate or wish to stop participating?

It is up to you to decide whether or not to participate in the study. Participation is voluntary. If you do not want to participate, you will continue your usual treatment as before.
If you do participate in the study, you can always change your mind. You may stop participation at any time during the study. This is not harmful and will not affect your usual treatment. You do not have to say why you are stopping, but you do need to tell the investigator immediately. Participation may also be stopped by the investigator if it is considered best for you.

If there is any new information about the study that is important for you, the investigator will let you know. You will then be asked whether you still want to continue your participation. The data collected until that time can still be used for the study. Additional information about participation and your rights can be found in the general brochure.

9. How will your data be handled?

Privacy

For this study your medical and personal data will be collected and used in several ways. All your data will remain confidential.

For peer review only - http://bmjopen.bmj.com/site/about/guidelines.xhtml

Here we list where and why your personal data will be collected:

- At Eurocept Homecare your personal data will be registered in order to plan and administer injections at home.
- At the AMC the coordinating investigator will store your contact information so that she can reach you if necessary.

Data for the study will be collected and stored using a study code. Each participant will get a study code that replaces all personal data. Only the research team knows the code given to your data. In communication and reports about the study the study code will be used to protect your privacy.

Access to your data

To check whether the study is being performed according to the guidelines, some people may access the study data without using the code. Apart from the research team this concerns the monitor of the study and the Healthcare Inspectorate. They are obliged to keep your data secret. If representatives of Ipsen, the financier of the study and manufacturer of lanreotide, need to check the study, they only have access to the encoded data.

Storing of data

All data collected during this study needs to be stored for 15 years. If you sign the consent form, you consent to your medical and personal data being collected, stored and accessed. General information about the usage and storage of data can be found in the general brochure on medical research.

Storing of blood

During the study period blood tests are performed as part of your regular care. Part of the collected blood will be stored for a longer period than usual, also using the study code. We will store the blood in case we need to perform additional tests. The stored blood samples will only be used for this study. It will be destroyed once the study is concluded. The study is concluded once all the participants have completed the study and the results can be published. This will not be longer than 5 years. If you sign the consent form, you consent to the temporary storage of your blood samples.

10. Are you insured if you participate in this study?

Insurance has been taken out for everyone participating in this study. This insurance covers damage caused by the study. This applies to damage manifesting during the study or within four years of the end of the study. The insurance does not cover all damages. **Appendix E** contains more information about the insurance and the contact information.

11. Will my GP and/or my treating specialist be informed if I participate?

We will send your GP ("huisarts") and your treating internist-endocrinologist a letter to let them know that you are participating in the study. This is for your own safety. If you do not agree to this, you cannot participate in this study.

12. Is there a compensation when you participate?

The study medication, additional visits and PET/CT scan for the study are free of charge for you. You will not be paid for your participation in this study, but you will be reimbursed for your travel costs.

For peer review only - http://bmjopen.bmj.com/site/about/guidelines.xhtml

13. Which Ethics Committee has approved this study?

The Ethics Committee of the AMC has approved this study. General information about the assessment of research can be found in the general brochure on medical research.

14. Any questions?

You do not have to decide right away whether you want to participate in the study. In about a week we will get in touch with you to inform about your decision. If you have any questions or would like more information, please contact the coordinating investigator, Tessel Boertien. If you would like independent advice about participation in this study, you may contact the independent doctor M.R. Soeters, internist-endocrinologist. He knows about the study but is not involved in it. All contact details can be found in **appendix A**.

Thank you for your attention and kind regards,

T.M Boertien, MD, coordinating investigator and project leader
Prof. E. Fliers, MD PhD, internist-endocrinologist and principal investigator AMC

Appendices:

- Separate: Brochure 'Medical research. General information for research participants'
- Appendix A: Contact details and hospital information
- Appendix B: Study overview
- Appendix C: Side effects of lanreotide – Somatuline AutoSolution
- Appendix D: Additional information on PET/CT and radiation exposure
- Appendix E: Insurance information
- Appendix F: Informed Consent form

For peer review only

For peer review only - http://bmjopen.bmj.com/site/about/guidelines.xhtml

APPENDIX A: INFORMATION ON THE PARTICIPATING CENTERS & CONTACT DETAILS

Coordinating investigator & project leader
T.M. Boertien, MD
PhD candidate Endocrinology AMC & VUmc
email: **t.m.boertien@amsterdamumc.nl**
Tel. **020 566 3507** / 020 566 6071 (secr.)

Study postal address: Academisch Medisch Centrum, t.a.v. T.M. Boertien
Afdeling Endocrinologie & Metabolisme, K2-283
Meibergdreef 9, 1105 AZ Amsterdam

Independent expert
M.R. Soeters, MD PhD, internist-endocrinologist
email: m.r.soeters@amsterdamumc.nl
Tel. 020 566 6071

Information for Academic Medical Center (AMC)
Address: Meibergdreef 9
1105 AZ Amsterdam
General no.: 020 566 9111
Nuclear medicine: 020 566 2775 (7:30-17:00)

In case of acute problems outside office hours you can call the general number of the AMC (020 566 9111) 24 hours a day and ask to be put through to the attending internist-endocrinologist. Always mention your participation in this study.

Principal investigator
Prof. E. Fliers, MD PhD, internist-endocrinologist
email: e.fliers@amsterdamumc.nl
Tel. 020 566 6071

Endocrine nurses
Moniek Gent-Houben and Shariefa Peters-Amanatkhan
e-mail fk.endocrinologie@amsterdamumc.nl
Tel. 020 566 2990

AMC department for patient information and complaints
The AMC department for patient information and complaints is at your service for general questions and information about matters such as participating in medical-scientific research. If you are not satisfied or unhappy with the care, treatment or services, you can inform the staff about your complaint. They can inform you about the procedure of filing a complaint and if necessary call in the complaints officer.

Location within AMC: outpatients clinic at the ground floor, A0-144

For peer review only - http://bmjopen.bmj.com/site/about/guidelines.xhtml

GALANT study
NL52821.018.15

Opening hours:	Monday to Friday from 9:00 to 12:30 hrs
email:	patientenvoorlichting@amc.nl
Tel.:	020 566 3355
Postal address:	Academisch Medisch Centrum, t.a.v. patiëntenvoorlichting, A0-144<br>Postbus 22660, 1100 DD Amsterdam

Contact details Eurocept Homecare

Tel.:	035 528 8375 (24 hrs a day)
email	galantstudie@eurocept.nl

For peer review only

For peer review only - http://bmjopen.bmj.com/site/about/guidelines.xhtml

APPENDIX B: STUDY OVERVIEW

This is an overview of the study period. The study visit with **bold** numbers will definitely take place. In this example the PET/CT is planned during a separate visit (no. 2). It could be that this scan can already take place during visit no. 1. The 7th and the 13th injection will take place on a day that you also have a study visit at the AMC. The total amount of study visits depends on where the rest of the study injections are administered: at home or at the AMC. That is why those visit numbers are between parentheses ( ). If all injections take place at the AMC there will be 21 visits in total.

RANDOMIZATION ↓ (between visit 2 and visits 3 – 8)

Time point → / Study visit no. →	start **1**	**2**	(3 – 8)	6 mo. **3** (9)	(10 – 14)	12 mo. **4** (15)	(16 – 20)	18 mo. **5** (21)	END OF STUDY
Location →	AMC	AMC	AMC/home	AMC	AMC/home	AMC	AMC/home	AMC	
Duration in minutes →	90	90	15	90	15	60	15	75	

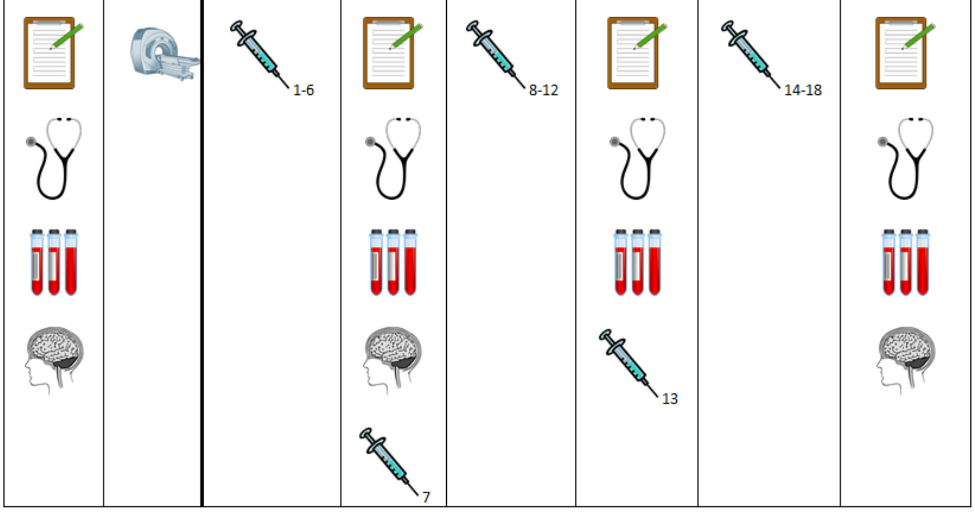

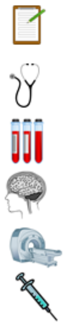

= interview and quality of life questionnaire

= short physical examination (blood pressure, heart rate, height, weight; if necessary more extensive)

= blood withdrawal (single puncture, 8 tubes, 36 milliliters)

= MRI scan of the brain

= PET/CT scan

= subcutaneous injection (lanreotide / Somatuline AutoSolution OR placebo)

For peer review only - http://bmjopen.bmj.com/site/about/guidelines.xhtml

Appendix C: Side effects of lanreotide – Somatuline AutoSolution

Like all medicines, Somatuline AutoSolution (the brand name of lanreotide) can cause side effects, although they do not affect everyone.

As mentioned earlier, the most commonly reported side effects are gastrointestinal complaints, gallbladder problems and injection site reactions. The list below shows the side effects that can occur with Somatuline AutoSolution according to their frequencies:

<u>Very common: may affect more than 1 in 10 people (>10%)</u>:
- Diarrhea, loose stools, abdominal pain
- Formation of gallstones; this normally doesn't cause symptoms. In some cases however a gallstone can lead to blockage of a bile duct. This can cause the following symptoms: severe and sudden abdominal pain, high fever, chills, jaundice (yellowing of the skin and whites of the eyes), loss of appetite, itchy skin

<u>Common: may affect up to 1 in 10 people (1-10%)</u>:
- Site reactions where the injection is given such as swelling, pain or itchiness
- Nausea, vomiting, constipation, gas formation/wind, stomach bloating or discomfort, indigestion, excess fat in stools
- Decrease in appetite
- Weight loss
- Feeling dizzy, headaches
- Pain that affects muscles, ligaments, tendons and bones
- Lack of energy or feeling tired
- Slow heart beat (sinus bradycardia)
- Loss of hair or less development of body hair
- Enlargement of the bile ducts between your liver and gallbladder and the intestine
- Lowered blood sugar level: this can cause symptoms of excessive sweating, feeling shaky, feeling hungry and confusion
- Heightened blood sugar level: this can cause symptoms of thirst, a dry mouth and feeling more tired than usual
- Abnormal liver and pancreas test results, shown in blood tests

<u>Uncommon: may affect up to 1 in 100 people (<1%)</u>:
- Hot flushes
- Difficulty sleeping
- A change in the color of the stools
- Changes to sodium and alkaline phosphatase levels, shown in blood tests

If any of these side effects becomes severe or if you experience a severe symptom that is not listed here, please contact the coordinating investigator or, if there is no answer or outside office hours, the attending internist-endocrinologist of your hospital - *see* ***appendix A*** *for contact details*

For peer review only - http://bmjopen.bmj.com/site/about/guidelines.xhtml

Appendix D: Additional information on PET/CT & radiation exposure

General information

The PET/CT scan will be performed at the department of Nuclear Medicine of the AMC. This will either take place in the afternoon of the first visit or during a separate, second visit. You are allowed to eat and drink before the scan. At the department of Nuclear Medicine, a small intravenous line will be placed in your arm. A small amount of the slightly radioactive marker (the radionuclide Gallium-68 DOTATATE) will be administered through the line. Following the injection the line will be removed. It takes 45 to 60 minutes for the radionuclide to be distributed through the body. During this time you can take place in the waiting room or go and have something to eat or drink. Then you will be placed comfortably on the PET/CT table and scanned. During the scan the table is moving through a large ring that is open on both sides. The technician is present continuously and you can contact him or her through the intercom. The imaging session will take about 10 minutes. It is important that you remain as still as possible. After the scan you may go home directly and continue your usual activities. The marker loses its radioactivity very quickly; it carries no risks for your environment.

Radiation exposure

The PET/CT involves using radioactive markers. Radiation exposure is expressed in mSv (millisievert). The total amount of radiation you will be exposed to in this study is estimated at 3.1 mSv. To compare: the background radiation in the Netherlands is 2-2.5 mSv per year. If you participate in scientific research involving exposure to radiation more often, you should discuss with the investigator whether participation now would be safe. The total amount of radiation due to scientific research should not exceed 10 mSv in 5 years. Participation is advised against if you are exposed to an amount of radiation that exceeds the background radiation as part of your job.

What is the risk of radiation?

The body is made up of different cells. There are brain cells, muscle cells, blood cells etc. The genes inside these cells carry the genetic material and determine the function of the cells. X-rays or radiation from radioactive substances can damage these genes. This can lead to cancer or a congenital (inborn) disorder.

What are the chances of these severe consequences?

Cancer due to radiation usually is not detected until 20 years after the exposure. An exposure of 1 mSv generates a chance of 1 in 17,000 to develop cancer. With an exposure of 5 mSv this chance is increased to 1 in 3,400 and with an exposure of 10 mSv to 1 in 1,700. You need to compare these chances with the chance of 25% (1 in 4) to develop cancer that every human has.

If genes in the reproductive cells (sperm and eggs) are damaged, there is a risk of a congenital (inborn) disorder of the child. An exposure of 1 mSv radiation of the parent will be the cause of a severe congenital disorder in 1 in 77,000 babies. You need to compare this chance with the chance of 2,3% (23 in 1,000) of a congenital disorder that every baby has.

For peer review only - http://bmjopen.bmj.com/site/about/guidelines.xhtml

APPENDIX E: INSURANCE INFORMATION

Insurance has been taken out by AMC Medical Research B.V. for everyone participating in this study. The insurance covers damage due to participation in the study. This applies to damage manifesting during the study or within four years of the end of the study. The insurance offers a cover of € 450,000 per study subject, with a maximum of € 3,500,000 for the entire study and € 5,000,000 annually for all studies from the same sponsor. You must notify the insurance company about the damage within those four years. This is set out in the Medical Research (Human Subjects) Compulsory Insurance Decree. This decree is listed on the website of the Central Committee on Research Involving Human Subjects www.ccmo.nl.

The insurance policy covers the following damage:

- damage as a result of a risk of participation in the clinical trial that you were not informed about in the written information;
- damage as a result of a risk that you were informed about in the written information, but which proved more serious than foreseen;
- damage as a result of a risk that you were informed about in the written information, but which was deemed very unlikely to occur.

The insurance policy does **not** cover the following damage:

- damage due to your health problems not being alleviated or your health problems deteriorating further, in case participation in the clinical trial took place within the context of treatment of those health problems;
- damage to your health that would also have occurred if you had not participated in the study;
- damage as a result of an existing treatment method for research into existing methods of treatment;
- damage to descendants as a result of a negative effect of the study on you or your descendants;
- damage resulting from not or not entirely following directions or instructions.

The insurance shall cover losses suffered by natural persons only. In the event of damage please contact the insurance company directly:

Name company:	Centramed B.A.
Postal address:	Postbus 7374<br>2701 AJ Zoetermeer
Telephone number:	070 301 7070
Email:	info@centramed.nl
Policy number:	620.872.806

Furthermore you are requested to contact the principal investigator of the study, prof. E. Fliers (tel. 020 566 6071).

For peer review only

For peer review only - http://bmjopen.bmj.com/site/about/guidelines.xhtml

Amsterdam UMC
Universitair Medische Centra

APPENDIX F: INFORMED CONSENT FORM

GALANT: The effect of lanreotide on non-functioning pituitary macroadenoma (NFMA) size

ABR: NL52821.018.15 – protocol version 3.0, 13 October 2016

Official study title: A randomized placebo-controlled study in patients with a **Ga**llium-68 DOTATATE PET/CT positive, clinically non-functioning pituitary macroadenoma (NFMA) of the effect of **Lan**reotide autosolution on **T**umor (adenoma) size (**GALANT**)

I have read the subject information letter. I was also able to ask questions. My questions have been answered to my satisfaction. I had enough time to decide whether to participate.

I know that participation is voluntary. I know that I may decide at any time not to participate after all or to withdraw from the study. I do not need to give a reason for this.

I know that incidental findings about my health during this study will be discussed with me.

I give permission to inform my GP and my treating specialist about my participation in this study.

I know that my personal data will be registered at Eurocept Homecare if the injections take place at home.

I know that my contact information will be stored in a study file at the AMC so that the coordinating investigator can contact me about the study.

I know that the research team, the study monitor and representatives of the Healthcare Inspectorate can access my data without the study code.

I consent to the collection and usage of my data for the purposes stated in the information letter.

I consent to my data being stored for another 15 years after this study.

I consent to my blood samples being stored during the study until publication of the results (maximum of 5 years), after which it shall be destroyed.

I ☐ **do**
☐ **do not**
consent to being contacted again after this study for a follow-up study.

For peer review only - http://bmjopen.bmj.com/site/about/guidelines.xhtml

Amsterdam UMC
Universitair Medische Centra

GALANT study
NL52821.018.15

I want to participate in this study.

Name study subject: ………………………………………………………………………………….

Signature: ……………………………………………………………………   Date: …………/…………/………….

I hereby declare that I have fully informed this study subject about this study.

If information comes to light during the course of the study that could affect the study subject's consent, I will inform him/her of this in a timely fashion.

Name investigator: …………………………………………………………………………………

Signature: ……………………………………………………………………   Date: …………/…………/………….

Additional information was given by (if applicable):

Name: ……………………………………………………………………………………….

Job title: ………………………………………………………………………………….

Signature: ……………………………………………………………………   Date: …………/…………/………….

The study subject receives a complete information letter and a copy of the signed informed consent form.

For peer review only - http://bmjopen.bmj.com/site/about/guidelines.xhtml

STANDARD PROTOCOL ITEMS: RECOMMENDATIONS FOR INTERVENTIONAL TRIALS

Manuscript Boertien et al. "The GALANT trial: study protocol of a randomized placebo-controlled trial in patients with a ^{68}Ga-DOTATATE PET/positive, clinically nonfunctioning pituitary macroadenoma on the effect of lanreotide on tumour size" – v 04-MAR-2020

SPIRIT 2013 Checklist: Recommended items to address in a clinical trial protocol and related documents*

Section/item	Item No	Description	Addressed in section, page no.
**Administrative information**			
Title	1	Descriptive title identifying the study design, population, interventions, and, if applicable, trial acronym	Title page, p1
Trial registration	2a	Trial identifier and registry name. If not yet registered, name of intended registry	Abstract, p2
	2b	All items from the World Health Organization Trial Registration Data Set	N/A (see registry)
Protocol version	3	Date and version identifier	Footnotes, p13
Funding	4	Sources and types of financial, material, and other support	Footnotes, p12-13
Roles and responsibilities	5a	Names, affiliations, and roles of protocol contributors	Title page, p1; Footnotes, p12
	5b	Name and contact information for the trial sponsor	Title page, p1
	5c	Role of study sponsor and funders, if any, in study design; collection, management, analysis, and interpretation of data; writing of the report; and the decision to submit the report for publication, including whether they will have ultimate authority over any of these activities	Ethics&Dissemination, p11-12; Footnotes, p12-13

For peer review only

	5d	Composition, roles, and responsibilities of the coordinating centre, steering committee, endpoint adjudication committee, data management team, and other individuals or groups overseeing the trial, if applicable (see Item 21a for data monitoring committee)	N/A
**Introduction**			
Background and rationale	6a	Description of research question and justification for undertaking the trial, including summary of relevant studies (published and unpublished) examining benefits and harms for each intervention	Introduction, p4
	6b	Explanation for choice of comparators	Intervention, p6
Objectives	7	Specific objectives or hypotheses	Introduction, p4
Trial design	8	Description of trial design including type of trial (eg, parallel group, crossover, factorial, single group), allocation ratio, and framework (eg, superiority, equivalence, noninferiority, exploratory)	Study design&setting, p5
**Methods: Participants, interventions, and outcomes**			
Study setting	9	Description of study settings (eg, community clinic, academic hospital) and list of countries where data will be collected. Reference to where list of study sites can be obtained	Study design&setting, p5
Eligibility criteria	10	Inclusion and exclusion criteria for participants. If applicable, eligibility criteria for study centres and individuals who will perform the interventions (eg, surgeons, psychotherapists)	Eligibility criteria, p5; Figure 2
Interventions	11a	Interventions for each group with sufficient detail to allow replication, including how and when they will be administered	Intervention p6; Allocation concealment…, p6-7
	11b	Criteria for discontinuing or modifying allocated interventions for a given trial participant (eg, drug dose change in response to harms, participant request, or improving/worsening disease)	Intervention, p6; Concomitant care…, p8
	11c	Strategies to improve adherence to intervention protocols, and any procedures for monitoring adherence (eg, drug tablet return, laboratory tests)	Intervention, p6
	11d	Relevant concomitant care and interventions that are permitted or prohibited during the trial	Concomitant care…, p8

For peer review only - http://bmjopen.bmj.com/site/about/guidelines.xhtml

Outcomes	12	Primary, secondary, and other outcomes, including the specific measurement variable (eg, systolic blood pressure), analysis metric (eg, change from baseline, final value, time to event), method of aggregation (eg, median, proportion), and time point for each outcome. Explanation of the clinical relevance of chosen efficacy and harm outcomes is strongly recommended	Primary outcome, p8; Secondary outcomes, p8-
Participant timeline	13	Time schedule of enrolment, interventions (including any run-ins and washouts), assessments, and visits for participants. A schematic diagram is highly recommended (see Figure)	Study outline, p5; Figure 3
Sample size	14	Estimated number of participants needed to achieve study objectives and how it was determined, including clinical and statistical assumptions supporting any sample size calculations	Sample size, p9
Recruitment	15	Strategies for achieving adequate participant enrolment to reach target sample size	Study outline, p5
**Methods: Assignment of interventions (for controlled trials)**			
Allocation:			
Sequence generation	16a	Method of generating the allocation sequence (eg, computer-generated random numbers), and list of any factors for stratification. To reduce predictability of a random sequence, details of any planned restriction (eg, blocking) should be provided in a separate document that is unavailable to those who enrol participants or assign interventions	Randomisation, p6
Allocation concealment mechanism	16b	Mechanism of implementing the allocation sequence (eg, central telephone; sequentially numbered, opaque, sealed envelopes), describing any steps to conceal the sequence until interventions are assigned	Randomisation, p6; Allocation concealment…, p6-7
Implementation	16c	Who will generate the allocation sequence, who will enrol participants, and who will assign participants to interventions	Allocation concealment…, p6-7
Blinding (masking)	17a	Who will be blinded after assignment to interventions (eg, trial participants, care providers, outcome assessors, data analysts), and how	Allocation concealment…, p6-7
	17b	If blinded, circumstances under which unblinding is permissible, and procedure for revealing a participant's allocated intervention during the trial	Allocation concealment…, p6-7
**Methods: Data collection, management, and analysis**			

Data collection methods	18a	Plans for assessment and collection of outcome, baseline, and other trial data, including any related processes to promote data quality (eg, duplicate measurements, training of assessors) and a description of study instruments (eg, questionnaires, laboratory tests) along with their reliability and validity, if known. **Reference to where data collection forms can be found, if not in the protocol**	Study outline, p5; Assessments…, p7; Outcomes, p8-9; Data management, p10-11
	18b	Plans to promote participant retention and complete follow-up, including list of any outcome data to be collected for participants who discontinue or deviate from intervention protocols	Study outline, p5; Statistical analyses, p9-10
Data management	19	Plans for data entry, coding, security, and storage, including any related processes to promote data quality (eg, double data entry; range checks for data values). **Reference to where details of data management procedures can be found, if not in the protocol**	Data management, p10-11
Statistical methods	20a	Statistical methods for analysing primary and secondary outcomes. Reference to where other details of the statistical analysis plan can be found, if not in the protocol	Statistical analyses, p9-10
	20b	Methods for any additional analyses (eg, subgroup and adjusted analyses)	Statistical analyses, p9-10
	20c	Definition of analysis population relating to protocol non-adherence (eg, as randomised analysis), and any statistical methods to handle missing data (eg, multiple imputation)	Statistical analyses, p9-10
**Methods: Monitoring**			
Data monitoring	21a	Composition of data monitoring committee (DMC); summary of its role and reporting structure; statement of whether it is independent from the sponsor and competing interests; and reference to where further details about its charter can be found, if not in the protocol. Alternatively, an explanation of why a DMC is not needed	N/A (no DMC), Monitoring, p11
	21b	Description of any interim analyses and stopping guidelines, including who will have access to these interim results and make the final decision to terminate the trial	N/A (no interim analyses)
Harms	22	Plans for collecting, assessing, reporting, and managing solicited and spontaneously reported adverse events and other unintended effects of trial interventions or trial conduct	Assessments.., p7; Safety, p11

For peer review only - http://bmjopen.bmj.com/site/about/guidelines.xhtml

Auditing	23	Frequency and procedures for auditing trial conduct, if any, and whether the process will be independent from investigators and the sponsor	Monitoring, p11
**Ethics and dissemination**			
Research ethics approval	24	Plans for seeking research ethics committee/institutional review board (REC/IRB) approval	Ethics&Dissemination, p11-12;
Protocol amendments	25	Plans for communicating important protocol modifications (eg, changes to eligibility criteria, outcomes, analyses) to relevant parties (eg, investigators, REC/IRBs, trial participants, trial registries, journals, regulators)	Ethics&Dissemination, p11-12;
Consent or assent	26a	Who will obtain informed consent or assent from potential trial participants or authorised surrogates, and how (see Item 32)	Study outline, p5
	26b	Additional consent provisions for collection and use of participant data and biological specimens in ancillary studies, if applicable	N/A
Confidentiality	27	How personal information about potential and enrolled participants will be collected, shared, and maintained in order to protect confidentiality before, during, and after the trial	Data management p10-11
Declaration of interests	28	Financial and other competing interests for principal investigators for the overall trial and each study site	Footnotes, p13
Access to data	29	Statement of who will have access to the final trial dataset, and disclosure of contractual agreements that limit such access for investigators	Ethics&Dissemination, p11-12; Footnotes, 13
Ancillary and post-trial care	30	Provisions, if any, for ancillary and post-trial care, and for compensation to those who suffer harm from trial participation	Concomitant care…, p8
Dissemination policy	31a	Plans for investigators and sponsor to communicate trial results to participants, healthcare professionals, the public, and other relevant groups (eg, via publication, reporting in results databases, or other data sharing arrangements), including any publication restrictions	Ethics&Dissemination, p11-12;
	31b	Authorship eligibility guidelines and any intended use of professional writers	Ethics&Dissemination, p11-12;

For peer review

	31c	Plans, if any, for granting public access to the full protocol, participant-level dataset, and statistical code	Ethics&Dissemination, p11-12;
**Appendices**			
Informed consent materials	32	Model consent form and other related documentation given to participants and authorised surrogates	Supplementary file S2 and S3
Biological specimens	33	Plans for collection, laboratory evaluation, and storage of biological specimens **for genetic or molecular analysis** in the current trial and for future use in ancillary studies, if applicable	N/A

*It is strongly recommended that this checklist be read in conjunction with the SPIRIT 2013 Explanation & Elaboration for important clarification on the items. Amendments to the protocol should be tracked and dated. The SPIRIT checklist is copyrighted by the SPIRIT Group under the Creative Commons "Attribution-NonCommercial-NoDerivs 3.0 Unported" license.

For peer review only

BMJ Open

The GALANT trial: study protocol of a randomised placebo-controlled trial in patients with a ^{68}*Ga*-DOTATATE PET-positive, clinically nonfunctioning pituitary macroadenoma on the effect of *lan*reotide on *t*umour size

Journal:	*BMJ Open*
Manuscript ID	bmjopen-2020-038250.R1
Article Type:	Protocol
Date Submitted by the Author:	26-Jun-2020
Complete List of Authors:	Boertien, Tessel; Amsterdam UMC - Locatie AMC, Department of Endocrinology and Metabolism<br>Drent, Madeleine; Amsterdam UMC - Locatie VUMC, Department of Internal Medicine, Section of Endocrinology<br>Booij, Jan; Amsterdam UMC - Locatie AMC, Department of Radiology and Nuclear Medicine<br>Majoie, Charles; Amsterdam UMC - Locatie AMC, Department of Radiology and Nuclear Medicine<br>Stokkel, Marcel; Netherlands Cancer Institute, Department of Nuclear Medicine<br>Hoogmoed, Jantien; Amsterdam UMC - Locatie AMC, Department of Neurosurgery, Neurosurgical Centre Amsterdam<br>Pereira, Alberto; Leiden University Medical Center, Department of Medicine, Division of Endocrinology, and Centre for Endocrine Tumors Leiden (CETL)<br>Biermasz, Nienke; Leiden University Medical Center, Department of Medicine, Division of Endocrinology, and Centre for Endocrine Tumors Leiden (CETL)<br>Simsek, Suat; Noordwest Ziekenhuisgroep, Department of Internal Medicine; Amsterdam UMC - Locatie VUMC, Department of Internal Medicine, Section of Endocrinology<br>Groote Veldman, Ronald; Medisch Spectrum Twente, Department of Internal Medicine<br>Tanck, Michael; Amsterdam UMC - Locatie AMC, Department of Clinical Epidemiology, Biostatistics and Bioinformatics<br>Fliers, Eric; Amsterdam UMC - Locatie AMC, Department of Endocrinology and Metabolism<br>Bisschop, Peter; Amsterdam UMC - Locatie AMC, Department of Endocrinology and Metabolism
**Primary Subject Heading**:	Diabetes and endocrinology
Secondary Subject Heading:	Research methods, Radiology and imaging
Keywords:	Pituitary disorders < DIABETES & ENDOCRINOLOGY, Endocrine tumours < DIABETES & ENDOCRINOLOGY, Nuclear radiology < RADIOLOGY & IMAGING, Clinical trials < THERAPEUTICS

SCHOLARONE™
Manuscripts

I, the Submitting Author has the right to grant and does grant on behalf of all authors of the Work (as defined in the below author licence), an exclusive licence and/or a non-exclusive licence for contributions from authors who are: i) UK Crown employees; ii) where BMJ has agreed a CC-BY licence shall apply, and/or iii) in accordance with the terms applicable for US Federal Government officers or employees acting as part of their official duties; on a worldwide, perpetual, irrevocable, royalty-free basis to BMJ Publishing Group Ltd ("BMJ") its licensees and where the relevant Journal is co-owned by BMJ to the co-owners of the Journal, to publish the Work in this journal and any other BMJ products and to exploit all rights, as set out in our licence.

The Submitting Author accepts and understands that any supply made under these terms is made by BMJ to the Submitting Author unless you are acting as an employee on behalf of your employer or a postgraduate student of an affiliated institution which is paying any applicable article publishing charge ("APC") for Open Access articles. Where the Submitting Author wishes to make the Work available on an Open Access basis (and intends to pay the relevant APC), the terms of reuse of such Open Access shall be governed by a Creative Commons licence – details of these licences and which Creative Commons licence will apply to this Work are set out in our licence referred to above.

Other than as permitted in any relevant BMJ Author's Self Archiving Policies, I confirm this Work has not been accepted for publication elsewhere, is not being considered for publication elsewhere and does not duplicate material already published. I confirm all authors consent to publication of this Work and authorise the granting of this licence.

For peer review only

The GALANT trial: study protocol of a randomised placebo-controlled trial in patients with a ^{68}*Ga*-DOTATATE PET-positive, clinically nonfunctioning pituitary macroadenoma on the effect of *lan*reotide on *tu*mour size

Tessel M. Boertien[1], Madeleine L. Drent[2], Jan Booij[3], Charles B. L. M. Majoie[3], Marcel P. M. Stokkel[4], Jantien Hoogmoed[5], Alberto M. Pereira[6], Nienke R. Biermasz[6], Suat Simsek[2,7], Ronald Groote Veldman[8], Michael W.T. Tanck[9], Eric Fliers[1]*, Peter H. Bisschop[1]*

[1]Department of Endocrinology and Metabolism, Amsterdam UMC, location AMC, University of Amsterdam, Meibergdreef 9, Amsterdam, the Netherlands
[2]Department of Internal Medicine, Section of Endocrinology, Amsterdam UMC, location VUMC, VU University, De Boelelaan 1117, Amsterdam, the Netherlands
[3]Department of Radiology and Nuclear Medicine, Amsterdam UMC, location AMC, University of Amsterdam, Meibergdreef 9, Amsterdam, the Netherlands
[4]Department of Nuclear Medicine, Netherlands Cancer Institute, Amsterdam, the Netherlands
[5]Department of Neurosurgery, Neurosurgical Centre Amsterdam, Amsterdam UMC, location AMC, University of Amsterdam, Meibergdreef 9, Amsterdam, the Netherlands
[6]Department of Medicine, Division of Endocrinology, and Centre for Endocrine Tumors Leiden (CETL), Leiden University Medical Centre, Leiden, the Netherlands
[7]Department of Internal Medicine, Northwest Clinics, Alkmaar, the Netherlands
[8]Department of Internal Medicine, Medical Spectrum Twente, Enschede, the Netherlands
[9]Department of Clinical Epidemiology, Biostatistics and Bioinformatics, Amsterdam UMC, location AMC, University of Meibergdreef 9, Amsterdam, the Netherlands
**Equal contribution*

Corresponding author: Tessel M. Boertien; t.m.boertien@amsterdamumc.nl
Amsterdam UMC, location AMC
Dept. of Endocrinology & Metabolism, K2-283
Meibergdreef 9
1105 AZ Amsterdam
THE NETHERLANDS

Word count:
Abstract: 265
Main text: 4,179

For peer review only - http://bmjopen.bmj.com/site/about/guidelines.xhtml

Abstract

Introduction At present, there is no approved medical treatment option for patients with nonfunctioning pituitary adenoma. A number of open-label studies suggest that treatment with somatostatin analogues may prevent tumour progression. *In vivo* somatostatin receptor imaging using ^{68}Ga-DOTATATE PET could help in preselecting patients potentially responsive to treatment. Our aim is to investigate the effect of the somatostatin analogue lanreotide as compared to placebo on tumour size in patients with a ^{68}Ga-DOTATATE PET-positive nonfunctioning pituitary macroadenoma (NFMA).

Methods and analysis The GALANT study is a multicentre, randomised, double-blind, placebo-controlled trial in adult patients with a suprasellar extending NFMA. Included patients undergo a ^{68}Ga-DOTATATE PET/CT of the head and tracer uptake is assessed after coregistration with pituitary MRI. Forty-four patients with a ^{68}Ga-DOTATATE PET-positive NFMA are randomised in a 1:1 ratio between lanreotide 120 mg or placebo, both administered as subcutaneous injections every 28 days for 72 weeks. The primary outcome is the change in cranio-caudal tumour diameter on pituitary MRI after treatment. Secondary outcomes are change in tumour volume, time to tumour progression, change in quality of life and number of adverse events. Final results are expected in the second half of 2021.

Ethics and dissemination The study protocol has been approved by the medical research ethics committee of the Academic Medical Centre (AMC) of the Amsterdam University Medical Centres and by the Dutch competent authority. It is an investigator-initiated study with financial support by Ipsen Farmaceutica BV. The AMC, as sponsor, remains owner of all data. Results will be submitted for publication in a peer-reviewed journal.

Trial registration number NL5136 (Netherlands Trial Register); pre-recruitment.

BMJ Open keywords: pituitary disorders, endocrine tumours, nuclear radiology, clinical trials

Strengths and limitations of this study

- The GALANT study is the first double-blind and placebo-controlled intervention trial in nonfunctioning pituitary macroadenoma patients.
- ^{68}Ga-DOTATATE PET/CT is used to select only those patients with a somatostatin receptor-positive adenoma for randomisation between lanreotide and placebo.
- The study is powered on change in cranio-caudal adenoma diameter, a clinically relevant outcome in the prevention of optic chiasm compression.
- The exclusion of patients previously treated with radiotherapy may present a bias in this study towards NFMA with a less aggressive growth rate.

For peer review only - http://bmjopen.bmj.com/site/about/guidelines.xhtml

76 ## List of abbreviations

^{111}In-DTPA-octreotide	Indium-111 labeled diethylenetriaminepentaacetic acid-octreotide
^{68}Ga-DOTATATE	Gallium-68 labeled dodecanetetraacetic acid-[Tyr^{3}]-octreotate
AMC	Academic Medical Centre (Amsterdam UMC, University of Amsterdam)
Amsterdam UMC	Amsterdam University Medical Centres
ANCOVA	analysis of covariance
CRF	case report form
CT	computer tomography
ITT	intention-to-treat
LUMC	Leiden University Medical Centre
MAR	missing at random
MBq	megabecquerel
MREC	medical research ethics committee
MRI	magnetic resonance imaging
mSv	millisievert
NFA	nonfunctioning adenoma
NFMA	nonfunctioning macroadenoma
PET	positron emission tomography
ROI	region of interest
SF-36	36-item Short Form Health Survey
SPECT	single-photon emission computed tomography
SPIRIT	Standard Protocol Items: Recommendations for Interventional Trials
(S)AE	(serious) adverse event
SSA	somatostatin analogue(s)
SSTR	somatostatin receptor
SUSAR	suspected unexpected serious adverse reaction
SUV	standard uptake value
VUMC	VU University Medical Center (Amsterdam UMC, VU University)

For peer review only

INTRODUCTION

Pituitary adenomas are benign tumours of the pituitary gland and are recognised as the third most common intracranial neoplasm.[1] Clinically nonfunctioning adenomas (NFA) account for 15-50% of all pituitary adenomas.[2] Because of the lack of clinical and biochemical signs of hormonal hypersecretion, NFA can go undetected for a long time and usually are macroadenomas, i.e. diameter ≥1cm, at time of diagnosis. Presenting symptoms are related to mass effects and include pituitary insufficiency, headache, and visual disturbances.[2]

Transsphenoidal resection of the adenoma is the main therapeutic approach. However, the overall remission rate after surgery is only 44%, and regrowth of residual tumour occurs in over 50% of patients.[3,4] Consequently, a substantial part of patients requires additional treatment during follow-up. Repeat surgery is recommended in case of persistent or recurrent chiasm compression, but the benefit/risk ratio is less favourable than with primary surgery.[5,6] Adjuvant radiotherapy is effective in controlling tumour (re)growth, but its use is restricted in view of the high risk of hypopituitarism and rare but important complications such as optic nerve damage and secondary brain tumours.[7–9]

Effective medical therapy as alternative option in NFA management would therefore be of great value. The expression of somatostatin receptor (SSTR) subtypes in the majority of NFA provides a potential target for receptor-mediated therapy.[10–16] The somatostatin analogues (SSA) octreotide and lanreotide have highest affinity for $SSTR_2$ and $SSTR_5$, and these subtypes are implicated in antiproliferative effects.[17] Indeed, treatment with octreotide seems to prevent tumour progression in selected NFA patients, although interpretation is hampered by methodological heterogeneity among studies and open-label design.[10,18]

SSA could thus play a role in the management of NFA patients, but outcomes of well-designed placebo-controlled studies are lacking. A promising approach may be *in vivo* SSTR imaging to preselect patients potentially responsive to treatment.[10,19] Thus far, SSTR expression in NFA has been assessed through ^{111}In-DTPA-octreotide scintigraphy or SPECT.[10,14,15,20] However, interpretation of pituitary adenoma uptake using scintigraphy/SPECT is limited by low spatial resolution.[21] The introduction of the PET radiotracer ^{68}Ga-DOTATATE has enabled high-resolution PET/CT imaging with additional superb $SSTR_2$ affinity.[22,23]

The aim of the present study is to investigate the effect of the SSA lanreotide, as compared to placebo, on adenoma size in patients with a ^{68}Ga-DOTATATE PET-positive nonfunctioning pituitary macroadenoma (NFMA).

METHODS AND ANALYSIS

This manuscript was written following the Standard Protocol Items: Recommendations for Interventional Trials (SPIRIT) guideline on reporting of intervention trial protocols (online supplementary material S1).[24,25]

For peer review only - http://bmjopen.bmj.com/site/about/guidelines.xhtml

Study design and setting

The GALANT study is a multicentre, randomised, double-blind, placebo-controlled, parallel-group, superiority trial in patients with a ^{68}Ga-DOTATATE PET-positive NFMA, investigating the effect of lanreotide versus placebo on tumour size. Forty-four patients are randomised in a 1:1 ratio and the primary outcome measure is the absolute change in cranio-caudal adenoma diameter after 72 weeks of treatment (study flowchart in **Figure 1**). The study is conducted in an outpatient setting and eligible patients are referred by endocrinologists at academic and non-academic hospitals in the Netherlands for inclusion at one of the participating centres (Amsterdam University Medical Centres (locations AMC and VUMC) and Leiden University Medical Centre). The study-related ^{68}Ga-DOTATATE PET/CT is performed at either the AMC or the Netherlands Cancer Institute in Amsterdam, an imaging-only participating centre. The study is investigator-initiated with the AMC as sponsor. Financial support is provided by Ipsen Farmaceutica BV.

Eligibility criteria

Adult patients diagnosed with an NFMA with suprasellar extension, either surgery-naïve or with a postoperative remnant, are eligible for inclusion. Detailed inclusion and exclusion criteria are listed in **Figure 2**. NFMA diagnosis is based on evidence for a pituitary macroadenoma on dedicated pituitary MRI and absence of clinical and biochemical signs of hormonal hypersecretion. Extension of the NFMA above the sellar diaphragm is a supplemental inclusion criterion since upward growth towards the optic nerves and chiasm is clinically relevant. In case of optic chiasm compression, current visual field defects must be excluded as this presents an indication for resection. Previous therapy with radiation, SSA or dopamine receptor agonists are exclusion criteria as these can influence the primary outcome. Other exclusion criteria are in accordance with the summary of product characteristics of lanreotide (Somatuline AutoSolution®).

Study outline

Eligible patients are approached by their treating endocrinologist and permission is asked to be contacted by a trial staff member (TB). The study is explained in full and written information is provided (online supplementary material S2 and S3). After obtaining written informed consent by a trial staff clinician, patient and NFMA characteristics are recorded (age, gender, medication use, laboratory and MRI results, current pituitary hormone deficiencies, prior NFMA treatment and further relevant medical history). At the screening visit, patients undergo a ^{68}Ga-DOTATATE PET/CT of the head. Only patients with a PET-positive NFMA are randomised for treatment. Before start of treatment, a baseline visit is planned consisting of a fasting blood sample, short physical examination, quality of life questionnaire, structured interview, and pregnancy test, if applicable. Pituitary MRI is only repeated at the baseline visit if time between the most recent MRI and planned start of study treatment exceeds 3 months or if the results provide any reason to repeat it. Study treatment consists of 18 subcutaneous injections administered every 28 days. Follow-up visits are planned at week 24 and week 48 (after the 6th and 12th injection, respectively), and the end-of-study visit takes place at week 72, approximately 4 weeks after the last injection. Study duration from first injection to last visit is thus 72 weeks. In case of withdrawal, a premature end-of-study visit is planned in the month after the last received injection. Study visits take place at the centre of inclusion (AMC, VUMC or LUMC) and will coincide with regular care assessments where possible. See **Figure 3** for the complete study schedule.

For peer review only - http://bmjopen.bmj.com/site/about/guidelines.xhtml

^{68}Ga-DOTATATE PET/CT

^{68}Ga-DOTATATE PET/CT of the head is performed at either the AMC or the Netherlands Cancer Institute (Gemini ToF, Philips Medical Systems and Biograph mCT, Siemens Healthineers). ^{68}Ga-DOTATATE preparation and quality control is performed according to a standard protocol as described elsewhere.[26] Images are acquired approximately 45 minutes after intravenous bolus injection of 100 MBq ^{68}Ga-DOTATATE with 2.5-3 min per bed position. A low-dose CT scan is acquired for attenuation correction and anatomical correlation. The total estimated radiation exposure of the combined PET/CT is 3.1 mSv.[27] PET/CT and pituitary MR images are co-registered manually using Hybrid Viewer (Hermes Medical Solutions). On the fused PET/MR images, ^{68}Ga-DOTATATE uptake is assessed by placement of a circular region of interest (ROI) within the adenoma while maintaining a clear margin from normal pituitary tissue, and determining the mean standard uptake value (SUV_{mean}).[23] An SUV_{mean} of >2 is considered as positive uptake. In the absence of literature on ^{68}Ga-DOTATATE uptake in pituitary adenomas, this value has been chosen to reflect a level of uptake at least similar to that of the normal pituitary, based on a ^{68}Ga-DOTATATE biodistribution study in 42 subjects demonstrating physiological pituitary uptake with a minimum SUV_{mean} of 2.1.[28]

Intervention

Forty-four patients with a ^{68}Ga-DOTATATE PET-positive NFMA are randomised between treatment with either the extended-release formulation of lanreotide 120 mg (Somatuline AutoSolution®, Ipsen Farmaceutica BV; known outside the Netherlands as Somatuline Autogel®) without dose titration or placebo (consisting of 0.4 mL saline 0.9%), both administered every 28 days as a deep subcutaneous injection in the superior, external quadrant of the buttock. Study treatment consists of a total of 18 injections. The injections are administered by experienced and trained nurses, either at the AMC endocrine unit or at home via a homecare service (Eurocept Homecare). Administration details are recorded at each nurse visit. Lanreotide 120 mg is the highest dose currently available. Treatment with this dose has shown to be safe and well-tolerated in two efficacy studies in enteropancreatic neuroendocrine tumours and acromegaly.[29,30] A safety review on SSA dose optimisation and a phase 2 RCT specifically investigating lanreotide in different dosages did not demonstrate significant changes in adverse effects and tolerability with increasing dose. Higher doses were, however, related to greater efficacy.[31,32]

Randomisation

Randomisation to treatment is done by means of a computer-generated randomisation list through Sealed Envelope Ltd (https://www.sealedenvelope.com/simple-randomiser/v1/lists) using blocked randomisation with an allocation ratio of 1:1 and block size of four.

Allocation concealment and blinding

Randomisation is performed by the AMC trial pharmacy. The randomisation list is stored in the pharmacy trial file and will not be disclosed until the last participant has completed the trial. In case of emergency the randomisation list can be accessed by the hospital pharmacist on call and the randomisation code can be broken if the safety of the patient requires knowledge of the assigned treatment (e.g. in case of a suspected unexpected serious adverse reaction (SUSAR)). Breaking of the code will be requested by an independent trial staff member to maintain allocation concealment to investigators assessing the outcomes. The study is blinded for participants, investigators and treating physicians. Because the placebo injections differ in appearance from the lanreotide (Somatuline

For peer review only - http://bmjopen.bmj.com/site/about/guidelines.xhtml

AutoSolution®) pre-filled syringes, pharmacy employees preparing the treatment and nurses administering the injections cannot be blinded.[29] As the injections are administered into the superior, external quadrant of the buttock, patients will not see the syringes. Furthermore, patients have no earlier experience with lanreotide treatment to have expectations concerning the administration. To maintain blinding during transport and storage, the study medication is placed in an opaque bag within a sealed cardboard box with a "blinded study medication" warning message.

Assessments during the study

Pituitary MRI

Pituitary MRIs are performed regularly as part of standard care of NFMA patients. Study participation is timed in such a way that visits coincide with regular MRI planning where possible. MRI scans are performed on a 1.5 or 3 Tesla scanner, following a pituitary-specific protocol that includes T1-weighted coronal and sagittal acquisitions before and after gadolinium administration, a T2-weighted coronal sequence, and preferably a volumetric 3DT1-weighted sequence.

Laboratory tests

Regular laboratory assessments are also part of standard care. During the study, laboratory tests will include an endocrine evaluation (TSH, free T4, cortisol, IGF-1, prolactin, and testosterone or oestradiol), fasting glucose, glycated haemoglobin (HbA1c), sodium, potassium, kidney function, liver enzymes, and bilirubin. At baseline and week 72, additional blood samples are obtained for alpha subunit assessment. Leftover serum and plasma will be stored according to local regulatory guidelines until publication of results.

Physical examination

Measurement of height, weight, blood pressure and pulse rate takes place at each visit after randomisation. More extensive examination is only performed if indicated.

Quality of life questionnaire

The 36-item Short Form Health Survey (SF-36), validated Dutch translation, is used to assess quality of life at study visits.[33] The SF-36 is comprised of 36 items that generate 8 component scores: physical functioning, role limitations due to physical health problems, bodily pain, general health perceptions, vitality, social functioning, role limitations due to emotional problems, and general mental health. Scores are expressed on a 0-100 scale with higher scores indicating better quality of life for that component.

Structured interview

The structured interview at visits during treatment consists of health-related questions based on possible side-effects of lanreotide, other signs and symptoms experienced between visits, and changes in medication use.

Adverse events

Adverse events (AEs) can be reported throughout the study period and will be inquired about specifically at study and nurse visits. An AE is defined as any undesirable finding or experience occurring to a subject during the study, whether or not considered related to study treatment. The type of AE, times of onset and resolution, severity, and possible relationship with lanreotide will be recorded.

For peer review only - http://bmjopen.bmj.com/site/about/guidelines.xhtml

Concomitant care and post-trial follow-up

Participants are not allowed to use medication or participate in other trials aimed at adenoma reduction. Otherwise, patient care is continued as usual. Participants may withdraw from the study for any reason at any time. In case of development of visual field impairments or signs pointing to cranial nerve injury, patients will undergo additional pituitary MRI and visual field examination as is standard practice. If intervention is deemed necessary, study treatment is stopped. Patients can also be withdrawn based on the investigator's or treating specialist's judgement, e.g. in case of an AE that could jeopardise patient safety. Premature study withdrawal will have no effect on regular patient care.

Lanreotide can inhibit the secretion of insulin and glucagon. Patients on antidiabetic therapy are therefore advised to monitor their blood glucose levels more closely after start of study treatment and have their medication adjusted accordingly. Concomitant use of bradycardia inducing drugs (e.g. beta blockers) may have an additive effect on the slight reduction of heart rate associated with lanreotide and dose adjustments may be necessary. Intestinal absorption of most notably ciclosporin can be reduced, requiring close review of therapeutic levels. Participants are instructed to notify the research team if they have been prescribed a new drug by their general practitioner or specialist during the study.

After completion of study treatment or premature withdrawal, standard care is continued. Patients who leave the study for medical reasons/AEs will be followed until the interfering condition has resolved or reached a stable state. All participants will be followed until completion of the trial and will be notified of the results.

Primary outcome

The primary outcome is the absolute change in cranio-caudal tumour diameter in millimetres from baseline to week 72 (or last available post-baseline measurement). Tumour size will be determined on pituitary MRI by two neuroradiologists blinded to treatment allocation and scan chronology. The measurements of both radiologists are averaged for the analysis. In case of a between-reader difference of ≥10% or ≥2mm, the images will be offered for reassessment. In case of a persistent difference of ≥10% or ≥2mm, a third neuroradiologist will review the images and the measurements of the three radiologists are averaged for the analysis. A change in tumour size of ≥2mm is considered clinically relevant, as such a difference is sufficient to prevent complications related to tumour growth. Even a small increase in cranio-caudal diameter can be reason for intervention via surgery or radiotherapy to prevent or relieve compression of the optic nerves or chiasm.[34] Moreover, the value of 2mm is commonly used as cut-off to reliably detect a change in tumour size on pituitary MRI.[35–37]

Secondary outcomes

Change in tumour volume

As with the primary outcome, the change in tumour volume between baseline and week 72 (or last available post-baseline measurement) is determined by two neuroradiologists or trained and supervised study personnel using pituitary MR images. Tumour volume in cubic millimetres will be calculated using a semi-automatic segmentation tool in the coronal plane. On each slice the tumour is delineated and the surface area multiplied by the slice thickness. The total tumour volume is

For peer review only - http://bmjopen.bmj.com/site/about/guidelines.xhtml

determined by summing up the slice volumes. The slice-by-slice segmentation method is preferred to the traditional geometric formula (i.e. ½ x length x width x height) due to the frequent irregular shape and invasion of NFMA, especially if it concerns a postoperative remnant.[38] In case of a between-reader difference of ≥10%, the images will be offered for reassessment. In case of a persistent difference of ≥10%, a third neuroradiologist or experienced study staff member will review the images. A change in tumour volume of ≥20% will be considered clinically significant.[30]

Time to progression

The presence or absence of tumour progression will be based on clinically significant change in tumour volume between baseline and subsequent pituitary MRIs. Tumour progression is defined as an increase in volume of ≥20%, absence of progression comprises both tumour shrinkage (volume decrease ≥20%) and stable disease (volume change <20%).[30]

Change in quality of life

The change in quality of life is based on the SF-36 questionnaire component scores obtained at the study visits.

Number of (serious) AEs

As a safety endpoint, the number and type of AEs and serious AEs (SAEs) recorded during the study will be compared between the groups.

Sample size

The study is powered on a between-group difference in primary outcome of ≥2mm. In order to detect a mean difference of 2mm with a standard deviation of 1.9mm, we need to randomise 16 patients per group based on a 2-sided independent *t*-test with 80% power and 5% type I error risk (nQuery Advisor 7.0, Statsol, Boston, Massachusetts, USA). The final analysis of the primary endpoint will be performed by ANCOVA. While ANCOVA may reduce the required sample size, a reliable estimation of rho is not possible for this study and a more conservative *t*-test-based power analysis is therefore less likely to result in an underpowered study.[39] Taking into account an observed dropout rate of around 25%, as a result of withdrawals due to AEs or need for intervention, the sample size has been increased to 22 patients per group (substantial amendment, protocol version 5.0). Dropout is defined as failure to complete the 72 weeks of treatment due to any reason. Only patients with a ^{68}Ga-DOTATATE PET-positive NFMA are randomised for treatment. Based on previous reports that about two-thirds of NFA patients showed increased adenoma uptake using ^{111}In-DTPA-octreotide scintigraphy[10,14,15,40] and considering the superior sensitivity of ^{68}Ga-DOTATATE PET/CT, we expected to enrol a maximum of 66 patients in order to randomise 44 patients.

Statistical analyses

Data of continuous variables will be summarised as either means with standard deviation or medians with interquartile range, according to distribution. Normality of distribution will be assessed visually with histograms, Q-Q plots and using the Shapiro-Wilk test. Data of categorical variables will be reported as incidence rates (number and percentage). Baseline characteristics and ^{68}Ga-DOTATATE PET/CT results of all included subjects will be reported. Data on recruitment and dropout will also be reported. Differences between patients with positive and negative ^{68}Ga-DOTATATE uptake will be evaluated. Group differences in continuous variables are analysed using the Student's *t*-test or Mann-Whitney U test, depending on data distribution. Group differences in categorical variables are

For peer review only - http://bmjopen.bmj.com/site/about/guidelines.xhtml

analysed using either the Chi-square or the Fisher's exact test, as appropriate. In principle, since patients are randomly allocated to treatment, differences in baseline characteristics between the groups should not be analysed. When performing linear regression analyses or linear mixed effects models, assumptions for linearity, distribution and/or homoscedasticity will be checked for each model using scatter plots and residual analysis.

Outcome data will be analysed according to intention-to-treat (ITT). The ITT population is defined as all randomised patients having received at least one study injection. In case of withdrawal after treatment initiation, effort is made to plan a premature end-of-study visit to obtain outcome data for the ITT analyses, unless assessments were performed within the previous 4 weeks. In case of incomplete/missing outcome data, the assumption that data is missing at random (MAR) will be checked. If the MAR assumption is plausible, a main analysis using all observed data is performed (e.g. a mixed model). This main analysis does not require the inclusion of subjects that lack post-baseline assessments.[41] Sensitivity analyses including all ITT subjects are then performed to explore the impact of departures from this assumption.[41] This includes an analysis using multiple imputation in case of missing outcome data.[42]

The difference between groups in primary outcome and secondary outcome change in tumour volume will be analysed using one-way ANCOVA, controlling for baseline adenoma size. For these two endpoints, a separate per-protocol efficacy analysis will be performed, including only those subjects that have completed all 18 study injections and final MRI. Additionally, the proportion of patients with a clinically significant change in tumour size (i.e. ≥2mm change in cranio-caudal size or ≥20% change in tumour volume) is reported. Time to tumour progression is analysed using the Kaplan-Meier method. Missing cases due to withdrawal without outcome data will be censored. Differences in distribution and percentage of censoring between groups will be evaluated. Between-group differences in time to progression are analysed using the stratified logrank test, with stratification for presence or absence of tumour growth at baseline. To quantify the difference, the hazard ratio and confidence intervals are estimated using the Cox proportional-hazards model. The SF-36 component scores will be presented as spider charts. The mean imputation method will be used to replace missing values. Results will be compared with Dutch population reference values[33] and the change in relevant component scores are compared between the groups. All patients that received at least one study injection will be included in the safety endpoint analysis of AEs independent of post-baseline assessments.

As the study remains blinded until completion, no interim analyses are planned. There are no pre-specified subgroups. A *p*-value <0.05 will be considered statistically significant. In case of multiple testing, Bonferroni correction will be applied where appropriate. Besides the reporting of *p*-values, 95% confidence intervals around point estimates will be provided for more useful interpretation of results. Analyses will be done using SPSS (IBM, v25 or later if available) and/or RStudio (R Foundation for Statistical Computing, v3.6.1, 2019-07-05, or later if available). Before outcome analyses are initiated, the statistical analysis plan will be finalised.

Data management

Data is collected using Castor Electronic Data Capture (EDC).[43] The electronic case report forms (CRFs) have built-in data validity and value range checks. Source documents include medical records, records of the Eurocept homecare service, SF-36 questionnaires and paper CRFs documenting

For peer review only - http://bmjopen.bmj.com/site/about/guidelines.xhtml

injections administered at the AMC. All subject data is pseudonymised with a study code. The subject identification log which links subjects to the code is kept in a trial file only accessible to study personnel. All research data will be stored for 15 years. Leftover serum and plasma from blood samples obtained for standard care during the study will be stored according to local regulatory guidelines until publication of the results and then destroyed. Data will be handled in accordance with the principles of the General Data Protection Regulation. All data management procedures are detailed in a separate data management plan.

Safety

The most commonly expected side-effects of lanreotide are gastrointestinal disorders (e.g. diarrhoea, abdominal pain and nausea, usually mild/moderate and transient), cholelithiasis (often asymptomatic) and injection site reactions. Additionally, lanreotide may induce (mild) hypoglycaemia or hyperglycaemia due to inhibition of insulin and/or glucagon secretion. Because of surveillance at regular intervals during the study the risks are small. Throughout the study, AEs reported by the patient or observed by the investigators or healthcare providers are recorded in the medical file and study database. All SAEs including SUSARs are reported to the medical research ethics committee (MREC) of the AMC. In addition, an annual safety report is submitted.

Monitoring

The trial is monitored by the Clinical Research Unit of the AMC. Participating centres agree to allow monitoring by providing access to source data and documents as required. Every participating centre is subjected to a start-up visit after three included patients, a second site-visit after approximately ten inclusions and a combined monitoring and site close-out visit after completion of data collection. The monitor confirms all written informed consents, checks all SAEs and investigates data collection and data quality for a random subset of patients. The frequency and intensity of monitoring can be changed based on findings during visits. Monitoring procedures are detailed in a separate monitoring plan.

Patient and public involvement

Patients were not involved in the design of this study. The study was highlighted in “Hyponieuws”, the magazine of the Dutch Pituitary Foundation, which is the Dutch pituitary patient platform.

ETHICS AND DISSEMINATION

The study protocol has been approved by the accredited MREC of the AMC and by the Dutch competent authority. Permission for execution of the study is obtained at each participating site. Substantial amendments to the protocol are submitted for approval and communicated to participating centres and trial registries. The study is conducted in accordance with the Declaration of Helsinki, Good Clinical Practice guidelines, and local regulatory requirements. Participant insurance is provided by the AMC for all participating subjects.

Regardless of the outcome, study results will be submitted for publication in a peer-reviewed international medical journal and presented at (inter)national conferences. The International Committee of Medical Journal Editors guidelines for authorship will be followed. The AMC, as

For peer review only - http://bmjopen.bmj.com/site/about/guidelines.xhtml

sponsor, will remain owner of all data and have all publication rights. Full protocol, datasets and statistical analysis plan will be available from the corresponding author on reasonable request after publication of the final results.

Trial status

Recruitment started on November 3rd 2015. At the time of preparing this manuscript, inclusion has been completed. The last visit of the last patient is expected in the first half of 2021.

DISCUSSION

The GALANT study is the first double-blind and placebo-controlled intervention trial in NFMA patients, investigating the effect of lanreotide 120 mg on NFMA size. Treatment duration is 72 weeks, in line with the slow average NFA growth rate of around 1 mm/year.[4,35] An additional strength of the study design is the use of ^{68}Ga-DOTATATE PET/CT to select only those patients with an SSTR-positive adenoma for treatment. Although in a clinical setting dose titration might mitigate side-effects and prevent treatment cessation, use of the highest dose currently available is justified in order to prove efficacy for a possible new therapeutic indication and ensures data homogeneity. Furthermore, treatment with this dose has shown to be safe and well-tolerated in earlier trials.[29–31] The study is powered to detect a between-group difference of ≥2 mm change in cranio-caudal tumour size after treatment, a clinically relevant outcome. Consistent and unbiased tumour measurements are ensured through assessment by at least two neuroradiologists blinded for treatment allocation and scan chronology. If lanreotide proves effective in reducing or controlling tumour size, this would present an important new treatment option for NFMA patients.

Supplementary files

The following supplementary files to this article can be found online at ***[… fill-in-DOI]***:
Supplementary file S1: Completed SPIRIT Checklist. (PDF, 184 kB)
Supplementary file S2: Patient information letter and informed consent form, Dutch AMC version. (PDF, 606 kB)
Supplementary file S3: Patient information letter and informed consent form, English AMC version. (PDF, 344 kB)

Footnotes

Contributors: TB, MD, JB, CM, MS, MT, PB and EF participated in the design of the study. EF, MD, MS and AP are principal investigators at the participating study centres. EF supervises the study in name of the sponsor (AMC). MD, JH, AP, NB, SS, RGV, PB and EF assist with the referral and inclusion of patients. TB drafted the manuscript with help of PB and EF. All authors reviewed and approved the final manuscript.

Funding: This work is supported by Ipsen Farmaceutica BV (the Netherlands). The GALANT study is investigator-initiated; the Academic Medical Centre (AMC) is the sponsor of the study. The funding

For peer review only - http://bmjopen.bmj.com/site/about/guidelines.xhtml

party has played no role in the final study design or study conduct nor in the preparation of this manuscript or the decision to submit it for publication. The AMC, as sponsor, will remain owner of all data and have all publication rights.

Competing interests: MD received a honorarium for chairing a symposium sponsored by Ipsen Farmaceutica BV in 2019. No other potential conflicts of interest have been declared.

Ethics approval: Medical Ethics Committee Academic Medical Centre, of the Amsterdam University Medical Centres (reference: NL 52821.018.15).

Trial registration: EudraCT: 2015-001234-22, registered 10 March 2015; Netherlands Trial Register (NTR): NL5136, registered 18 August 2015. Registration took place before start of recruitment.

Protocol versions:

- V2.0, May 21st 2015: first approved protocol version.
- V3.0, October 13th 2016: new participating centre (LUMC) added to achieve target recruitment goal; possibility to perform the ^{68}Ga-DOTATATE PET/CT at the AMC; possibility for patients to have study injections administered at home by a specialised homecare company with trained nurses (Eurocept Homecare).
- V4.0, August 31st 2017: clearer exclusion criterion concerning dopamine agonist use: “Use of dopamine agonists” changed to “Use of dopamine agonist in the past 6 months”.
- V5.0, November 30th 2018: increased number of randomisations to account for dropouts and more detailed statistical analysis section (including handling of missing/incomplete data). Current protocol version.

Data sharing statement: The datasets generated and/or analysed during the current study will be available from the corresponding author on reasonable request after publication of the final results.

Provenance and peer review: Not commissioned; externally peer reviewed.

Open Access: This is an Open Access article distributed in accordance with the Creative Commons Attribution Non Commercial (CC BY-NC 4.0) license, which permits others to distribute, remix, adapt, build upon this work non-commercially, and license their derivative works on different terms, provided the original work is properly cited and the use is non-commercial. See: https://creativecommons.org/licenses/by-nc/4.0/

References

1 Aflorei ED, Korbonits M. Epidemiology and etiopathogenesis of pituitary adenomas. *J Neurooncol* 2014;**117**:379–94. doi:10.1007/s11060-013-1354-5

2 Ntali G, Wass JA. Epidemiology, clinical presentation and diagnosis of non-functioning pituitary adenomas. *Pituitary* 2018;**21**:111–8. doi:10.1007/s11102-018-0869-3

3 Roelfsema F, Biermasz NR, Pereira AM. Clinical factors involved in the recurrence of pituitary adenomas after surgical remission: A structured review and meta-analysis. *Pituitary* 2012;**15**:71–83. doi:10.1007/s11102-011-0347-7

4 Chen Y, Wang C De, Su ZP, *et al.* Natural History of Postoperative Nonfunctioning Pituitary Adenomas: A

For peer review only - http://bmjopen.bmj.com/site/about/guidelines.xhtml

Systematic Review and Meta-Analysis. *Neuroendocrinology* 2012;**96**:333–42. doi:10.1159/000339823
5 Sheehan J, Lee C-C, Bodach ME, *et al.* Congress of Neurological Surgeons Systematic Review and Evidence-Based Guideline for the Management of Patients With Residual or Recurrent Nonfunctioning Pituitary Adenomas. *Neurosurgery* 2016;**79**:E539–40. doi:10.1227/NEU.0000000000001385
6 Cortet-Rudelli C, Bonneville J-F, Borson-Chazot F, *et al.* Post-surgical management of non-functioning pituitary adenoma. *Ann Endocrinol (Paris)* 2015;**76**:228–38. doi:10.1016/j.ando.2015.04.003
7 Minniti G, Flickinger J, Tolu B, *et al.* Management of nonfunctioning pituitary tumors: radiotherapy. *Pituitary* 2018;**21**:154–61. doi:10.1007/s11102-018-0868-4
8 Dekkers OM, Pereira AM, Romijn JA. Treatment and follow-up of clinically nonfunctioning pituitary macroadenomas. *J Clin Endocrinol Metab* 2008;**93**:3717–26. doi:10.1210/jc.2008-0643
9 Greenman Y. MANAGEMENT OF ENDOCRINE DISEASE: Present and future perspectives for medical therapy of nonfunctioning pituitary adenomas. *Eur J Endocrinol* 2017;**177**:R113–24. doi:10.1530/EJE-17-0216
10 Fusco A, Giampietro A, Bianchi A, *et al.* Treatment with octreotide LAR in clinically non-functioning pituitary adenoma: Results from a case-control study. *Pituitary* 2012;**15**:571–8. doi:10.1007/s11102-011-0370-8
11 Lee M, Lupp A, Mendoza N, *et al.* SSTR3 is a putative target for the medical treatment of gonadotroph adenomas of the pituitary. *Endocr Relat Cancer* 2015;**22**:111–9. doi:10.1530/ERC-14-0472
12 Ramírez C, Cheng S, Vargas G, *et al.* Expression of Ki-67, PTTG1, FGFR4, and SSTR 2, 3, and 5 in Nonfunctioning Pituitary Adenomas: A High Throughput TMA, Immunohistochemical Study. *J Clin Endocrinol Metab* 2012;**97**:1745–51. doi:10.1210/jc.2011-3163
13 Gabalec F, Drastikova M, Cesak T, *et al.* Dopamine 2 and somatostatin 1-5 receptors coexpression in clinically non-functioning pituitary adenomas. *Physiol Res* 2015;**64**:369–77.http://www.ncbi.nlm.nih.gov/pubmed/25536318
14 Plöckinger U, Bäder M, Hopfenmüller W, *et al.* Results of somatostatin receptor scintigraphy do not predict pituitary tumor volume- and hormone-response to ocreotide therapy and do not correlate with tumor histology. *Eur J Endocrinol* 1997;**136**:369–76.http://www.ncbi.nlm.nih.gov/pubmed/9150695
15 Borson-Chazot F, Houzard C, Ajzenberg C, *et al.* Somatostatin receptor imaging in somatotroph and non-functioning pituitary adenomas: correlation with hormonal and visual responses to octreotide. *Clin Endocrinol (Oxf)* 1997;**47**:589–98. doi:10.1046/j.1365-2265.1997.3361119.x
16 Reubi JC, Waser B, Schaer J-C, *et al.* Somatostatin receptor sst1-sst5 expression in normal and neoplastic human tissues using receptor autoradiography with subtype-selective ligands. *Eur J Nucl Med Mol Imaging* 2001;**28**:836–46. doi:10.1007/s002590100541
17 Bevan JS. The Antitumoral Effects of Somatostatin Analog Therapy in Acromegaly. *J Clin Endocrinol Metab* 2005;**90**:1856–63. doi:10.1210/jc.2004-1093
18 Colao A, Di Somma C, Pivonello R, *et al.* Medical therapy for clinically non-functioning pituitary adenomas. *Endocr Relat Cancer* 2008;**15**:905–15. doi:10.1677/ERC-08-0181
19 Melmed S. Pituitary Medicine From Discovery to Patient-Focused Outcomes. *J Clin Endocrinol Metab* 2016;**101**:769–77. doi:10.1210/jc.2015-3653
20 Colao A, Lastoria S, Ferone D, *et al.* The pituitary uptake of 111In-DTPA-D-Phe1-octreotide in the normal pituitary and in pituitary adenomas. *J Endocrinol Invest* 1999;**22**:176–83.http://www.embase.com/search/results?subaction=viewrecord&from=export&id=L29158636
21 de Herder WW, Kwekkeboom DJ, Feelders RA, *et al.* Somatostatin receptor imaging for neuroendocrine tumors. *Pituitary* 2006;**9**:243–8. doi:10.1007/s11102-006-0270-5
22 Reubi JC, Schaer J-C, Waser B, *et al.* Affinity profiles for human somatostatin receptor subtypes SST1-SST5 of somatostatin radiotracers selected for scintigraphic and radiotherapeutic use. *Eur J Nucl Med Mol Imaging* 2000;**27**:273–82.http://www.ncbi.nlm.nih.gov/pubmed/10774879
23 Bai B, Bading J, Conti PS. Tumor quantification in clinical positron emission tomography. *Theranostics* 2013;**3**:787–801. doi:10.7150/thno.5629
24 Chan A-W, Tetzlaff JM, Gøtzsche PC, *et al.* SPIRIT 2013 explanation and elaboration: guidance for protocols of clinical trials. *BMJ* 2013;**346**:e7586–e7586. doi:10.1136/bmj.e7586
25 Chan A-W, Tetzlaff JM, Altman DG, *et al.* SPIRIT 2013 Statement: Defining Standard Protocol Items for Clinical Trials. *Ann Intern Med* 2013;**158**:200. doi:10.7326/0003-4819-158-3-201302050-00583
26 Aalbersberg EA, Geluk-Jonker MM, Young-Mylvaganan T, *et al.* A Practical Guide for the Production and PET/CT Imaging of 68Ga-DOTATATE for Neuroendocrine Tumors in Daily Clinical Practice. *J Vis Exp* 2019;:1–2. doi:10.3791/59358
27 Walker RC, Smith GT, Liu E, *et al.* Measured human dosimetry of 68Ga-DOTATATE. *J Nucl Med*

For peer review only - http://bmjopen.bmj.com/site/about/guidelines.xhtml

2013;**54**:855–60. doi:10.2967/jnumed.112.114165
28 Shastry M, Kayani I, Wild D, *et al.* Distribution pattern of 68Ga-DOTATATE in disease-free patients. *Nucl Med Commun* 2010;**31**:1. doi:10.1097/MNM.0b013e32833f635e
29 Caplin ME, Pavel M, Cwikła JB, *et al.* Lanreotide in metastatic enteropancreatic neuroendocrine tumors. *N Engl J Med* 2014;**371**:224–33. doi:10.1056/NEJMoa1316158
30 Caron PJ, Bevan JS, Petersenn S, *et al.* Tumor shrinkage with lanreotide autogel 120 mg as primary therapy in acromegaly: Results of a prospective multicenter clinical trial. *J Clin Endocrinol Metab* 2014;**99**:1282–90. doi:10.1210/jc.2013-3318
31 Ludlam WH, Anthony L. Safety review: Dose optimization of somatostatin analogs in patients with acromegaly and neuroendocrine tumors. *Adv Ther* 2011;**28**:825–41. doi:10.1007/s12325-011-0062-9
32 Bronstein M, Musolino N, Jallad R, *et al.* Pharmacokinetic profile of lanreotide Autogel?? in patients with acromegaly after four deep subcutaneous injections of 60, 90 or 120 mg every 28 days. *Clin Endocrinol (Oxf)* 2005;**63**:514–9. doi:10.1111/j.1365-2265.2005.02372.x
33 Aaronson NK, Muller M, Cohen PDA, *et al.* Translation, validation, and norming of the Dutch language version of the SF-36 Health Survey in community and chronic disease populations. *J Clin Epidemiol* 1998;**51**:1055–68. doi:10.1016/S0895-4356(98)00097-3
34 Molitch ME. Diagnosis and Treatment of Pituitary Adenomas. *JAMA* 2017;**317**:516. doi:10.1001/jama.2016.19699
35 Dekkers OM, Hammer S, de Keizer RJW, *et al.* The natural course of non-functioning pituitary macroadenomas. *Eur J Endocrinol* 2007;**156**:217–24. doi:10.1530/eje.1.02334
36 O'Sullivan EP, Woods C, Glynn N, *et al.* The natural history of surgically treated but radiotherapy-naïve nonfunctioning pituitary adenomas. *Clin Endocrinol (Oxf)* 2009;**71**:709–14. doi:10.1111/j.1365-2265.2009.03583.x
37 Brochier S, Galland F, Kujas M, *et al.* Factors predicting relapse of nonfunctioning pituitary macroadenomas after neurosurgery: A study of 142 patients. *Eur J Endocrinol* 2010;**163**:193–200. doi:10.1530/EJE-10-0255
38 Chuang C-C, Lin S-Y, Pai P-C, *et al.* Different Volumetric Measurement Methods for Pituitary Adenomas and Their Crucial Clinical Significance. *Sci Rep* 2017;**7**:40792. doi:10.1038/srep40792
39 Borm GF, Fransen J, Lemmens WAJG. A simple sample size formula for analysis of covariance in randomized clinical trials. *J Clin Epidemiol* 2007;**60**:1234–8. doi:10.1016/j.jclinepi.2007.02.006
40 Duet M, Ajzenberg C, Benelhadj S, *et al.* Somatostatin receptor scintigraphy in pituitary adenomas: a somatostatin receptor density index can predict hormonal and tumoral efficacy of octreotide in vivo. *J Nucl Med* 1999;**40**:1252–6.http://www.ncbi.nlm.nih.gov/pubmed/10450674
41 White IR, Carpenter J, Horton NJ. Including all individuals is not enough: Lessons for intention-to-treat analysis. *Clin Trials* 2012;**9**:396–407. doi:10.1177/1740774512450098
42 Sterne JAC, White IR, Carlin JB, *et al.* Multiple imputation for missing data in epidemiological and clinical research: potential and pitfalls. *BMJ* 2009;**338**:b2393. doi:10.1136/bmj.b2393
43 Castor E. Castor Electronic Data Capture. 2019.https://castoredc.com

For peer review only

For peer review only - http://bmjopen.bmj.com/site/about/guidelines.xhtml

Figure legends

Figure 1 Flowchart of the GALANT (a randomized placebo-controlled trial in patients with a ^{68}*Ga*-DOTATATE PET-positive, clinically nonfunctioning pituitary macroadenoma on the effect of *lan*reotide on *t*umour size) study. NFMA, nonfunctioning pituitary macroacdenoma

Figure 2 Eligibility criteria.

Figure 3 Standard Protocol Items: Recommendations for Interventional Trials (SPIRIT) figure. Schedule of enrolment, interventions, and assessments in the GALANT study. SF-36, 36-item Short Form Health Survey.

[a] Treatment consists of 18 injections administered every 28 days.

[b] In case of study withdrawal, a premature end-of-study visit is planned in the month after the last received injection.

[c] Patient age, gender, medication use, most recent laboratory and pituitary MRI results, current pituitary hormone deficiencies, prior treatment and further relevant medical history.

[d] Only in female patients of childbearing potential.

[e] Pituitary MRI is only repeated if time between the most recent MRI and planned start of study treatment exceeds three months or if results give cause to repeat it.

[f] Endocrine evaluation, fasting glucose, glycated haemoglobin (HbA1c), sodium, potassium, kidney function, liver enzymes, and bilirubin; additional sample at baseline and close-out for alpha subunit assessment.

[g] Measurement of height, weight, blood pressure and pulse rate; more extensive examination if indicated.

[h] Unresolved adverse events at time of end-of-study visit are followed up until resolved or stabilised.

For peer review only - http://bmjopen.bmj.com/site/about/guidelines.xhtml

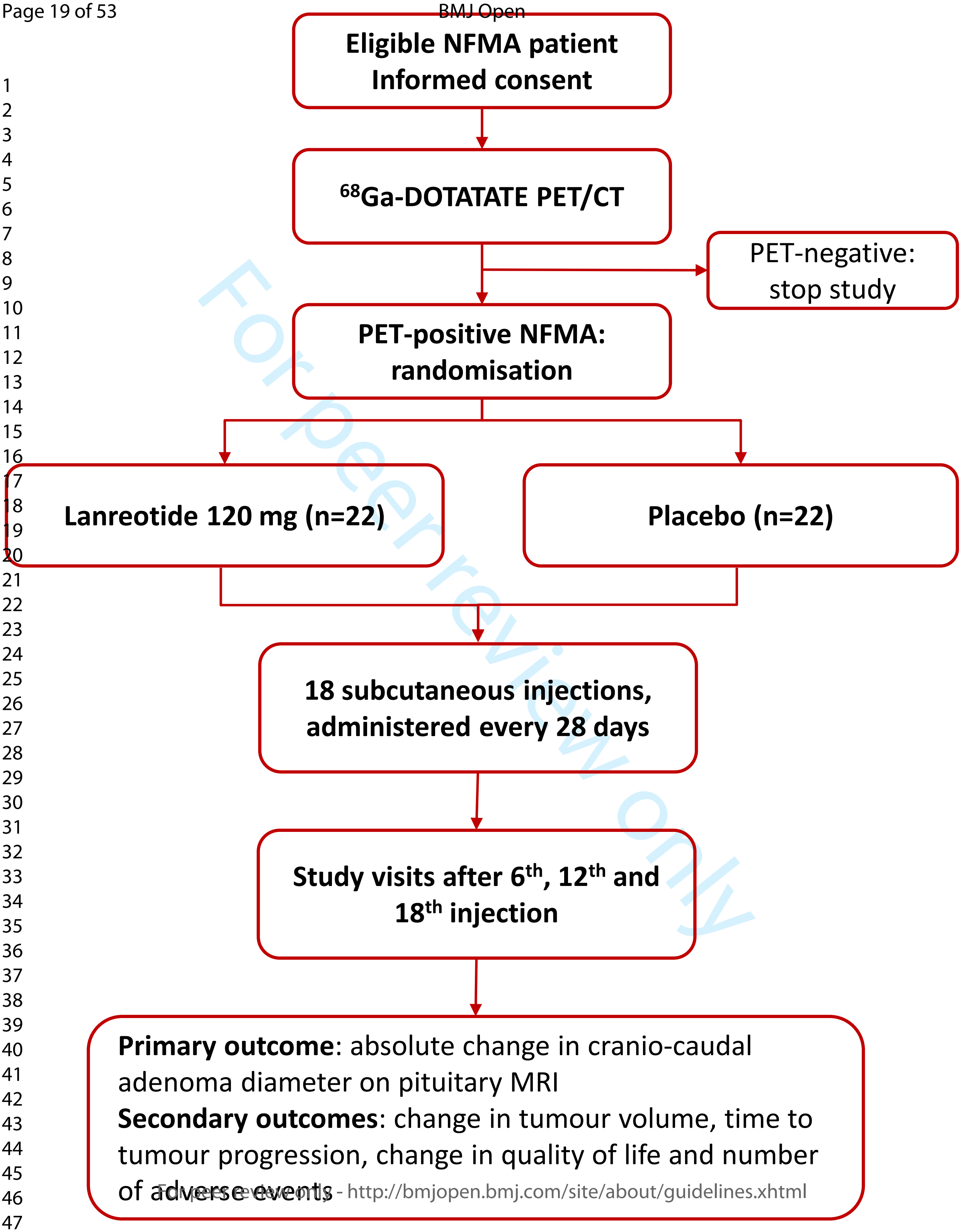

For peer review only

Inclusion criteria

- Clinically nonfunctioning pituitary macroadenoma extending above the sellar diaphragm
- Age ≥ 18 years
- Written informed consent

Exclusion criteria

- Optic chiasm compression with visual field defects
- Previous radiation therapy in the pituitary region
- Previous use of somatostatin analogues
- Use of dopamine receptor agonists in the past 6 months
- Hypersensitivity to somatostatin or similar peptides
- Obstructive neuroendocrine gut tumour
- Symptomatic cholelithiasis
- Pregnancy, lactation or wish to conceive
- Female patients of childbearing potential not using adequate contraception
- Any contraindication to perform MRI with gadolinium-based contrast agent (including implanted stimulation devices and severe claustrophobia)

For peer review only - http://bmjopen.bmj.com/site/about/guidelines.xhtml

	STUDY PERIOD						
	Screen	Baseline	Treatment[a]				Close-out[b]
**Weeks**		-1 / 0	0	24	48	68	72
**Visits**	V_1	V_2		V_3	V_4		V_5
**ENROLMENT:**							
**Eligibility screen**	X						
**Informed consent**	X						
**Baseline characteristics[c]**	X						
**Pregnancy test, if applicable[d]**	X	X					
**^{68}Ga-DOTATATE PET/CT**	X						
**Allocation**		X					
**INTERVENTIONS:**							
**Lanreotide**			◆——	——	——	——◆	
**Placebo**			◆——	——	——	——◆	
**ASSESSMENTS:**							
**Pituitary MRI**		X[e]		X			X
**Laboratory assessment[f]**		X		X	X		X
**Physical examination[g]**		X		X	X		X
**SF-36 survey**		X		X	X		X
**Concomitant medication**		X		X	X		X
**Adverse events**		X	◆——	——	——	——◆	X[h]

For peer review only

SPIRIT
STANDARD PROTOCOL ITEMS: RECOMMENDATIONS FOR INTERVENTIONAL TRIALS

Manuscript Boertien et al. "The GALANT trial: study protocol of a randomized placebo-controlled trial in patients with a ^{68}Ga-DOTATATE PET/positive, clinically nonfunctioning pituitary macroadenoma on the effect of lanreotide on tumour size" – v 04-MAR-2020

SPIRIT 2013 Checklist: Recommended items to address in a clinical trial protocol and related documents*

Section/item	Item No	Description	Addressed in section, page no.
**Administrative information**			
Title	1	Descriptive title identifying the study design, population, interventions, and, if applicable, trial acronym	Title page, p1
Trial registration	2a	Trial identifier and registry name. If not yet registered, name of intended registry	Abstract, p2
	2b	All items from the World Health Organization Trial Registration Data Set	N/A (see registry)
Protocol version	3	Date and version identifier	Footnotes, p13
Funding	4	Sources and types of financial, material, and other support	Footnotes, p12-13
Roles and responsibilities	5a	Names, affiliations, and roles of protocol contributors	Title page, p1; Footnotes, p12
	5b	Name and contact information for the trial sponsor	Title page, p1
	5c	Role of study sponsor and funders, if any, in study design; collection, management, analysis, and interpretation of data; writing of the report; and the decision to submit the report for publication, including whether they will have ultimate authority over any of these activities	Ethics&Dissemination, p11-12; Footnotes, p12-13

For peer review only - http://bmjopen.bmj.com/site/about/guidelines.xhtml

	5d	Composition, roles, and responsibilities of the coordinating centre, steering committee, endpoint adjudication committee, data management team, and other individuals or groups overseeing the trial, if applicable (see Item 21a for data monitoring committee)	N/A
**Introduction**			
Background and rationale	6a	Description of research question and justification for undertaking the trial, including summary of relevant studies (published and unpublished) examining benefits and harms for each intervention	Introduction, p4
	6b	Explanation for choice of comparators	Intervention, p6
Objectives	7	Specific objectives or hypotheses	Introduction, p4
Trial design	8	Description of trial design including type of trial (eg, parallel group, crossover, factorial, single group), allocation ratio, and framework (eg, superiority, equivalence, noninferiority, exploratory)	Study design&setting, p5
**Methods: Participants, interventions, and outcomes**			
Study setting	9	Description of study settings (eg, community clinic, academic hospital) and list of countries where data will be collected. Reference to where list of study sites can be obtained	Study design&setting, p5
Eligibility criteria	10	Inclusion and exclusion criteria for participants. If applicable, eligibility criteria for study centres and individuals who will perform the interventions (eg, surgeons, psychotherapists)	Eligibility criteria, p5; Figure 2
Interventions	11a	Interventions for each group with sufficient detail to allow replication, including how and when they will be administered	Intervention p6; Allocation concealment…, p6-7
	11b	Criteria for discontinuing or modifying allocated interventions for a given trial participant (eg, drug dose change in response to harms, participant request, or improving/worsening disease)	Intervention, p6; Concomitant care…, p8
	11c	Strategies to improve adherence to intervention protocols, and any procedures for monitoring adherence (eg, drug tablet return, laboratory tests)	Intervention, p6
	11d	Relevant concomitant care and interventions that are permitted or prohibited during the trial	Concomitant care…, p8

For peer review only - http://bmjopen.bmj.com/site/about/guidelines.xhtml

Outcomes	12	Primary, secondary, and other outcomes, including the specific measurement variable (eg, systolic blood pressure), analysis metric (eg, change from baseline, final value, time to event), method of aggregation (eg, median, proportion), and time point for each outcome. Explanation of the clinical relevance of chosen efficacy and harm outcomes is strongly recommended	Primary outcome, p8; Secondary outcomes, p8-
Participant timeline	13	Time schedule of enrolment, interventions (including any run-ins and washouts), assessments, and visits for participants. A schematic diagram is highly recommended (see Figure)	Study outline, p5; Figure 3
Sample size	14	Estimated number of participants needed to achieve study objectives and how it was determined, including clinical and statistical assumptions supporting any sample size calculations	Sample size, p9
Recruitment	15	Strategies for achieving adequate participant enrolment to reach target sample size	Study outline, p5
**Methods: Assignment of interventions (for controlled trials)**			
Allocation:			
Sequence generation	16a	Method of generating the allocation sequence (eg, computer-generated random numbers), and list of any factors for stratification. To reduce predictability of a random sequence, details of any planned restriction (eg, blocking) should be provided in a separate document that is unavailable to those who enrol participants or assign interventions	Randomisation, p6
Allocation concealment mechanism	16b	Mechanism of implementing the allocation sequence (eg, central telephone; sequentially numbered, opaque, sealed envelopes), describing any steps to conceal the sequence until interventions are assigned	Randomisation, p6; Allocation concealment…, p6-7
Implementation	16c	Who will generate the allocation sequence, who will enrol participants, and who will assign participants to interventions	Allocation concealment…, p6-7
Blinding (masking)	17a	Who will be blinded after assignment to interventions (eg, trial participants, care providers, outcome assessors, data analysts), and how	Allocation concealment…, p6-7
	17b	If blinded, circumstances under which unblinding is permissible, and procedure for revealing a participant's allocated intervention during the trial	Allocation concealment…, p6-7
**Methods: Data collection, management, and analysis**			

Data collection methods	18a	Plans for assessment and collection of outcome, baseline, and other trial data, including any related processes to promote data quality (eg, duplicate measurements, training of assessors) and a description of study instruments (eg, questionnaires, laboratory tests) along with their reliability and validity, if known. **Reference to where data collection forms can be found, if not in the protocol**	Study outline, p5; Assessments…, p7; Outcomes, p8-9; Data management, p10-11
	18b	Plans to promote participant retention and complete follow-up, including list of any outcome data to be collected for participants who discontinue or deviate from intervention protocols	Study outline, p5; Statistical analyses, p9-10
Data management	19	Plans for data entry, coding, security, and storage, including any related processes to promote data quality (eg, double data entry; range checks for data values). **Reference to where details of data management procedures can be found, if not in the protocol**	Data management, p10-11
Statistical methods	20a	Statistical methods for analysing primary and secondary outcomes. Reference to where other details of the statistical analysis plan can be found, if not in the protocol	Statistical analyses, p9-10
	20b	Methods for any additional analyses (eg, subgroup and adjusted analyses)	Statistical analyses, p9-10
	20c	Definition of analysis population relating to protocol non-adherence (eg, as randomised analysis), and any statistical methods to handle missing data (eg, multiple imputation)	Statistical analyses, p9-10
**Methods: Monitoring**			
Data monitoring	21a	Composition of data monitoring committee (DMC); summary of its role and reporting structure; statement of whether it is independent from the sponsor and competing interests; and reference to where further details about its charter can be found, if not in the protocol. Alternatively, an explanation of why a DMC is not needed	N/A (no DMC), Monitoring, p11
	21b	Description of any interim analyses and stopping guidelines, including who will have access to these interim results and make the final decision to terminate the trial	N/A (no interim analyses)
Harms	22	Plans for collecting, assessing, reporting, and managing solicited and spontaneously reported adverse events and other unintended effects of trial interventions or trial conduct	Assessments.., p7; Safety, p11

Auditing	23	Frequency and procedures for auditing trial conduct, if any, and whether the process will be independent from investigators and the sponsor	Monitoring, p11
**Ethics and dissemination**			
Research ethics approval	24	Plans for seeking research ethics committee/institutional review board (REC/IRB) approval	Ethics&Dissemination, p11-12;
Protocol amendments	25	Plans for communicating important protocol modifications (eg, changes to eligibility criteria, outcomes, analyses) to relevant parties (eg, investigators, REC/IRBs, trial participants, trial registries, journals, regulators)	Ethics&Dissemination, p11-12;
Consent or assent	26a	Who will obtain informed consent or assent from potential trial participants or authorised surrogates, and how (see Item 32)	Study outline, p5
	26b	Additional consent provisions for collection and use of participant data and biological specimens in ancillary studies, if applicable	N/A
Confidentiality	27	How personal information about potential and enrolled participants will be collected, shared, and maintained in order to protect confidentiality before, during, and after the trial	Data management p10-11
Declaration of interests	28	Financial and other competing interests for principal investigators for the overall trial and each study site	Footnotes, p13
Access to data	29	Statement of who will have access to the final trial dataset, and disclosure of contractual agreements that limit such access for investigators	Ethics&Dissemination, p11-12; Footnotes, 13
Ancillary and post-trial care	30	Provisions, if any, for ancillary and post-trial care, and for compensation to those who suffer harm from trial participation	Concomitant care…, p8
Dissemination policy	31a	Plans for investigators and sponsor to communicate trial results to participants, healthcare professionals, the public, and other relevant groups (eg, via publication, reporting in results databases, or other data sharing arrangements), including any publication restrictions	Ethics&Dissemination, p11-12;
	31b	Authorship eligibility guidelines and any intended use of professional writers	Ethics&Dissemination, p11-12;

For peer review

	31c	Plans, if any, for granting public access to the full protocol, participant-level dataset, and statistical code	Ethics&Dissemination, p11-12;
**Appendices**			
Informed consent materials	32	Model consent form and other related documentation given to participants and authorised surrogates	Supplementary file S2 and S3
Biological specimens	33	Plans for collection, laboratory evaluation, and storage of biological specimens **for genetic or molecular analysis** in the current trial and for future use in ancillary studies, if applicable	N/A

*It is strongly recommended that this checklist be read in conjunction with the SPIRIT 2013 Explanation & Elaboration for important clarification on the items. Amendments to the protocol should be tracked and dated. The SPIRIT checklist is copyrighted by the SPIRIT Group under the Creative Commons "Attribution-NonCommercial-NoDerivs 3.0 Unported" license.

For peer review only

INFORMATIEBRIEF VOOR PROEFPERSONEN

GALANT: Het effect van lanreotide op de grootte van niet-functionerende hypofyse macroadenomen (NFMA)

Officiële titel: Een gerandomiseerde, placebo-gecontroleerde studie onder patiënten met **Ga**llium-68 DOTATATE PET/CT positieve, klinisch niet-functionerende hypofyse macroadenomen (NFMA) naar het effect van **Lan**reotide autosolution op **T**umorgrootte (**GALANT**)

Geachte heer/mevrouw,

Wij vragen u vriendelijk om mee te doen aan een medisch-wetenschappelijk onderzoek. Wij hebben u benaderd omdat u op dit moment onder behandeling bent bij de afdeling Endocrinologie van het AMC vanwege een adenoom (tumor) van de hypofyse. U beslist zelf of u wilt meedoen. Voordat u de beslissing neemt, is het belangrijk om meer te weten over het onderzoek. Lees deze informatiebrief daarom rustig door en bespreek het met partner, vrienden en/of familie. Bekijk ook de bijgevoegde brochure 'Medisch-wetenschappelijk onderzoek', daarin staat veel algemene informatie. Hebt u na het lezen van de informatie nog vragen? Dan kunt u terecht bij de arts-onderzoeker of de onafhankelijke arts. In **bijlage A**, pagina 7, vindt u de contactgegevens.

1. Wat is het doel van het onderzoek?

Het doel van dit onderzoek is het testen van het medicijn lanreotide als behandeling van bepaalde hypofyse tumoren. De hypofyse is een klein orgaan dat vlak onder de hersenen hangt, achter de neusbrug. De hypofyse heeft een centrale rol in de hormoonhuishouding van het lichaam. Een hypofyse adenoom is een goedaardige tumor of gezwel van de hypofyse. Als dit adenoom zelf geen hormonen produceert wordt dit "niet-functionerend" genoemd. Adenomen groter dan 1 centimeter worden macroadenomen genoemd. Door groei van het adenoom kan het gezonde deel van de hypofyse in verdrukking komen. Hierdoor kunnen hormonen uitvallen waarvoor aanvulling nodig is met hormoontherapie ("substitutie"). Als het adenoom te groot wordt, en daardoor op de oogzenuw drukt, is een operatie noodzakelijk. Helaas zien we dat hierna het adenoom toch vaak terug komt. Op dit moment is behandeling met een medicijn nog niet mogelijk. De Endocrinologie afdelingen van het AMC en het VU Medisch Centrum (VUmc) in Amsterdam zijn een gezamenlijk onderzoek gestart naar de werkzaamheid van het medicijn lanreotide bij patiënten met een niet-functionerend hypofyse macroadenoom. Wij hopen dat door dit medicijn het adenoom langzamer groeit of zelfs slinkt. Dit zou betekenen dat een operatie niet of pas later nodig is. Het onderzoek wordt verricht met financiële steun van Ipsen, het farmaceutische bedrijf dat lanreotide produceert.

2. Wat is lanreotide precies?

Lanreotide is een eiwit dat lijkt op het natuurlijke hormoon somatostatine. Het behoort tot de groep "antigroei hormonen". De merknaam van lanreotide is Somatuline AutoSolution. Dit zijn twee namen voor hetzelfde medicijn. Het wordt toegediend als een injectie. Lanreotide wordt al gebruikt voor de behandeling van hypofyse adenomen die groeihormoon of schildklier stimulerend hormoon aanmaken. Uit een aantal onderzoeken bleek dat ongeveer een derde van de niet-functionerende hypofyse adenomen ook gevoelig kan zijn voor lanreotide, zelfs als ze geen hormoon aanmaken. Deze onderzoeken waren echter

For peer review only - http://bmjopen.bmj.com/site/about/guidelines.xhtml

klein en er was geen vergelijking met een controlegroep. Hierdoor zijn de uitkomsten niet betrouwbaar genoeg.

3. Hoe wordt het onderzoek uitgevoerd?

Zie ook het overzicht in ***bijlage B***

Soort onderzoek

Het is een gerandomiseerd, dubbelblind, placebo-gecontroleerd onderzoek. Dit betekent dat een loting bepaalt in welke behandelgroep u komt (randomisatie). De ene groep krijgt het medicijn lanreotide en de andere groep krijgt een placebo (een “nepbehandeling”) in de vorm van een onschadelijke zoutoplossing. Beide middelen worden als injectie toegediend. Dubbelblind betekent dat u en ook de onderzoekers niet weten welke behandeling u krijgt. Er vindt eerst een screening plaats om te zien of u mee kunt doen aan de loting en de behandeling.

De eerste bezoeken: screening

Indien u interesse heeft om mee te doen aan het onderzoek volgt een uitnodiging voor het eerste bezoek in het AMC. Hier zullen we eerst bekijken of deelname mogelijk is. Allereerst zal de onderzoeker wat algemene informatie vastleggen, zoals doorgemaakte en huidige ziektes, medicatiegebruik, allergieën etc. Wij vragen u ook een vragenlijst in te vullen over uw kwaliteit van leven. Hiernaast vinden tijdens het eerste bezoek een kort lichamelijk onderzoek en bloedafname (8 buisjes, 36 milliliter bloed) plaats. De MRI-scan van de hersenen wordt herhaald als het nodig is om de precieze grootte van het adenoom opnieuw te meten. Alleen adenomen groter dan 1 centimeter kunnen onderzocht worden. De genoemde testen en controles maken deel uit van uw reguliere behandeling, met uitzondering van de vragenlijst.

Als blijkt dat deelname mogelijk is volgt er nog een tweede screening. Deze tweede screening bestaat uit een speciale PET/CT scan. Deze scan laat zien of het adenoom bepaalde eigenschappen heeft waardoor lanreotide aan het adenoom kan binden. Zo ja, dan bestaat de kans dat het adenoom reageert op een behandeling met lanreotide. Alleen in dat geval kunt u meedoen aan het onderzoek. De scan vindt plaats in het AMC. Als dat mogelijk is al op dezelfde dag, maar hier kan ook een tweede bezoek voor nodig zijn. Voor de scan krijgt u een kleine hoeveelheid van een licht radioactieve stof (een radionuclide) toegediend via een infuus. Meer informatie over de PET/CT scan vindt u in **bijlage D**.

De verdere uitvoering en duur van het onderzoek

De loting vindt plaats als na de screening blijkt dat u mee kunt doen aan het onderzoek. Hierna starten de injecties. Elke 4 weken (28 dagen) krijgt u een injectie. Deze wordt diep onder de huid van de bil toegediend. In totaal krijgt u 18 injecties van óf lanreotide óf placebo. In principe worden de injecties elke 4 weken in het AMC toegediend door een gespecialiseerde endocrinologie verpleegkundige. Als dit voor u ver reizen is of als het niet goed uitkomt om overdag naar het AMC te komen, dan kunnen de injecties ook thuis of zelfs op werk toegediend worden. Dit wordt gedaan door verpleegkundigen van de Somacare thuiszorgservice van Eurocept Homecare. U wordt als deelnemer aan deze studie bij hen aangemeld zodat zij uw gegevens hebben en contact kunnen opnemen over de toediening.

Tijdens het onderzoek komt u verder elk halfjaar naar het AMC voor een controle-bezoek aan de arts-onderzoeker, in totaal drie keer. Hier herhalen we het lichamelijk onderzoek, de bloedafname en de vragenlijst. Ook vragen we naar mogelijke bijwerkingen of andere problemen. Het eerste en tweede controle-bezoek vinden plaats op de dag van een injectie, zodat deze tegelijk in het AMC kan plaatsvinden. Het derde en laatste controle-bezoek vindt plaats ná de laatste injectie. Rond het eerste en rond het laatste

For peer review only - http://bmjopen.bmj.com/site/about/guidelines.xhtml

bezoek wordt de MRI-scan van de hersenen herhaald om de grootte van het adenoom te bekijken. De totale duur van het onderzoek is ongeveer anderhalf jaar (18 maanden). In totaal zijn er minimaal 4 en maximaal 21 bezoeken aan het ziekenhuis. Dit hangt af van waar de injecties toegediend worden. In **bijlage B** vindt u een handig overzicht van het onderzoek.

4. Wat wordt er van u verwacht?

Indien u meedoet aan het onderzoek vragen wij u vaker naar het ziekenhuis te komen dan gebruikelijk. Hoe vaak u precies moet komen hangt af van waar de injecties worden toegediend. Als u de injecties in het AMC laat toedienen dan zijn deze bezoeken van korte duur. Om het onderzoek goed te kunnen uitvoeren is het belangrijk dat u zich houdt aan de geplande afspraken in het ziekenhuis en thuis. U kunt contact opnemen met de arts-onderzoeker of met Eurocept Homecare als u een afspraak wilt wijzigen.
Het onderzoek heeft geen invloed op uw dagelijkse activiteiten of werk. Er zijn geen beperkingen wat eten en drinken betreft. Lanreotide kan soms effect hebben op andere medicijnen. Daarom is het belangrijk dat de onderzoeker voor de start van het onderzoek uw medicatie controleert. Als deelname mogelijk is mag u uw eigen medicatie blijven gebruiken. Het is wel belangrijk de onderzoeker in te lichten voordat u nieuwe medicijnen gaat gebruiken tijdens de studie.

Voor vrouwelijke deelnemers
Geeft u borstvoeding, bent u zwanger of van plan dit te worden? Dan kunt u niet meedoen aan dit onderzoek. Bent u in de vruchtbare leeftijd? Dan is het van belang om tijdens het onderzoek adequate voorbehoedsmiddelen (anticonceptie) te gebruiken. Er is namelijk nog te weinig bekend over de gevolgen van lanreotide op het ongeboren kind. Wordt u toch zwanger tijdens het onderzoek? Neem dan contact op met de onderzoeker of uw arts. De zwangerschap wordt dan extra gecontroleerd.

5. Heeft meedoen invloed op de reguliere behandeling die u krijgt?

Meedoen heeft geen invloed op de reguliere behandeling van uw niet-functionerende hypofyse adenoom. Deze bestaat gewoonlijk uit controle van klachten, de grootte van het adenoom en laboratoriumbepalingen; naast eventuele hormoontherapie om tekorten aan te vullen (“substitutie”). Deze controles en therapie vinden ook plaats tijdens het onderzoek. Bepaalde klachten kunnen passen bij groei van het adenoom. Indien deze optreden tijdens het onderzoek krijgt u, conform de reguliere behandeling, een extra MRI-scan van de hersenen. Als blijkt dat het adenoom geopereerd moet worden stopt het onderzoek. Dit heeft geen consequenties voor uw verdere behandeling.

6. Welke bijwerkingen en ongemakken kunt u verwachten?

Lanreotide
Zoals alle medicijnen kan ook lanreotide bijwerkingen veroorzaken, al heeft lang niet iedereen hier last van. Hieronder staan de meest voorkomende bijwerkingen. Deze komen bij ongeveer 10% (één op de tien) van de mensen voor:

- Maag-darmklachten zoals diarree, buikpijn en misselijkheid (vooral na de eerste injectie)
- Vorming van galstenen (meestal zonder klachten)
- Huidreacties op de injectieplaats zoals zwelling, pijn en jeuk

Andere bijwerkingen komen minder vaak voor of zijn zeldzaam. Ook bestaat er altijd een kans op nog onbekende bijwerkingen. Meer informatie over de bijwerkingen vindt u in **bijlage C**. Daarin staat ook met wie u contact moet opnemen bij een mogelijk ernstige bijwerking.

For peer review only - http://bmjopen.bmj.com/site/about/guidelines.xhtml

MRI scans (reguliere behandeling)

Een MRI-scan werkt met een magneet, hierbij wordt u niet blootgesteld aan straling. Een MRI-scan kan niet plaatsvinden als zich in of op het lichaam kleine metaaldeeltjes bevinden, zoals een metaalsplinter in het oog, implantaten in de oren of metalen vaatclips in het hoofd. Ook kan dit niet bij geïmplanteerde elektronische apparatuur, zoals een pacemaker.

Bij de MRI-scans krijgt u een contrastmiddel toegediend via een bloedvat in de arm. Dit middel bevat geen jodium. In sommige gevallen ontstaan door het contrastmiddel kortdurend onschuldige bijwerkingen zoals hoofdpijn en misselijkheid. Als u bij een eerdere MRI-scan een allergische reactie hebt gehad kunt u niet meedoen met het onderzoek.

PET/CT scan en de radionuclide

Voor de PET/CT krijgt u zoals we eerder schreven een radionuclide toegediend, deze heet "Gallium-68 DOTATATE". (Allergische) reacties hierop zijn zeldzaam. Het prikken van het infuus voor de toediening kan onprettig zijn en een blauwe plek veroorzaken.

De radionuclide is licht radioactief. Het stofje blijft net lang genoeg radioactief om de afbeeldingen te kunnen maken. Hierbij wordt u blootgesteld aan een kleine hoeveelheid straling. De geschatte stralingsbelasting van dit onderzoek is 3.1 mSv (millisievert). Uitgebreide informatie hierover vindt u in **bijlage D**.

7. Wat zijn mogelijke voor- en nadelen van deelname aan dit onderzoek?

Door resultaten uit eerder onderzoek is de verwachting dat lanreotide de groei van bepaalde hypofyse adenomen remt, maar zeker is dat niet. Het is daarom niet te zeggen of u voordeel heeft van deelname aan het onderzoek. Het huidige onderzoek zal het effect van lanreotide nauwkeuriger bestuderen en het vergelijken met een controlegroep. De kans om deel van die controlegroep te zijn en placebo injecties te krijgen is 50% (één op twee). Dit is een nadeel, ook vanwege de vele afspraken thuis of bezoeken aan het ziekenhuis om de injecties te ontvangen. Andere nadelen zijn de extra PET/CT scan met de bijbehorende straling en de mogelijke bijwerkingen of ongemakken van de injecties zelf. Tijdens de onderzoeksperiode wordt 4 keer een kleine hoeveelheid bloed afgenomen (telkens 36 milliliter). Dit gebeurt gewoonlijk ook tijdens de reguliere behandeling. Als er onverwacht afwijkingen uit het bloedonderzoek of de scans komen, zullen we dit altijd met u bespreken. Als u dit niet wilt, kunt u niet aan het onderzoek meedoen.

8. Wat gebeurt er als u niet wenst deel te nemen aan dit onderzoek of wilt stoppen?

Deelname aan het onderzoek is geheel vrijwillig. Als u besluit niet mee te doen, hoeft u niet te zeggen waarom. Als u wel meedoet, kunt u zich altijd bedenken en toch stoppen, ook tijdens het onderzoek. Als u besluit niet mee te doen of te stoppen, dan worden uw eigen behandeling en controles gewoon voortgezet. Tussentijds stoppen heeft geen risico's voor uw gezondheid. Deelname kan ook door de onderzoeker worden beëindigd. Bijvoorbeeld als uw veiligheid in gevaar is.

Als er tijdens het onderzoek nieuwe informatie naar voren komt die belangrijk is voor u, laat de onderzoeker dit altijd aan u weten. U mag dan zelf beslissen of u blijft meedoen. Extra informatie over deelname, stoppen en uw rechten als proefpersoon kunt u vinden in de bijgevoegde brochure.

For peer review only - http://bmjopen.bmj.com/site/about/guidelines.xhtml

9. Wat gebeurt er met uw gegevens?

Privacy

Voor dit onderzoek worden uw medische en persoonlijke gegevens op verschillende manieren verzameld en gebruikt. Al uw gegevens blijven vertrouwelijk.

Hieronder staat waar en waarom uw persoonsgegevens verzameld worden:

- Bij de thuiszorgservice van Eurocept Homecare worden uw persoonsgegevens geregistreerd voor het inplannen en toedienen van injecties thuis.
- In een apart studiedossier in het AMC slaat de arts-onderzoeker uw contactgegevens op zodat zij u kan bereiken als dat nodig is.

Hiernaast worden alle gegevens voor het onderzoek verzameld en opgeslagen met behulp van een studiecode. Elke proefpersoon krijgt een eigen studiecode die de persoonsgegevens zoals uw naam vervangt. Welke code u heeft is alleen bekend bij het onderzoeksteam. Bij bespreken van en in rapporten over het onderzoek wordt alleen de studiecode gebruikt.

Inzien van onderzoeksgegevens

Om te controleren of het onderzoek degelijk en betrouwbaar wordt uitgevoerd, mogen bepaalde mensen wel uw gegevens zonder code inzien. Naast het onderzoeksteam zijn dit een controleur van het AMC en de Inspectie voor de Gezondheidszorg. Zij zijn verplicht om uw gegevens geheim te houden. Als vertegenwoordigers van Ipsen, de financier van het onderzoek, het onderzoek moeten controleren dan mogen zij de gegevens alleen met de studiecode inzien.

Bewaren van gegevens

Alle onderzoeksgegevens moeten wettelijk 15 jaar bewaard worden. Bij het ondertekenen van het toestemmingsformulier geeft u toestemming voor het verzamelen, inzien en bewaren van uw medische en persoonsgegevens. Algemene informatie over omgang met gegevens vindt u ook in de bijgevoegde brochure.

Bewaren van bloed

Gedurende de onderzoeksperiode wordt als reguliere controle een paar keer bloed afgenomen. Een deel hiervan word langer bewaard dan normaal, ook met gebruik van de code. Dit gebeurt voor het geval er later aanvullende bepalingen nodig blijken. Het bewaarde bloed wordt alleen voor het huidige onderzoek gebruikt. Het wordt vernietigd zodra het onderzoek in zijn geheel is voltooid. Dit houdt in dat alle patiënten die meedoen het onderzoek hebben afgerond en dat de resultaten kunnen worden gepubliceerd. In totaal zal het niet langer dan 5 jaar bewaard worden. Voor het bewaren van uw bloed geeft u toestemming via het toestemmingsformulier.

10. Bent u verzekerd wanneer u aan het onderzoek meedoet?

Voor iedereen die meedoet aan dit onderzoek is een verzekering afgesloten. De verzekering dekt schade als gevolg van het onderzoek. Dit geldt voor schade die naar boven komt tijdens het onderzoek, of binnen vier jaar na het einde van het onderzoek. In **bijlage E** vindt u de verzekerde bedragen, de uitzonderingen en de contactgegevens van de verzekeraar.

For peer review only - http://bmjopen.bmj.com/site/about/guidelines.xhtml

11. Wordt uw huisarts en/of behandelend specialist geïnformeerd bij deelname?

Wij laten uw huisarts en uw behandelend internist-endocrinoloog altijd schriftelijk weten dat u meedoet aan het onderzoek. Hiervoor geeft u toestemming via het toestemmingsformulier. Als u geen toestemming geeft, is meedoen niet mogelijk.

12. Is er een vergoeding wanneer u besluit deel te nemen?

De studiemedicatie, extra bezoeken en PET/CT scan kosten u niks. U wordt niet betaald voor het meedoen aan dit onderzoek. Wel krijgt u een vergoeding voor uw reiskosten.

13. Welke medisch-ethische toetsingscommissie heeft dit onderzoek goedgekeurd?

De medisch-ethische toetsingscommissie (METC) van het AMC heeft dit onderzoek goedgekeurd. Meer informatie over goedkeuring vindt u in de algemene brochure.

14. Wilt u verder nog iets weten?

U hoeft niet direct te beslissen of u mee wilt doen. De bedenktijd is ongeveer een week. Bij vragen kunt u contact opnemen met de arts-onderzoeker, Tessel Boertien. Voor onafhankelijk advies over meedoen aan dit onderzoek kunt u terecht bij de onafhankelijke arts, dr. M.R. Soeters, internist-endocrinoloog. Hij weet van het onderzoek af maar is zelf niet betrokken. Naast de algemene brochure kunt u ook op de website van het AMC veel informatie vinden, onder andere over het meedoen aan onderzoek en rechten & plichten als patiënt. Alle contactgegevens vindt u in **bijlage A**.

Dank voor uw aandacht en met vriendelijke groet,

Drs. Tessel M. Boertien, arts-onderzoeker en projectleider
Prof. Dr. E. Fliers, internist-endocrinoloog en hoofdonderzoeker AMC

<u>Bijlagen bij deze informatiebrief</u>:

- Los: Brochure 'Medisch-wetenschappelijk onderzoek. Algemene informatie voor de proefpersoon' (versie september 2014)
- Bijlage A: Contactgegevens en informatie per centrum
- Bijlage B: Overzicht van het onderzoek
- Bijlage C: Bijwerkingen lanreotide – Somatuline AutoSolution
- Bijlage D: Extra informatie over PET/CT en stralenbelasting
- Bijlage E: Verzekeringstekst
- Bijlage F: Toestemmingsformulier

For peer review only - http://bmjopen.bmj.com/site/about/guidelines.xhtml

GALANT studie
NL52821.018.15

Amsterdam UMC
Universitair Medische Centra

Bijlage A: Contactgegevens en informatie per centrum

Projectleider & eerste contactpersoon

Drs. Tessel M. Boertien, arts-onderzoeker endocrinologie AMC & VUmc
e-mail **t.m.boertien@amsterdamumc.nl**
Tel. **020 566 0172** / 020 566 6071 (secr.)

Postadres onderzoek: Academisch Medisch Centrum, t.a.v. T.M. Boertien
Afdeling Endocrinologie & Metabolisme, kamer K2-283
Meibergdreef 9, 1105 AZ Amsterdam

Onafhankelijk arts AMC

Dr. M.R. Soeters, internist-endocrinoloog
e-mail m.r.soeters@amsterdamumc.nl
Tel. 020 566 6071

Informatie Academisch Medisch Centrum (AMC)

Bezoekadres: Meibergdreef 9
1105 AZ Amsterdam
Algemeen nummer: 020 566 9111
Nucleaire geneeskunde: 020 566 2775 (7:30-17:00)

Bij acute problemen buiten kantoortijden kunt u via het algemene telefoonnummer (020 566 9111) 24 uur per dag worden doorverbonden naar de dienstdoende internist-endocrinoloog. Vermeld altijd dat u meedoet aan deze studie.

Hoofdonderzoeker

Prof. dr. E. Fliers, internist-endocrinoloog
e-mail e.fliers@amsterdamumc.nl
Tel. 020 566 6071

Endocrinologie verpleegkundigen

Moniek Gent-Houben en Shariefa Peters-Amanatkhan
e-mail fk.endocrinologie@amsterdamumc.nl
Tel. 020 566 2990

Afdeling Patiëntenvoorlichting en Klachtenopvang AMC

Bij de afdeling patiëntenvoorlichting en klachtenopvang in het AMC kunt u terecht met algemene vragen en informatie, bijvoorbeeld over het meedoen aan onderzoek. Ook kunt u hier terecht indien u ontevreden bent over de gang van zaken of een klacht wilt indienen. De medewerkers verzorgen de eerste klachtenopvang en informeren over de klachtenprocedure. Op de website van het AMC is veel van de informatie terug te vinden: https://www.amc.nl/web/ik-heb-een-afspraak-1/rechten-en-plichten.htm

Locatie binnen AMC: begane grond van het polikliniekgebouw (A0)
Openingstijden: maandag t/m vrijdag van 9:00 tot 12:30 uur

For peer review only - http://bmjopen.bmj.com/site/about/guidelines.xhtml
For peer review only

Tel.:	020 566 3355
e-mail:	patientenvoorlichting@amc.nl
	klachtenfunctionaris@amc.nl
Postadres:	Academisch Medisch Centrum, t.a.v. patiëntenvoorlichting, A0-144
	Postbus 22660, 1100 DD Amsterdam

Contactgegevens Eurocept Homecare

Tel.: 035 528 8375 (ook buiten kantooruren)

e-mail galantstudie@eurocept.nl

For peer review only

For peer review only - http://bmjopen.bmj.com/site/about/guidelines.xhtml

BIJLAGE B: OVERZICHT VAN HET ONDERZOEK

Hieronder staat een schematisch overzicht van het onderzoek. De studie bezoeken met de **dikgedrukte** nummers vinden in ieder geval plaats. In dit voorbeeld overzicht staat de PET/CT scan gepland tijdens een apart bezoek (nr. 2). Het kan zijn dat deze scan al tijdens bezoek nr. 1 plaatsvindt. De 7e en 13e injectie vinden plaats op de dag dat u ook naar het AMC komt. Het totale aantal studie bezoeken hangt af van waar de overige injecties plaatsvinden: thuis of in het AMC. Daarom staan die bezoeknummers tussen haakjes. Als alle injecties in het AMC plaatsvinden zijn er in totaal 21 bezoeken.

LOTING ↓ (tussen bezoek 2 en bezoek (3 – 8))

Tijd → / Studie bezoek →	start 1	2	(3 – 8)	6 mnd. 3 (9)	(10 – 14)	12 mnd. 4 (15)	(16 – 20)	18 mnd. 5 (21)	EINDE
Locatie →	AMC	AMC	AMC/thuis	AMC	AMC/thuis	AMC	AMC/thuis	AMC	
Duur in minuten →	90	90	15	90	15	60	15	75	

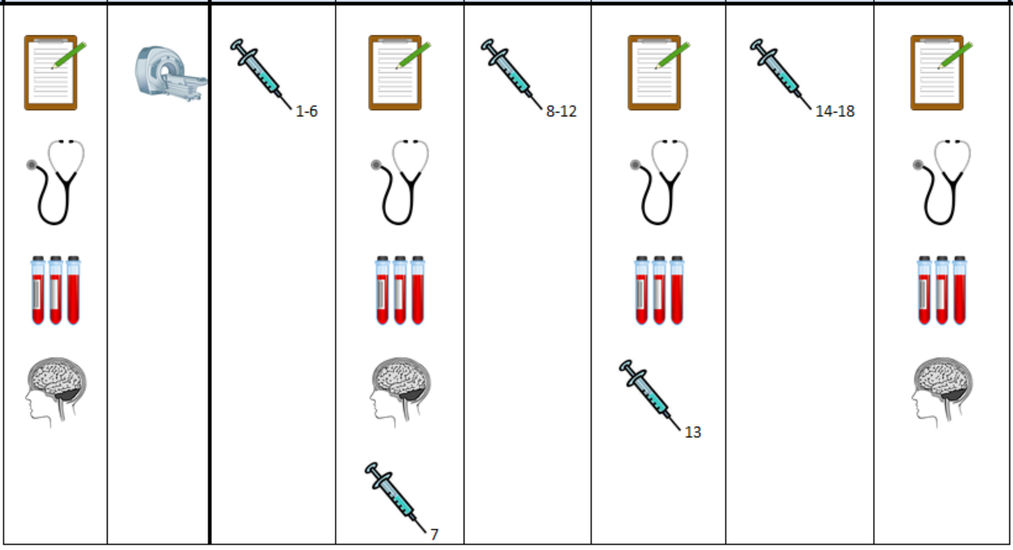

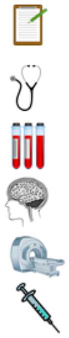

= gesprek en kwaliteit van leven vragenlijst

= kort lichamelijk onderzoek (bloeddruk, pols, lengte, gewicht; uitgebreider op geleide van klachten)

= bloed afname (één prik, 8 buisjes, 36 milliliter)

= MRI-hersenen

= PET/CT scan

= subcutane injectie (lanreotide / Somatuline AutoSolution óf placebo)

For peer review only - http://bmjopen.bmj.com/site/about/guidelines.xhtml

GALANT studie
NL52821.018.15

BIJLAGE C: BIJWERKINGEN LANREOTIDE – SOMATULINE AUTOSOLUTION

Zoals alle geneesmiddelen kan Somatuline AutoSolution, de merknaam van lanreotide, bijwerkingen veroorzaken. Lang niet iedereen krijgt hier last van.

De meest voorkomende bijwerkingen zijn zoals eerder genoemd maag-darmklachten, galblaasproblemen en reacties op de injectieplaats. Hieronder staat een lijst van de bijwerkingen die het vaakst optreden met Somatuline AutoSolution:

<u>Zeer vaak: komen voor bij meer dan 1 op de 10 gebruikers (>10%)</u>
- Diarree of dunne ontlasting, buikpijn
- Vorming van galstenen; dit veroorzaakt meestal geen klachten. Soms kan een galsteen echter leiden tot een verstopping van de galwegen. Dit kan de volgende klachten veroorzaken: ernstige en plotselinge buikpijn (vaak rechtsboven in de buik), hoge koorts, koude rillingen, geelzucht (het geel worden van de huid en het oogwit), verlies van eetlust, een jeukende huid

<u>Vaak: komen voor bij maximaal 1 op de 10 gebruikers (1-10%)</u>
- Reacties op de injectieplaats zoals zwelling, pijn en jeuk
- Misselijkheid, braken, constipatie (verstopping), winderigheid, opgezette buik, vreemd of opgeblazen gevoel in de buik, dyspepsie (gestoorde spijsvertering/maagklachten), vettige ontlasting
- Verminderde eetlust
- Gewichtsverlies
- Duizeligheid, hoofdpijn
- Pijn in de spieren, gewrichtsbanden, pezen of botten
- Vermoeidheid, gebrek aan energie of slaperigheid
- Trage hartslag
- Haaruitval of minder lichaamsbeharing
- Verwijding van de galwegen
- Verlaagde bloedsuikerspiegel: dit kan klachten geven van overmatig zweten, trillen, hongergevoel en verwardheid
- Verhoogde bloedsuikerspiegel: dit kan klachten geven van dorst, droge mond en vermoeidheid
- Afwijkende lever- en alveesklierwaarden, zoals blijkt uit bloedtesten

<u>Soms: komen voor bij maximaal 1 op de 100 gebruikers (<1%)</u>
- Opvliegers
- Slaapproblemen
- Verandering van de kleur van de ontlasting
- Veranderingen in de concentratie natrium en alkalinefosfaat, zoals blijkt uit bloedtesten

Wanneer u een ernstige bijwerking vermoedt of als u een andere ernstige klacht krijgt die hier niet tussen staat, raadpleeg dan de arts-onderzoeker! Indien geen gehoor of buiten kantooruren, raadpleeg dan de dienstdoende internist(-endocrinoloog) van uw ziekenhuis - *zie **bijlage A** voor contactgegevens*

For peer review only - http://bmjopen.bmj.com/site/about/guidelines.xhtml

BIJLAGE D: EXTRA INFORMATIE OVER PET/CT

Algemene informatie
De PET/CT scan wordt in het AMC uitgevoerd op de afdeling Nucleaire Geneeskunde. Deze vindt óf in de middag van het eerste bezoek plaats óf op een apart, tweede bezoek. U hoeft voor de scan niet nuchter te zijn. Op de afdeling Nucleaire Geneeskunde krijgt u een infuusnaald geplaatst in één arm. Via dit infuus krijgt u een kleine hoeveelheid van de licht radioactieve stof toegediend (de radionuclide Gallium-68 DOTATATE). Hierna wordt het infuus verwijderd. Het duurt 45 tot 60 minuten voordat deze stof goed is opgenomen door het lichaam en de hypofyse. Gedurende deze wachttijd mag u in de wachtkamer plaatsnemen, of iets gaan eten of drinken. Hierna neemt u rustig plaats op het bed van de PET/CT-scanner. Het maken van de scan duurt ongeveer 10 minuten, waarbij u op het bed door de brede ring van de scanner wordt geschoven. Deze ring is aan de voor en achterkant open. De laborant is constant aanwezig, en u kunt via de intercom contact met elkaar houden. Belangrijk is dat u tijdens het gehele onderzoek zo stil mogelijk blijft liggen. Na het maken van de scan mag u meteen naar huis. Het toedienen van de radionuclide heeft geen risico voor de omgeving. U mag na de scan meteen alles doen wat u normaal ook zou doen.

Stralingsbelasting
De stralingsbelasting wordt uitgedrukt in mSv (millisievert). De geschatte stralingsbelasting in dit onderzoek is 3.1 mSv. Ter vergelijking: de achtergrondstraling in Nederland is 2-2,5 mSv per jaar. Als u vaker meedoet aan wetenschappelijk onderzoek met stralingsbelasting, moet u er op letten dat de totale hoeveelheid over 5 jaar niet meer is dan 10 mSv. Ook kunt u beter niet mee doen aan dit onderzoek als u in uw beroep meer straling ontvangt dan de achtergrondstraling.

Wat is het risico van straling?
Het lichaam bestaat uit verschillende cellen. Wij hebben bijvoorbeeld hersencellen, spiercellen, bloedcellen, etc. Het zijn de genen (het erfelijke materiaal) in de cel die bepalen hoe de cel functioneert. Röntgenstraling of straling van radioactieve stoffen kan genen beschadigen. Deze beschadiging kan leiden tot kanker of een aangeboren afwijking.

Hoe groot zijn de kansen op deze ernstige gevolgen?
Een blootstelling aan 1 mSv straling geeft een kans van 1 op 17.000 op kanker. Bij een stralingsbelasting van 5 mSv is deze kans 1 op 3.400 en bij 10 mSv 1 op 1.700. Kanker wordt meestal pas 20 jaar of nog later ná de blootstelling aan de straling vastgesteld. De genoemde kansen moet u vergelijken met de kans op kanker van 25% (1 op 4) die elk mens heeft.
Als genen in zaadcellen of eicellen van de ouder beschadigd raken, bestaat het risico op een aangeboren afwijking van het kind. Een dosis van 1 mSv straling zal bij 1 op de 77.000 baby's (van een ouder die deze stralingsdosis kreeg) de oorzaak zijn van een ernstige aangeboren afwijking. Deze kans moet u vergelijken met de kans van 2,3% (23 op de 1000) op een aangeboren afwijking die elke baby heeft.

For peer review only - http://bmjopen.bmj.com/site/about/guidelines.xhtml

BIJLAGE E: VERZEKERINGSTEKST

Overeenkomstig de Wet medisch-wetenschappelijk onderzoek met mensen heeft de AMC Medical Research B.V. voor medisch-wetenschappelijk onderzoek een verzekering afgesloten die door het onderzoek veroorzaakte schade door dood of letsel van de proefpersoon dekt. Dit betreft zowel schade die zich tijdens de duur van het onderzoek openbaart als schade die zich openbaart, en gemeld is, binnen vier jaar na beëindiging van de deelname aan het onderzoek.
De verzekering biedt een dekking van €450.000 per proefpersoon met een maximum van €3.500.000 voor het hele onderzoek en een maximum van €5.000.000 per jaar voor alle onderzoeken van dezelfde opdrachtgever.

De verzekering biedt dekking:

- voor schade ten gevolge van de verwezenlijking van de deelname aan het wetenschappelijk onderzoek verbonden risico's waarover men niet schriftelijk is ingelicht;
- voor schade ten gevolge van de verwezenlijking van de risico's waarover de deelnemer wel is ingelicht, maar die zich in ernstiger mate voordoet dan is voorzien;
- voor schade ten gevolge van de verwezenlijking van de risico's waarover de deelnemer wel is ingelicht, maar die zeer onwaarschijnlijk werd geacht.

De verzekering biedt geen dekking:

- voor schade die het gevolg is van het uitblijven van een vermindering van de gezondheidsproblemen van de proefpersoon, dan wel het gevolg is van de verdere verslechtering van de gezondheidsproblemen, indien de deelname aan het wetenschappelijk onderzoek plaatsvindt in het kader van de behandeling van deze gezondheidsproblemen;
- voor schade door aantasting van de gezondheid van de proefpersoon waarvan aannemelijk is dat deze zich ook zou hebben geopenbaard wanneer de proefpersoon niet aan het onderzoek had deelgenomen;
- voor schade ten gevolge van deelname aan medisch-wetenschappelijk onderzoek waarbij in de kring van beroepsgenoten gebruikelijke handelingen op het gebied van de geneeskunst met elkaar worden vergeleken en aannemelijk is dat de schade het gevolg is van de toegepaste handelingen;
- voor schade die zich bij een nakomeling van de proefpersoon openbaart als gevolg van een nadelige inwerking van het onderzoek op de proefpersoon of de nakomeling;
- voor schade die het gevolg is van het niet of niet volledig opvolgen van aanwijzingen en instructies door de proefpersoon, indien de proefpersoon daartoe althans in staat is.

De verzekering dekt uitsluitend de schade van natuurlijke personen. De dekking van specifieke schades en kosten is tot bepaalde bedragen beperkt. Om aanspraak te kunnen maken op schadevergoeding dient de proefpersoon in geval van vermeende schade als gevolg van het onderzoek dit te melden aan:

Naam verzekeraar:	Centramed B.A.
Postadres:	Postbus 7374<br>2701 AJ Zoetermeer
Telefoonnummer:	070 301 7070
E-mail:	info@centramed.nl
Polisnummer:	620.872.806

Hiernaast wordt de proefpersoon verzocht contact op te nemen met de hoofdonderzoeker van het onderzoek, prof. dr. E. Fliers (Tel. 020 566 6071).

For peer review only - http://bmjopen.bmj.com/site/about/guidelines.xhtml

BIJLAGE F: TOESTEMMINGSFORMULIER

GALANT: Het effect van lanreotide op de grootte van niet-functionerende hypofyse macroadenomen (NFMA)

ABR: NL52821.018.15 – protocol versie 3.0, oktober 2016

Officiële titel: Een gerandomiseerde, placebo-gecontroleerde studie onder patiënten met **Ga**llium-68 DOTATATE PET/CT positieve, klinisch niet-functionerende hypofyse macroadenomen (NFMA) naar het effect van **Lan**reotide autosolution op **T**umorgrootte (**GALANT**)

Ik heb de informatiebrief voor de proefpersoon gelezen. Ik kon aanvullende vragen stellen. Mijn vragen zijn genoeg beantwoord. Ik had genoeg tijd om te beslissen of ik meedoe.

Ik weet dat meedoen helemaal vrijwillig is. Ik weet dat ik op ieder moment kan beslissen om toch niet mee te doen. Daarvoor hoef ik geen reden te geven.

Ik weet dat onverwachte afwijkingen die tijdens het onderzoek naar voren komen altijd met mij besproken zullen worden.

Ik geef toestemming om mijn huisarts en behandelend internist-endocrinoloog te informeren dat ik meedoe aan dit onderzoek.

Ik weet dat mijn persoonsgegevens worden geregistreerd bij Eurocept Homecare als de injecties thuis plaatsvinden.

Ik weet dat mijn contactgegevens in een studiedossier in het AMC worden opgeslagen zodat de arts-onderzoeker met mij contact kan onderhouden.

Ik weet dat het onderzoeksteam, de controleur van het AMC en vertegenwoordigers van de Inspectie voor de Gezondheidszorg mijn onderzoeksgegevens zonder code kunnen inzien.

Ik geef toestemming om mijn gegevens te verzamelen en te gebruiken voor de doelen die in de informatiebrief staan.

Ik geef toestemming om mijn onderzoeksgegevens 15 jaar na afloop van dit onderzoek te bewaren.

Ik geef toestemming om het afgenomen bloed gedurende het onderzoek te bewaren tot publicatie van de resultaten (korter dan 5 jaar), waarna het wordt vernietigd.

Ik geef ☐ **wel**
☐ **geen**
toestemming om in de toekomst benaderd te worden voor een vervolgonderzoek.

For peer review only

For peer review only - http://bmjopen.bmj.com/site/about/guidelines.xhtml

GALANT studie
NL52821.018.15

Ik wil meedoen aan dit onderzoek.

Naam proefpersoon: ……………………………………………………………………………

Handtekening: ………………………………………………………. **Datum:** ………/………./……….

Ik verklaar hierbij dat ik deze proefpersoon volledig heb geïnformeerd over het genoemde onderzoek.

Als er tijdens het onderzoek informatie bekend wordt die de toestemming van de proefpersoon zou kunnen beïnvloeden, dan breng ik hem/haar daarvan tijdig op de hoogte.

Naam onderzoeker: ……………………………………………………………………………

Handtekening: ………………………………………………………. **Datum:** ………/………./……….

Aanvullende informatie is gegeven door (indien van toepassing):

Naam: ……………………………………………………………………………………………

Functie: …………………………………………………………………………………………

Handtekening: ………………………………………………………. **Datum:** ………/………./……….

De proefpersoon krijgt een volledige informatiebrief mee, samen met een kopie van het getekende toestemmingsformulier

For peer review only

For peer review only - http://bmjopen.bmj.com/site/about/guidelines.xhtml

Amsterdam UMC
Universitair Medische Centra

SUBJECT INFORMATION FOR PARTICIPATION IN MEDICAL RESEARCH

GALANT: The effect of lanreotide on non-functioning pituitary macroadenoma (NFMA) size

Official study title: A randomized placebo-controlled study in patients with a **Ga**llium-68 DOTATATE PET/CT positive, clinically non-functioning pituitary macroadenoma (NFMA) of the effect of **Lan**reotide autosolution on **T**umor (adenoma) size (**GALANT**)

Dear Sir/Madam,

You are kindly requested to take part in a medical-scientific study. We have contacted you because you receive treatment for an adenoma (tumor) of the pituitary gland at the department of Endocrinology of the Academic Medical Center (AMC). Participation is voluntary. Participation requires your written consent. Before you decide whether you want to participate in this study, you will be given an explanation about what the study involves. Please read this information carefully and discuss it with your partner, friends and/or family. General information about participating in a study can be found in the enclosed general brochure on medical research. If you have any questions you can contact the (coordinating) investigator or the independent expert for an explanation. Contact details can be found in **appendix A**, page 7.

1. What is the purpose of the study?

The purpose of this study is to investigate the effect of the medicine lanreotide in the treatment of certain pituitary adenomas. The pituitary gland, or hypophysis, is a small but important endocrine organ. It sits at the base of the brain and is about the size and shape of a bean. It has a central role in the regulation of various hormones. Pituitary adenomas are benign tumors within the pituitary gland. Some adenomas produce excess hormone. If this is not the case the adenoma is called “non-functioning”. If the size exceeds 1 centimeter, it is called a macroadenoma. The growing adenoma can cause pressure on the pituitary gland and disrupt its function. This requires treatment to replace the missing hormones (“substitution therapy”). The growing adenoma can also cause pressure on the optic nerve, which leads to vision disturbances. If this happens, a surgical operation is necessary. Sadly, however, the adenoma grows back in most cases. At this time, no medicinal treatment is available. The departments of Endocrinology of the AMC and the VU Medical Center (VUmc) in Amsterdam have therefore started a study to examine the effect of the medicine lanreotide on the size of non-functioning pituitary macroadenoma. We hope that this medicine can slow the growth of the adenoma or even decrease its size. This could prevent or postpone surgery. The study is realized with the financial support of Ipsen, the pharmaceutical company that produces lanreotide.

2. What exactly is lanreotide?

Lanreotide is a medicinal protein that is similar to the naturally occurring hormone somatostatin. This hormone blocks the release of several other hormones such as growth hormone. The brand name of lanreotide in the Netherlands is Somatuline AutoSolution. Both names are used for the same drug. It is administered through an injection. Lanreotide is already used in the treatment of pituitary adenomas that produce growth hormone or thyroid stimulating hormone. Earlier research has shown that about one-third of non-functioning adenomas might also be susceptible to lanreotide and could benefit from treatment.

For peer review only - http://bmjopen.bmj.com/site/about/guidelines.xhtml

However, these studies were small and lacked comparison with a control group. Therefore the results are not reliable enough.

3. What does participation involve?

*Also see the study overview in **appendix B***

Study design

This is a randomized, double-blind and placebo-controlled study. Randomization means that chance decides which patient gets which study treatment. There are two treatment groups. One group will receive the medicine lanreotide and the other group will receive a placebo or "sham treatment", in this case a harmless saline solution. Both treatments are administered as an injection. Every patient will also receive their regular care. Double-blind means that neither the patient nor the investigators know which patient is getting which treatment. First, a screening will take place to see if you can participate in the randomization and treatment.

Screening: the first visits

If you are interested in participating in the study you will be invited for the first visit at the AMC. We will first evaluate whether you may participate. The investigator will register some general information about your medical history, medication use, allergies etc. We will also request you to fill in a questionnaire about your health and daily activities. Furthermore the first visit will involve a short physical examination and blood tests (8 tubes, 36 milliliters). The MRI-scan of the brain will be repeated if it is necessary to measure the size of the adenoma again. Only adenomas larger than 1 centimeter can be examined in this study. Apart from the questionnaire, the tests are part of your regular care.

If the first screening is okay, a second screening will follow. This screening consists of a specific PET/CT scan. This scan shows if the adenoma has certain characteristics that could make it susceptible for lanreotide. If so, treatment with lanreotide might be effective. Only in that case participation in the study is possible. The PET/CT takes place at the AMC. If possible on the same day of the first screening, but a second visit could be necessary. Before the scan is performed, you will receive a small amount of a slightly radioactive marker (called a radionuclide) through an intravenous injection. You can find more information about this scan in **appendix D**.

The treatment, further visits and duration of the study

Randomization takes place when the screening shows that participation is possible. Then the treatment with the injections starts. Every 4 weeks (28 days) you will receive one injection, administered in the skin of the buttock ('subcutaneously'). In total you will receive 18 injections of either lanreotide or placebo. A specialized endocrine nurse will administer the injections at the AMC. If travelling to the AMC every 4 weeks is inconvenient for you, the injections can also be administered at home or even at work. Somacare nurses from Eurocept Homecare will perform this. They will register you as a participant in this study so they can contact you about the administration.

Every six months there will be a control visit to the investigator at the AMC, three times during the study. At these visits we will repeat the physical examination, blood tests and the questionnaire. We will also ask about possible side effects or other problems. The first and second control visit will take place on the day of a planned injection, so these can take place at the same time. The third and final control visit will take place after the last injection. Around the first and the last visit the MRI scan of the brain will be repeated to check the size of the adenoma. The duration of the study is about 18 months. In total there will be a minimum of

For peer review only - http://bmjopen.bmj.com/site/about/guidelines.xhtml

4 and a maximum of 21 visits to the hospital, depending on where the injections are administered. In **appendix B** a useful overview of the study is shown.

4. What is expected of you?

If you decide to participate in the study we will ask you to come to the hospital more often than usual. The amount of visits depends on where the injections are administered. If the injections are administered at the AMC these visits will be short. In order to carry out the study properly it is important to keep to the planned appointments in the hospital and at home. If you need to reschedule you can contact the investigator or Eurocept Homecare. Participation will have no influence on your daily activities. There are no restrictions on eating or drinking. Lanreotide can have some effect on other medicines. That is why it is important to check your medication use before the start of the study. If participation is possible you can continue all of your usual medication. We will discuss it with you if extra monitoring is necessary. It is important to contact the investigator before starting to use any new medicines during the study.

For female participants

If you are breastfeeding, pregnant, or planning to become pregnant, you cannot participate in this study. For fertile women it is important to use adequate contraception. This is because there is still not enough data on the effects of lanreotide on the unborn child. If you do, however, become pregnant during the study, please contact the investigator. The pregnancy will then be monitored more closely.

5. Will participation affect your regular treatment?

Participation will not affect your regular treatment of the pituitary adenoma. Regular treatment consists of monitoring complaints, regular blood tests and checking the size of the adenoma; next to the substitution of missing hormones. This treatment is continued during the study. Certain complaints can indicate increasing size of the adenoma. If this occurs during the study, you will undergo an extra MRI-scan. If the scan shows that surgery is needed to remove the adenoma, participation in the study will stop. Withdrawal from the study will not affect your regular treatment.

6. Can you expect any side effects or discomfort?

Lanreotide

Like all medicines lanreotide may have side effects. The most common side effects are listed below. These occur in about one in ten people (10%):

- Gastrointestinal complaints such as diarrhea, abdominal pain or nausea (especially after the first injection)
- Formation of gallstones (mostly without any symptoms)
- Skin reactions where the injection is given such as swelling, pain or itching

Other known side effects are less common or rare. The drug may also have side effects that are still unknown. Please see **appendix C** for a more extensive list of possible side effects and information on who to contact if any side effect becomes severe.

MRI scans (regular care)

An MRI scan is performed using a magnet, this doesn't involve any exposure to radiation. Because of the powerful magnet an MRI scan cannot be performed if there are small parts of metal in the body, such as

For peer review only - http://bmjopen.bmj.com/site/about/guidelines.xhtml

metal fragments in the eye or certain implants. The magnet can also interfere with implanted electronic devices such as a pacemaker.

To perform the MRI scan a contrast agent is administered through an intravenous injection. During the injection you may shortly experience harmless side effects like headache and nausea. If you had an allergic reaction during earlier MRI scans you cannot participate in this study.

PET/CT scan and the radionuclide

The earlier mentioned radionuclide used to perform the PET/CT is called “Gallium-68 DOTATATE”. (Allergic) reactions to this radionuclide are rare. The injection to administer the radionuclide can be bothersome and may cause some bruising.

The radionuclide is slightly radioactive. The marker stays radioactive just long enough to perform the scan and generate the images. During this time you will be exposed to a small amount of radiation, estimated 3.1 mSv (millisievert). More extensive information on radiation exposure can be found in **appendix D**.

7. What are the possible advantages and disadvantages of participation?

The results of earlier research suggest that lanreotide may reduce the growth of non-functioning pituitary adenoma, but this is not certain. We can therefore not predict whether you will benefit from participation in this study. The current study will examine the effect of lanreotide more accurately and compare it with a control group. The chance to be part of the control group and receive placebo injections is 50% (one in two). This can be a disadvantage, also because of the frequent appointments at home or at the hospital to receive the injections. Further disadvantages are the additional PET/CT scan with the exposure to radiation and the possible discomfort and side effects of the injections. Blood tests are performed 4 times during the study period (each time 8 tubes and 36 milliliters). This is part of your regular care. If the blood tests or the scans lead to incidental findings about your health we will discuss these with you. If you do not want to be informed about these findings you cannot participate in the study.

8. What if you do not wish to participate or wish to stop participating?

It is up to you to decide whether or not to participate in the study. Participation is voluntary. If you do not want to participate, you will continue your usual treatment as before.
If you do participate in the study, you can always change your mind. You may stop participation at any time during the study. This is not harmful and will not affect your usual treatment. You do not have to say why you are stopping, but you do need to tell the investigator immediately. Participation may also be stopped by the investigator if it is considered best for you.

If there is any new information about the study that is important for you, the investigator will let you know. You will then be asked whether you still want to continue your participation. The data collected until that time can still be used for the study. Additional information about participation and your rights can be found in the general brochure.

9. How will your data be handled?

Privacy

For this study your medical and personal data will be collected and used in several ways. All your data will remain confidential.

For peer review only - http://bmjopen.bmj.com/site/about/guidelines.xhtml

Here we list where and why your personal data will be collected:

- At Eurocept Homecare your personal data will be registered in order to plan and administer injections at home.
- At the AMC the coordinating investigator will store your contact information so that she can reach you if necessary.

Data for the study will be collected and stored using a study code. Each participant will get a study code that replaces all personal data. Only the research team knows the code given to your data. In communication and reports about the study the study code will be used to protect your privacy.

Access to your data
To check whether the study is being performed according to the guidelines, some people may access the study data without using the code. Apart from the research team this concerns the monitor of the study and the Healthcare Inspectorate. They are obliged to keep your data secret. If representatives of Ipsen, the financier of the study and manufacturer of lanreotide, need to check the study, they only have access to the encoded data.

Storing of data
All data collected during this study needs to be stored for 15 years. If you sign the consent form, you consent to your medical and personal data being collected, stored and accessed. General information about the usage and storage of data can be found in the general brochure on medical research.

Storing of blood
During the study period blood tests are performed as part of your regular care. Part of the collected blood will be stored for a longer period than usual, also using the study code. We will store the blood in case we need to perform additional tests. The stored blood samples will only be used for this study. It will be destroyed once the study is concluded. The study is concluded once all the participants have completed the study and the results can be published. This will not be longer than 5 years. If you sign the consent form, you consent to the temporary storage of your blood samples.

10. Are you insured if you participate in this study?

Insurance has been taken out for everyone participating in this study. This insurance covers damage caused by the study. This applies to damage manifesting during the study or within four years of the end of the study. The insurance does not cover all damages. **Appendix E** contains more information about the insurance and the contact information.

11. Will my GP and/or my treating specialist be informed if I participate?

We will send your GP ("huisarts") and your treating internist-endocrinologist a letter to let them know that you are participating in the study. This is for your own safety. If you do not agree to this, you cannot participate in this study.

12. Is there a compensation when you participate?

The study medication, additional visits and PET/CT scan for the study are free of charge for you. You will not be paid for your participation in this study, but you will be reimbursed for your travel costs.

For peer review only - http://bmjopen.bmj.com/site/about/guidelines.xhtml

13. Which Ethics Committee has approved this study?

The Ethics Committee of the AMC has approved this study. General information about the assessment of research can be found in the general brochure on medical research.

14. Any questions?

You do not have to decide right away whether you want to participate in the study. In about a week we will get in touch with you to inform about your decision. If you have any questions or would like more information, please contact the coordinating investigator, Tessel Boertien. If you would like independent advice about participation in this study, you may contact the independent doctor M.R. Soeters, internist-endocrinologist. He knows about the study but is not involved in it. All contact details can be found in **appendix A**.

Thank you for your attention and kind regards,

T.M Boertien, MD, coordinating investigator and project leader
Prof. E. Fliers, MD PhD, internist-endocrinologist and principal investigator AMC

Appendices:

- Separate: Brochure ‘Medical research. General information for research participants’
- Appendix A: Contact details and hospital information
- Appendix B: Study overview
- Appendix C: Side effects of lanreotide – Somatuline AutoSolution
- Appendix D: Additional information on PET/CT and radiation exposure
- Appendix E: Insurance information
- Appendix F: Informed Consent form

For peer review only - http://bmjopen.bmj.com/site/about/guidelines.xhtml

APPENDIX A: INFORMATION ON THE PARTICIPATING CENTERS & CONTACT DETAILS

Coordinating investigator & project leader
T.M. Boertien, MD
PhD candidate Endocrinology AMC & VUmc
email: **t.m.boertien@amsterdamumc.nl**
Tel. **020 566 3507** / 020 566 6071 (secr.)

Study postal address: Academisch Medisch Centrum, t.a.v. T.M. Boertien
Afdeling Endocrinologie & Metabolisme, K2-283
Meibergdreef 9, 1105 AZ Amsterdam

Independent expert
M.R. Soeters, MD PhD, internist-endocrinologist
email: m.r.soeters@amsterdamumc.nl
Tel. 020 566 6071

Information for Academic Medical Center (AMC)
Address: Meibergdreef 9
1105 AZ Amsterdam
General no.: 020 566 9111
Nuclear medicine: 020 566 2775 (7:30-17:00)

In case of acute problems outside office hours you can call the general number of the AMC (020 566 9111) 24 hours a day and ask to be put through to the attending internist-endocrinologist. Always mention your participation in this study.

Principal investigator
Prof. E. Fliers, MD PhD, internist-endocrinologist
email: e.fliers@amsterdamumc.nl
Tel. 020 566 6071

Endocrine nurses
Moniek Gent-Houben and Shariefa Peters-Amanatkhan
e-mail fk.endocrinologie@amsterdamumc.nl
Tel. 020 566 2990

AMC department for patient information and complaints
The AMC department for patient information and complaints is at your service for general questions and information about matters such as participating in medical-scientific research. If you are not satisfied or unhappy with the care, treatment or services, you can inform the staff about your complaint. They can inform you about the procedure of filing a complaint and if necessary call in the complaints officer.

Location within AMC: outpatients clinic at the ground floor, A0-144

For peer review only - http://bmjopen.bmj.com/site/about/guidelines.xhtml

Opening hours:	Monday to Friday from 9:00 to 12:30 hrs
email:	patientenvoorlichting@amc.nl
Tel.:	020 566 3355
Postal address:	Academisch Medisch Centrum, t.a.v. patiëntenvoorlichting, A0-144<br>Postbus 22660, 1100 DD Amsterdam

Contact details Eurocept Homecare

Tel.: 035 528 8375 (24 hrs a day)

email galantstudie@eurocept.nl

For peer review only

For peer review only - http://bmjopen.bmj.com/site/about/guidelines.xhtml

Amsterdam UMC
Universitair Medische Centra

APPENDIX B: STUDY OVERVIEW

This is an overview of the study period. The study visit with **bold** numbers will definitely take place. In this example the PET/CT is planned during a separate visit (no. 2). It could be that this scan can already take place during visit no. 1. The 7th and the 13th injection will take place on a day that you also have a study visit at the AMC. The total amount of study visits depends on where the rest of the injections are administered: at home or at the AMC. That is why those visit numbers are between parentheses ( ). If all injections take place at the AMC there will be 21 visits in total.

RANDOMIZATION ↓

Time point →	start			6 mo.		12 mo.		18 mo.	END OF STUDY
Study visit no. →	**1**	**2**	(3 – 8)	**3** (9)	(10 – 14)	**4** (15)	(16 – 20)	**5** (21)	
Location →	AMC	AMC	AMC/home	AMC	AMC/home	AMC	AMC/home	AMC	
Duration in minutes →	90	90	15	90	15	60	15	75	

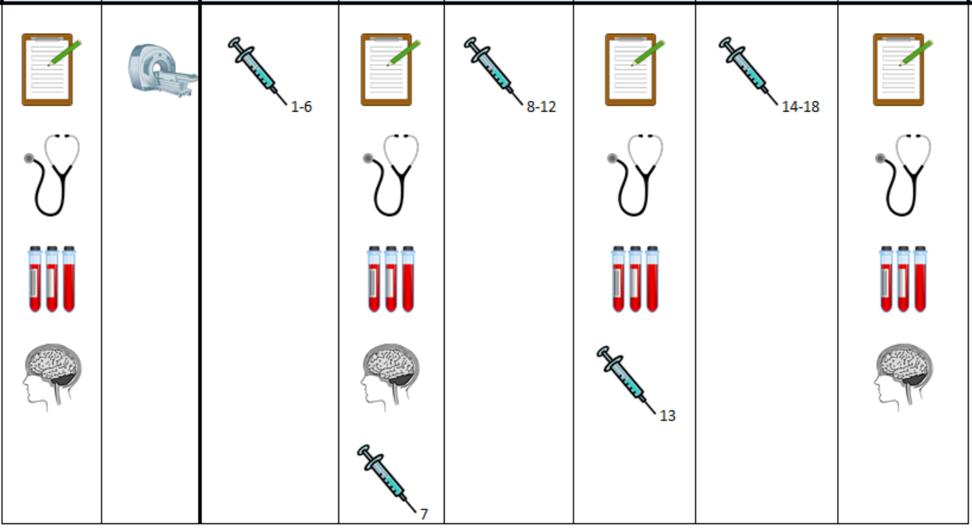

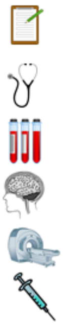

= interview and quality of life questionnaire

= short physical examination (blood pressure, heart rate, height, weight; if necessary more extensive)

= blood withdrawal (single puncture, 8 tubes, 36 milliliters)

= MRI scan of the brain

= PET/CT scan

= subcutaneous injection (lanreotide / Somatuline AutoSolution OR placebo)

For peer review only - http://bmjopen.bmj.com/site/about/guidelines.xhtml

APPENDIX C: SIDE EFFECTS OF LANREOTIDE – SOMATULINE AUTOSOLUTION

Like all medicines, Somatuline AutoSolution (the brand name of lanreotide) can cause side effects, although they do not affect everyone.

As mentioned earlier, the most commonly reported side effects are gastrointestinal complaints, gallbladder problems and injection site reactions. The list below shows the side effects that can occur with Somatuline AutoSolution according to their frequencies:

<u>Very common: may affect more than 1 in 10 people (>10%)</u>:

- Diarrhea, loose stools, abdominal pain
- Formation of gallstones; this normally doesn't cause symptoms. In some cases however a gallstone can lead to blockage of a bile duct. This can cause the following symptoms: severe and sudden abdominal pain, high fever, chills, jaundice (yellowing of the skin and whites of the eyes), loss of appetite, itchy skin

<u>Common: may affect up to 1 in 10 people (1-10%)</u>:

- Site reactions where the injection is given such as swelling, pain or itchiness
- Nausea, vomiting, constipation, gas formation/wind, stomach bloating or discomfort, indigestion, excess fat in stools
- Decrease in appetite
- Weight loss
- Feeling dizzy, headaches
- Pain that affects muscles, ligaments, tendons and bones
- Lack of energy or feeling tired
- Slow heart beat (sinus bradycardia)
- Loss of hair or less development of body hair
- Enlargement of the bile ducts between your liver and gallbladder and the intestine
- Lowered blood sugar level: this can cause symptoms of excessive sweating, feeling shaky, feeling hungry and confusion
- Heightened blood sugar level: this can cause symptoms of thirst, a dry mouth and feeling more tired than usual
- Abnormal liver and pancreas test results, shown in blood tests

<u>Uncommon: may affect up to 1 in 100 people (<1%)</u>:

- Hot flushes
- Difficulty sleeping
- A change in the color of the stools
- Changes to sodium and alkaline phosphatase levels, shown in blood tests

If any of these side effects becomes severe or if you experience a severe symptom that is not listed here, please contact the coordinating investigator or, if there is no answer or outside office hours, the attending internist-endocrinologist of your hospital - *see **appendix A** for contact details*

For peer review only - http://bmjopen.bmj.com/site/about/guidelines.xhtml

For peer review only

APPENDIX D: ADDITIONAL INFORMATION ON PET/CT & RADIATION EXPOSURE

General information

The PET/CT scan will be performed at the department of Nuclear Medicine of the AMC. This will either take place in the afternoon of the first visit or during a separate, second visit. You are allowed to eat and drink before the scan. At the department of Nuclear Medicine, a small intravenous line will be placed in your arm. A small amount of the slightly radioactive marker (the radionuclide Gallium-68 DOTATATE) will be administered through the line. Following the injection the line will be removed. It takes 45 to 60 minutes for the radionuclide to be distributed through the body. During this time you can take place in the waiting room or go and have something to eat or drink. Then you will be placed comfortably on the PET/CT table and scanned. During the scan the table is moving through a large ring that is open on both sides. The technician is present continuously and you can contact him or her through the intercom. The imaging session will take about 10 minutes. It is important that you remain as still as possible. After the scan you may go home directly and continue your usual activities. The marker loses its radioactivity very quickly; it carries no risks for your environment.

Radiation exposure

The PET/CT involves using radioactive markers. Radiation exposure is expressed in mSv (millisievert). The total amount of radiation you will be exposed to in this study is estimated at 3.1 mSv. To compare: the background radiation in the Netherlands is 2-2.5 mSv per year. If you participate in scientific research involving exposure to radiation more often, you should discuss with the investigator whether participation now would be safe. The total amount of radiation due to scientific research should not exceed 10 mSv in 5 years. Participation is advised against if you are exposed to an amount of radiation that exceeds the background radiation as part of your job.

What is the risk of radiation?

The body is made up of different cells. There are brain cells, muscle cells, blood cells etc. The genes inside these cells carry the genetic material and determine the function of the cells. X-rays or radiation from radioactive substances can damage these genes. This can lead to cancer or a congenital (inborn) disorder.

What are the chances of these severe consequences?

Cancer due to radiation usually is not detected until 20 years after the exposure. An exposure of 1 mSv generates a chance of 1 in 17,000 to develop cancer. With an exposure of 5 mSv this chance is increased to 1 in 3,400 and with an exposure of 10 mSv to 1 in 1,700. You need to compare these chances with the chance of 25% (1 in 4) to develop cancer that every human has.

If genes in the reproductive cells (sperm and eggs) are damaged, there is a risk of a congenital (inborn) disorder of the child. An exposure of 1 mSv radiation of the parent will be the cause of a severe congenital disorder in 1 in 77,000 babies. You need to compare this chance with the chance of 2,3% (23 in 1,000) of a congenital disorder that every baby has.

For peer review only - http://bmjopen.bmj.com/site/about/guidelines.xhtml

APPENDIX E: INSURANCE INFORMATION

Insurance has been taken out by AMC Medical Research B.V. for everyone participating in this study. The insurance covers damage due to participation in the study. This applies to damage manifesting during the study or within four years of the end of the study. The insurance offers a cover of € 450,000 per study subject, with a maximum of € 3,500,000 for the entire study and € 5,000,000 annually for all studies from the same sponsor. You must notify the insurance company about the damage within those four years. This is set out in the Medical Research (Human Subjects) Compulsory Insurance Decree. This decree is listed on the website of the Central Committee on Research Involving Human Subjects www.ccmo.nl.

The insurance policy covers the following damage:

- damage as a result of a risk of participation in the clinical trial that you were not informed about in the written information;
- damage as a result of a risk that you were informed about in the written information, but which proved more serious than foreseen;
- damage as a result of a risk that you were informed about in the written information, but which was deemed very unlikely to occur.

The insurance policy does **not** cover the following damage:

- damage due to your health problems not being alleviated or your health problems deteriorating further, in case participation in the clinical trial took place within the context of treatment of those health problems;
- damage to your health that would also have occurred if you had not participated in the study;
- damage as a result of an existing treatment method for research into existing methods of treatment;
- damage to descendants as a result of a negative effect of the study on you or your descendants;
- damage resulting from not or not entirely following directions or instructions.

The insurance shall cover losses suffered by natural persons only. In the event of damage please contact the insurance company directly:

Name company:	Centramed B.A.
Postal address:	Postbus 7374
	2701 AJ Zoetermeer
Telephone number:	070 301 7070
Email:	info@centramed.nl
Policy number:	620.872.806

Furthermore you are requested to contact the principal investigator of the study, prof. E. Fliers (tel. 020 566 6071).

For peer review only - http://bmjopen.bmj.com/site/about/guidelines.xhtml

APPENDIX F: INFORMED CONSENT FORM

GALANT: The effect of lanreotide on non-functioning pituitary macroadenoma (NFMA) size

ABR: NL52821.018.15 – protocol version 3.0, 13 October 2016

Official study title: A randomized placebo-controlled study in patients with a **Ga**llium-68 DOTATATE PET/CT positive, clinically non-functioning pituitary macroadenoma (NFMA) of the effect of **Lan**reotide autosolution on **T**umor (adenoma) size (**GALANT**)

I have read the subject information letter. I was also able to ask questions. My questions have been answered to my satisfaction. I had enough time to decide whether to participate.

I know that participation is voluntary. I know that I may decide at any time not to participate after all or to withdraw from the study. I do not need to give a reason for this.

I know that incidental findings about my health during this study will be discussed with me.

I give permission to inform my GP and my treating specialist about my participation in this study.

I know that my personal data will be registered at Eurocept Homecare if the injections take place at home.

I know that my contact information will be stored in a study file at the AMC so that the coordinating investigator can contact me about the study.

I know that the research team, the study monitor and representatives of the Healthcare Inspectorate can access my data without the study code.

I consent to the collection and usage of my data for the purposes stated in the information letter.

I consent to my data being stored for another 15 years after this study.

I consent to my blood samples being stored during the study until publication of the results (maximum of 5 years), after which it shall be destroyed.

I ☐ **do**
☐ **do not**
consent to being contacted again after this study for a follow-up study.

For peer review only - http://bmjopen.bmj.com/site/about/guidelines.xhtml

I want to participate in this study.

Name study subject: ………………………………………………………………………………….

Signature: ……………………………………………………………… Date: …………/…………/………….

I hereby declare that I have fully informed this study subject about this study.

If information comes to light during the course of the study that could affect the study subject's consent, I will inform him/her of this in a timely fashion.

Name investigator: …………………………………………………………………………………

Signature: ……………………………………………………………… Date: …………/…………/………….

Additional information was given by (if applicable):

Name: …………………………………………………………………………………….

Job title: ………………………………………………………………………………….

Signature: ……………………………………………………………… Date: …………/…………/………….

The study subject receives a complete information letter and a copy of the signed informed consent form.

For peer review only - http://bmjopen.bmj.com/site/about/guidelines.xhtml
